# Potential use of noncoding RNAs and innovative therapeutic strategies to target the 5’UTR of SARS-CoV-2

**DOI:** 10.2217/epi-2020-0162

**Published:** 2020-09-02

**Authors:** Antonella Baldassarre, Alessandro Paolini, Stefania Paola Bruno, Cristina Felli, Alberto Eugenio Tozzi, Andrea Masotti

**Affiliations:** ^1^Children’s Hospital Bambino Gesù-IRCCS, Research Laboratories; Multifactorial & Complex Phenotype Research Area, V.le di San Paolo 15, Rome 00146, Italy

**Keywords:** 5’UTR, COVID-19, GapmeRs, miRNAs, RNAi, SARS-CoV, SARS-CoV-2

## Abstract

After the increasing number of severe acute respiratory syndrome coronavirus 2 (SARS-CoV-2) infections all over the world, researchers and clinicians are struggling to find a vaccine or innovative therapeutic strategies to treat this viral infection. The severe acute respiratory syndrome coronavirus infection that occurred in 2002, Middle East respiratory syndrome (MERS) and other more common infectious diseases such as hepatitis C virus, led to the discovery of many RNA-based drugs. Among them, siRNAs and antisense locked nucleic acids have been demonstrated to have effective antiviral effects both in animal models and humans. Owing to the high genomic homology of SARS-CoV-2 and severe acute respiratory syndrome coronavirus (80–82%) the use of these molecules could be employed successfully also to target this emerging coronavirus. Trying to translate this approach to treat COVID-19, we analyzed the common structural features of viral 5’UTR regions that can be targeted by noncoding RNAs and we also identified miRNAs binding sites suitable for designing RNA-based drugs to be employed successfully against SARS-CoV-2.

After the first reports of a novel severe acute respiratory syndrome coronavirus 2 (SARS-CoV-2) in Wuhan city (Hubei, China) the WHO declared the COVID-19 outbreak a Public Health Emergency of International Concern and more recently a pandemic, as the number of cases all over the world has dramatically increased. As with other acute respiratory illnesses, the clinical presentation may vary including influenza-like symptoms to severe pneumonia or breathing difficulties and sometimes death, more frequent in older people or individuals with pre-existing diabetes and heart disease conditions (WHO, https://www.who.int/ith/diseases/sars/en/). Similarly to the severe acute respiratory syndrome coronavirus (SARS-CoV) outbreak in 2002, the scientific community is struggling to find novel therapies, as neither vaccines or specific treatments are currently available. Among the first therapeutic options that have been employed since the beginning we recall the antiviral therapies [1]. Several antiviral drugs such as Ribavirin (routinely employed to treat hepatitis C) [2], Nelfinavir or a combination of Lopinavir and Ritonavir (widely used as HIV protease inhibitors) [3] and Remdesivir (a monophosphoramidate nucleoside) [4] alone or in combination with chloroquine/hydroxychloroquine [5] are currently being assessed for the treatment of COVID-19. More recently, combinations of drugs (i.e., Bromhexine/Arbidol/IFN-α2b, Hydroxychloroquine/Azithromycin, etc.), biological drugs (i.e., convalescent plasma, mesenchymal stem cell (MSC) and MSC-derived exosomes, chimeric antigen receptor-natural killer (CAR-NK) cells, mRNA-1273, etc.), Chinese medicine drugs (i.e., YinHu QingWen decoction, Xiyanping injection) and supplementations with Vitamins C and D are currently under investigation (https://www.clinicaltrials.gov/). However, some results about the lopinavir-ritonavir treatment confirmed that the combined drugs are ineffective [6].

SARS-CoV-2 has a single-stranded positive-sense RNA genome [7] with a approximately 80% similarity with the human SARS-CoV genome [8,9] although the similarity between genes may vary (i.e., the spike [S] protein of SARS-CoV-2 exhibits ∼72% nucleotide sequence similarity with SARS-CoV).

Among all the genomic regions of the SARS-CoV genome that have been studied so far, we think that the 5’UTR region and specific portion of it should be considered crucial when devising novel therapeutic molecules also for SARS-CoV-2 infections.

## 5’UTR & the leader sequence of SARS-CoV

In the years following the outbreak of SARS-CoV, genomics, phylogeny, antigenic structure, immune response and potential therapeutic interventions have been reviewed [10]. Specific coding regions of the viral genome that encoded proteins fundamental for virus replication and transmission (i.e., replicases, S and M glycoproteins, envelope E protein, etc.) have been investigated as well. Some of these studies focused also on the structure and functional role of the 5’UTR region in determining coronavirus infection and replication. The 5’UTR (as well as the 3’UTR) genomic sequence is crucial for viral RNA replication and transcription [11]. In particular, a specific portion of 50–100 nucleotides at the 5’ end of the genome, referred as the ‘leader’ sequence, has been found also at the 5’ ends of all encoded transcripts (i.e., subgenomic mRNAs). This leader sequence and a *cis*-acting element termed transcription-regulatory sequence (TRS) immediately following the leader sequence, represent a unique feature of coronaviruses and some other viruses of the order Nidovirales [12].

Although the viral transcription mechanism is not fully understood, two major models have been proposed: a leader-primed transcription [13,14] or a discontinuous transcription during minus-strand synthesis [15,16]. Without going into details on these mechanisms, we outline the importance of the 5’UTR region and of the highly conserved leader sequence of coronaviruses especially when prompted to find therapeutic solutions for SARS-CoV-2.

**Figure 1.**
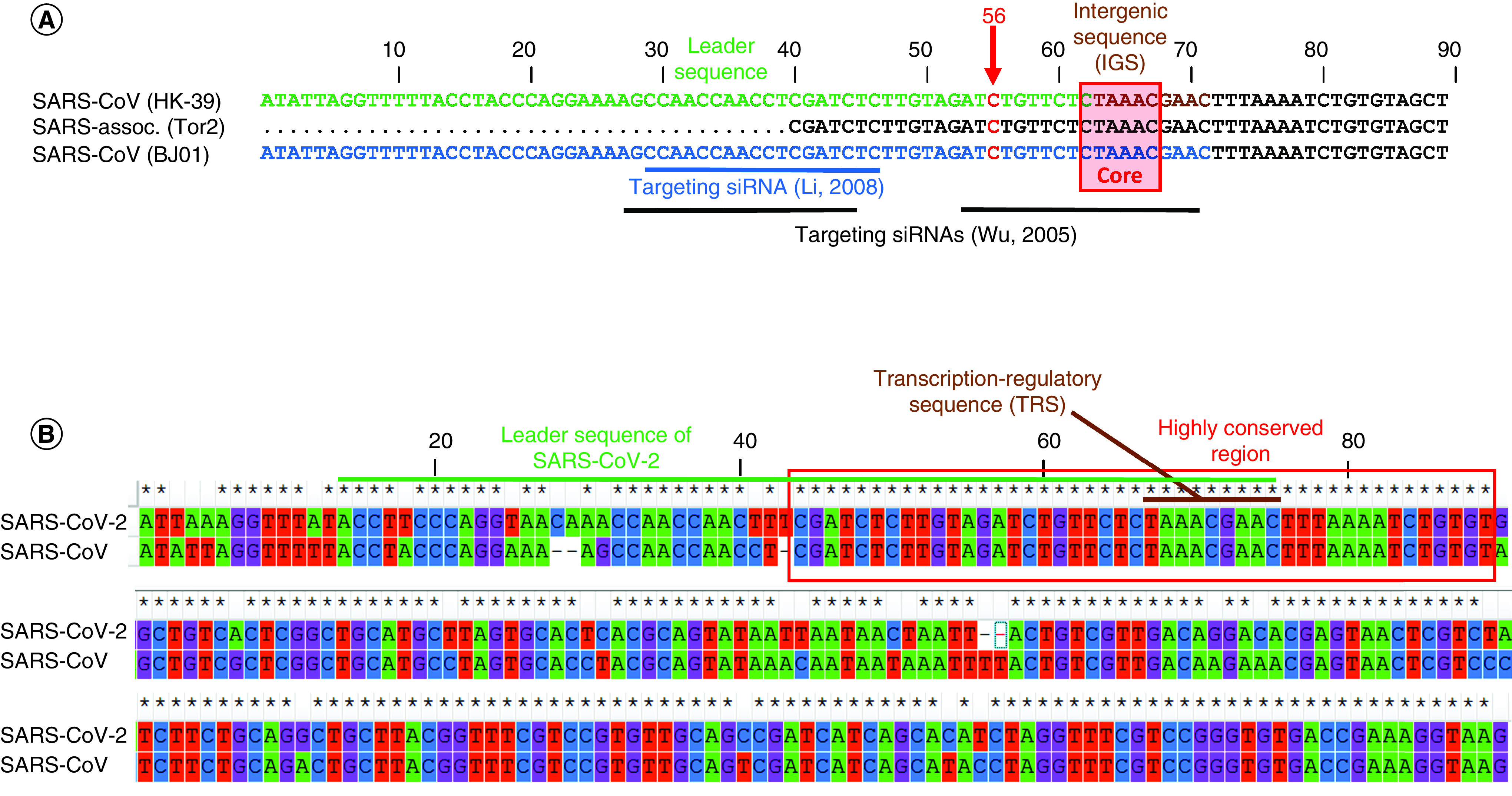
Alignment of 5'UTR regions of three coronaviruses. Alignment of **(A)** the first part (100 nucleotides) of the 5’UTR of three coronavirus isolates that show the similarity of the leader sequence (colored in green and blue) and the IGS depicted in brown. The position 56, considered crucial for viral transcription is represented in red. Blue and black lines indicate the genomic regions where siRNAs reported in the literature have been designed; **(B)** the alignment of complete 5’UTR region showing the similitude of the leader sequences and the high conservation of the transcription-regulatory sequence in SARS-CoV and SARS-CoV-2. IGS: Intergenic sequence; SARS-CoV: Severe acute respiratory syndrome coronavirus; SARS-CoV-2: Severe acute respiratory syndrome coronavirus 2; TRS: Transcription-regulatory sequence.

In fact, one of the most interesting aspects reported by Zeng and collaborators in 2003 was the identification of a leader sequence of 63 bp (Figure 1A) and an intergenic sequence (IGS) of 9 bp (5’-TAAACGAAC-3’) in the 5’UTR region of SARS-CoV HK-39 (NCBI Accession AY278491) [17]. Similarly, Li and collaborators reported a leader sequence of 72 bp for SARS-CoV BJ01 (NCBI Accession AY278488) coincident to that of SARS-CoV HK-39 (Figure 1A) [18]. Also in SARS-associated coronaviruses, such as the Tor2 isolate, the presence of a core sequence (5’-CTAAAC-3’) within the IGS region of the 5’UTR has been confirmed [19]. These regions have been indifferently termed IGS or TRS in different works, but the genomic location is the same.

Therefore, all of these data, while emphasizing the conservation of this genomic region among various coronavirus isolates, are useful to address the research toward the development of effective strategies aimed at reducing or abolishing viral activity and replication *in vitro* and *in vivo*. In fact, many strategies that we will describe in the following paragraphs have been adopted to target these viral genomic regions.

## RNAi against the 5’UTR & the leader region of SARS-CoV

RNAi was one of the strategies adopted in the past to inhibit the replication of coronavirus [20,21]. Several *in vitro* studies demonstrated that siRNAs targeting different region of SARS-CoV genome were effective in reducing the expression of viral subgenomic mRNAs, ultimately leading to an inhibition of viral replication. siRNA directed against the Spike region was the most effective although the authors demonstrated that also those siRNAs targeting the leader sequence or the TRS region and the 3’UTR were able to prevent SARS-CoV infection in Vero-E6 cells (Figure 1A) [20]. The SARS-CoV Spike protein was considered by many authors the most obvious candidate owing to its fundamental role in infecting human respiratory epithelial cells by interacting with human receptors (i.e., ACE2) [22]. Similarly, other studies reported the effective targeting of different regions such as the SARS-CoV M gene [23].

However, Li *et al.* by employing a specific siRNA (outlined in Figure 1A) were able to target the SARS-CoV leader sequence and effectively inhibit virus replication in Vero E6 cells [24].

However, a detailed study demonstrated that specific siRNA duplexes (i.e., siSC2 and siSC5), targeting the SARS-CoV Spike and *ORF1b* (*NSP12*) regions can significantly suppress SARS-like symptoms also *in vivo* (i.e., in infected macaques) [24]. More importantly, the RNAi approach displayed no adverse effects and was demonstrated to be effective for both prophylaxis and therapy [25]. As we were aimed mainly at reporting only those RNAi strategies directed to target the 5’UTR, we preferred to omit general review papers (i.e., [21] and [26]) dealing with targeting other genomic regions.

Although this approach cannot be considered an antiviral approach (as animals developed symptoms), it is surely a valid strategy to reduce the viral load and the severity of the disease. In any case, the idea that RNAi can be used to mitigate the symptoms of new coronavirus infections and other emerging infectious diseases was a debated concept in the past [27], but it is still actual and valuable of further investigations. In fact, we believe that approaches based on the common features shared between SARS-CoV and SARS-CoV-2 could be employed to block the virus replication.

## 5’UTR & the leader sequence of SARS-CoV-2

To evaluate whether the concepts already found for 5’UTR of SARS-CoV can be transferred also to SARS-CoV-2, we calculated the similarity between the 5’UTR of the two coronaviruses. We found that the similarity of these two regions is 88.76% (Figure 1B) and sequence alignment emphasized the presence of many conserved genomic regions between SARS-CoV and SARS-CoV-2 5’UTRs, especially in the first 90 nucleotides. Interestingly, the regions encompassing the TRS of both SARS-CoV and SARS-CoV-2 are conserved in all known genome sequences (Supplementary data). Owing to their high similarity, the region that encompasses the TRS (i.e., spanning between 40 and 85 nucleotides) is identical in SARS-CoV and SARS-CoV-2 (Figure 1B) and conserved (Supplementary data). More recently, a preliminary work of Taiaroa and his group reports the use of the leader sequence of SARS-CoV-2 for the identification of subgenomic mRNAs [28]. This sequence is very similar to that of SARS-CoV (Figure 1B) and this strongly suggests that the RNAi strategy already adopted for SARS-CoV and focused on this specific region would likely lead to inhibition of SARS-CoV-2 replication. However, RNAi is not the only strategy that can be employed to this purpose.

## Deletion of specific regions in the 5’UTR of SARS-CoV

In 2006, to test the promoter activity of the 5′UTR of SARS-CoV in eukaryotic cells and identify the fundamental regions necessary for viral replication, Zhang and collaborators assessed the function of many 5’UTR clones lacking increasing portions of the SARS-CoV 5’UTR sequence [29]. They found that the wild-type SARS-CoV 5’UTR has a promoter activity in eukaryotic cells such as A549, HepG2, ECV304, HeLa and Vero E6 and that deletion mutant plasmids with different 5’UTR length have different activities. Interestingly, the mutant plasmid lacking the region 1–36 did not altered the activity, whereas the plasmid lacking almost all the UTR (i.e., the 1–222 region) abolished completely the SARS-CoV promoter activity in human cells. In fact, it is long been known that subgenomic mRNAs lacking the 5’ leader sequence are not able to replicate [30,31]. At least four stem loop structures are located in this 5’-end region of the coronavirus genome and these secondary structures are actively implicated in viral replication and transcription [32]. Moreover, the authors identified the initial site of transcription at the 56th nucleotide (see Figure 1A) that is proximal to the TRS sequence and to those regions already targeted by siRNAs. It is reasonable to think that this position is among the crucial genomic regions to consider for targeting purposes.

## Secondary structures of coronaviruses 5’UTR

**Figure 2.**
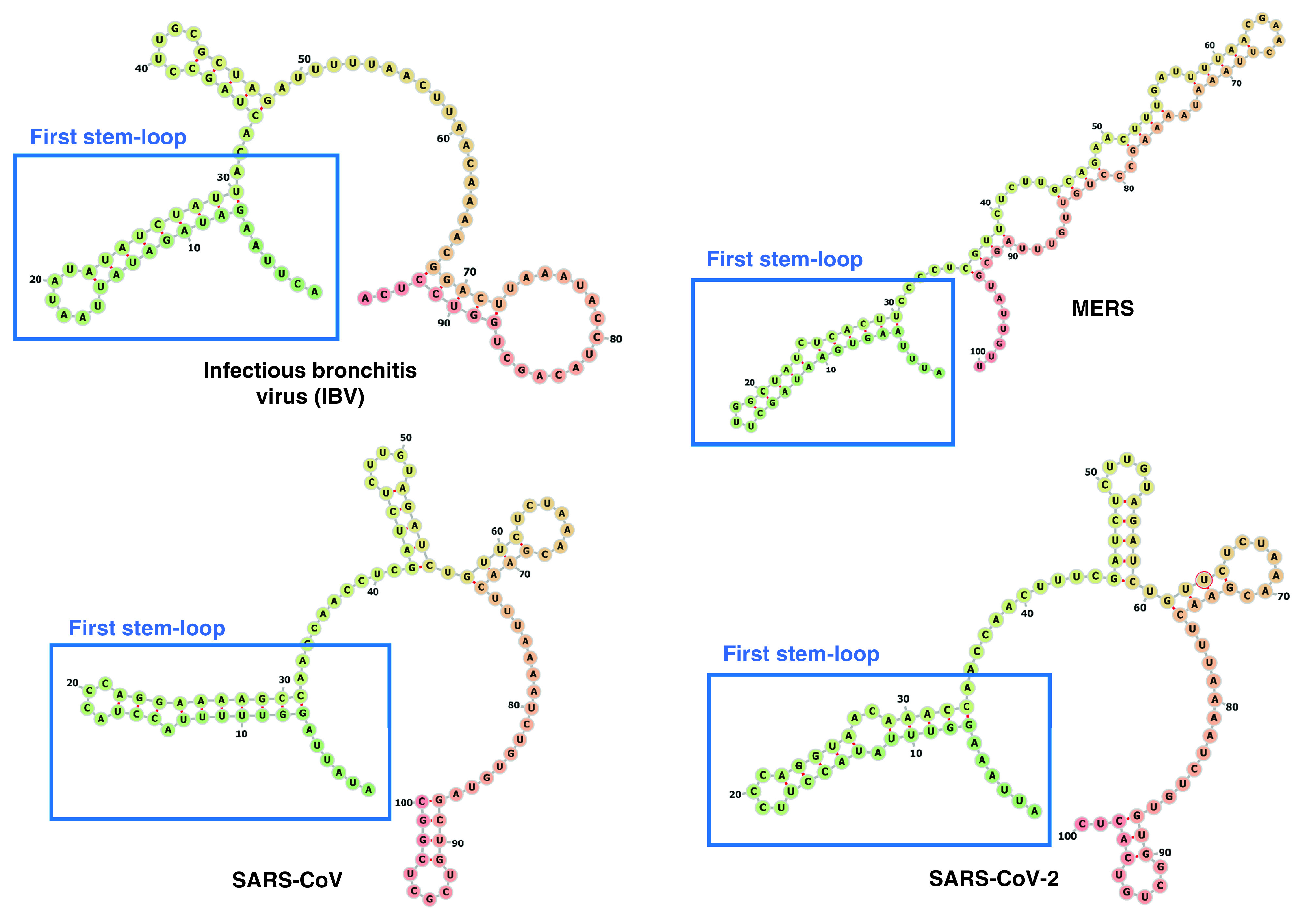
Secondary structures of the first 95–100 nucleotides of common coronavirus such as infectious bronchitis virus, MERS, severe acute respiratory syndrome coronavirus and severe acute respiratory syndrome coronavirus 2 showing the structural similarity of the first stem loop structure.

**Figure 3.**
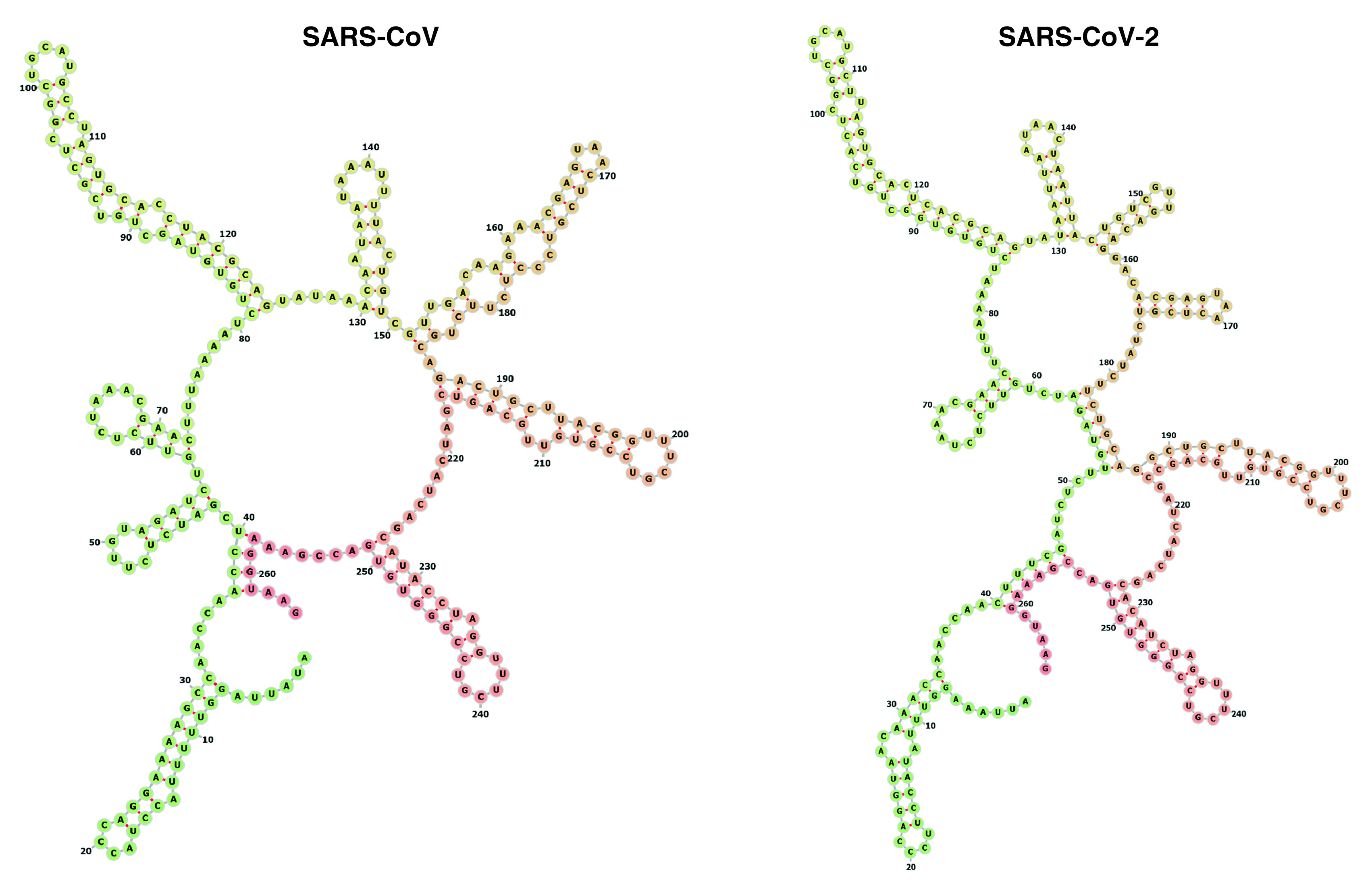
Secondary structures of the 5’UTR region of severe acute respiratory syndrome coronavirus and severe acute respiratory syndrome coronavirus 2 that emphasize the structural similarity of these two coronaviruses.

The RNA synthesis processes of coronaviruses takes place in the cytoplasm and is regulated by proteins of the host cell. Among these proteins, the zinc-finger and RNA binding motif MADP1 was able to interact with the 5’UTR region of the SARS-CoV genome and this interaction was confirmed also in coronavirus infectious bronchitis virus [33]. Interestingly, the interaction seems mediated by the secondary structure of the 5’UTR region and in particular by the presence of several stem-loops in this 5’end of the viral genome. After synthesizing four truncated biotin-labeled mutant RNA fragments of the 5’UTR region, the authors studied the minimal region required for MADP1 binding. Results indicated that even the lack of the first stem loop structure, located within the first 30 nucleotides from the beginning of the 5’UTR, is enough to abolish the binding of MADP1 protein. The absence of this protein determined a defective viral RNA synthesis *in vitro* but outlined the importance of the secondary structure of the 5’UTR region in coronavirus RNA synthesis. The presence of these stem-loops is highly conserved in many coronaviruses derived from all three major CoV groups [34]. Moreover, a couple of papers pointed to the importance of single-nucleotide mutations and deletions that are able to destabilize the first two stem loops in the 5’UTR of coronaviruses, thus inhibiting viral replication [35,36]. By analyzing the first 100-nucleotide region of the 5’UTR of infectious bronchitis virus, MERS, SARS-CoV and SARS-CoV-2, we confirmed their similarity (Figure 2) and we hypothesized that also their function could be almost identical (i.e., enhancement of viral replication) [11]. However, this similarity extends also to the entire 5’UTR of SARS-CoV and SARS-CoV-2 (Figure 3).

Therefore, all of these data outline once more the importance of 5’UTR when devising therapeutic strategies for coronaviruses.

## Locked nucleic acid antisense oligonucleotides

RNAi is not the only strategy that can be adopted to target the 5’UTR of coronaviruses. A few years ago an innovative antiviral strategy for hepatitis C virus (HCV), another common widespread virus, based on a locked nucleic acid (LNA)–modified DNA phosphorothioate antisense oligonucleotide, named Miravirsen, has been proposed [37]. This approach was based on a high affinity antisense oligonucleotide complementary to the human mature miR-122, a liver-specific and highly expressed microRNA. This microRNA is able to bind the 5’UTR of the HCV genome in two distinct regions and to promote actively the propagation of HCV RNA [38]. Prolonged subcutaneous administration of Miravirsen (at a dose of 3–7 mg per kg of body weight) halted the activity of miR-122 by preventing its binding to HCV 5’UTR and provided viral suppression up to the end of the therapy. This approach is based on two main pillars: the identification of miRNA binding sequences in the 5’UTR and the presence of the binding miRNA in the target tissue (i.e., the liver in the case of HCV) to boost viral propagation.

**Figure 4.**
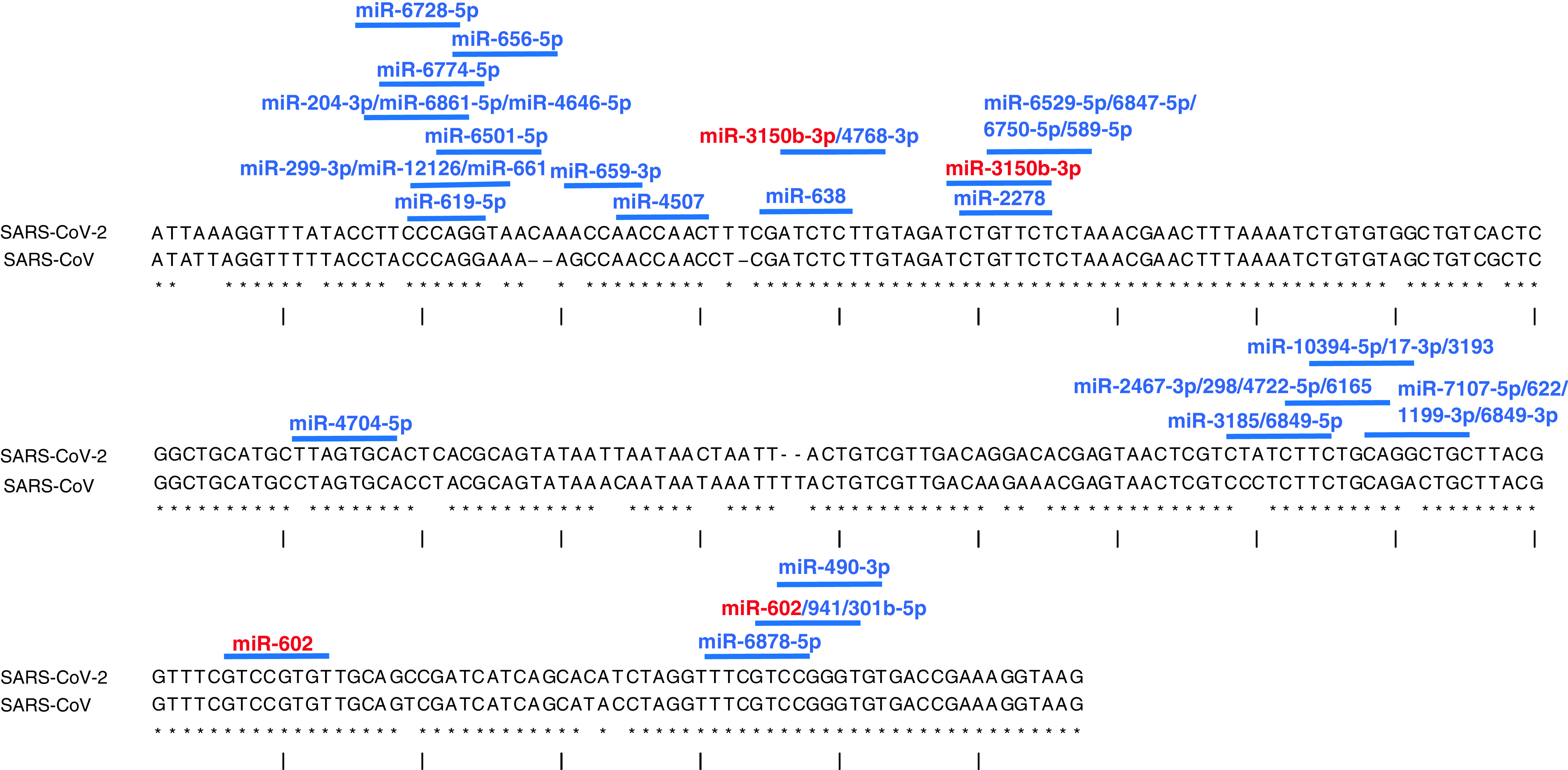
Aligned 5’UTR of severe acute respiratory syndrome coronavirus and severe acute respiratory syndrome coronaviruses 2 and the identification of miRNA binding sites. miRNAs targeting one site are depicted in blue, whereas in red if they target two sites (such as miR-3150-3p and miR-602).

To translate this approach into the identification of therapeutic molecules for the potential treatment of COVID-19 infections, we searched for potential miRNAs binding sites targeting the 5’UTR of SARS-CoV-2. Target recognition algorithms such as PITA [39] allowed identifying the accessible genomic binding sites for human miRNAs. Many miRNA binding sites were identified in conserved regions (Figure 4) that were filtered according to their score (ddG cutoff ≤10; Supplementary data). We would like to emphasize that this score is only an indicative *in silico* prediction score and does not necessarily reflects the real hybridization behavior in *in vitro* or *in vivo* studies. In any case, this score is a good starting point for further experimental considerations. Through this approach, up to 41 sites for miRNA binding were identified on the 5’UTR of SARS-CoV-2. Of note, several miRNAs such as miR-4507, miR-638, miR-3150b-3p and miR-602 can bind conserved regions of the 5’UTR of SARS-CoV-2. In HCV infection, miR-122 is highly expressed in the liver and this organ represents the primary target site of the virus. Similarly, we focused to find highly expressed miRNAs in the lungs that are the main target organs for SARS-CoV-2. Therefore, we extracted and analyzed the expression data contained in the human miRNA tissue atlas [40] to gain information about the quantity of these miRNAs in human tissues. We found that miR-4507 was among the most expressed miRNAs (∼99th percentile) in the lungs together with miR-638 (∼97th percentile) which is highly expressed also in the pleura (∼99th percentile). Two distinct binding regions were found in the 5’UTR of SARS-CoV-2 genome for miR-3150b-3p and miR-602. We still do not know if these miRNAs have crucial functions in promoting viral replication or if the binding of other miRNAs in different 5’UTR region has inhibitory functions. If we suppose a similar enhancing mechanism, we should hypothesize that the design of antisense oligonucleotides similar to Miravirsen could be able to sequester these miRNAs and inhibit viral replication. In any case, these findings prompt the research to validate these targets, explore their function and potentially identify novel drugs.

However, many other miRNAs resulted to bind coding regions and 3’UTR of the coronavirus (Supplementary data) where other *cis*-acting region are present, although the regulatory mechanisms underlying these interactions are still not completely understood [41]. The RNA-based antisense therapy is of wide applicability and the strategy herein suggested was already assessed in chronic HCV infections with no major adverse events. Based on these findings, we recommend to consider the possibility to employ miRNAs and their antisense analogues as effective candidates for targeting coronaviruses and to identify novel molecules for antiviral therapy.

## RNA-based drugs in viral infection

Many antiviral strategies have been devised in the last few years to reduce or abolish the activity of RNA viruses. In particular, drug development was focused to target not only two families of positive strand RNA viruses, the *Flaviviridae* [42] and the *Coronaviridae* [43], but also the families of negative strand RNA families such as *Filoviridae* [44] and *Rhabdoviridea* [45].

For *Coronaviridae* family, the development of antiviral nucleoside and nucleotide analogues targeting viral RNA synthesis (i.e., Gemcitabine, Remdesivir, Ribavirin, 6-Azauridine and Mizoribine) have been proposed as effective therapeutics against CoVs infection [43] and further assessed as antivirals against *Flaviviridae* and *Filoviridae* families together with molecules such as BXC4430, favipiravir and 3-deazaneplanocin A. However, the efforts and the development stage of these approaches are different as recently reported in an editorial paper by Brinton and colleagues [46].

One of the main problems associated to the delivery of RNA-based drugs is the intracellular presence of RNAses that can rapidly degrade the delivered cargo thus reducing the efficacy of the treatment [47]. Therefore, many efforts have been dedicated to improve the RNA stability and the delivery efficiency by exploiting encapsulation strategies such as the use of lipids, liposomes, polymers and nanoparticles [48], by targeting dendritic cells with liposomal vaccines [49] or by self-replicating RNA molecules based on RNA viruses [50].

Another less common antiviral approach is the exploitation of a fundamental property of ssRNA viruses: the extensive cytoplasmic RNA replication that leads to an extreme transgene expression. It has been demonstrated that alphaviruses, flaviviruses, rhabdoviruses and measles viruses can be employed for immunization against several infectious agents, thus providing a strong immune response against challenges with lethal viral loads [51]. In fact, mice vaccinated with Venezuelan equine encephalitis virus particles encoding the SARS-CoV spike glycoprotein resulted protected against subsequent challenges with lethal doses of the coronavirus [52].

Encapsulation of nucleoside-modified RNA molecules within lipid-nanoparticles is another relatively new approach already employed successfully in the immunization of mice and nonhuman primates against Zika virus [53] an approach that can be easily extended also to other viruses. Interestingly, the incorporation of RNA molecules within nanoparticles to form lipoplexes have already reached the clinical stage [54] although the main use is for tumor therapy as in the case of the RNA-lipoplex formulation lipoMERIT (a Phase I study) for advanced melanoma treatment in humans (ClinicalTrials.gov Identifier: NCT02410733).

In viral infection, RNA-based drugs are in different clinical Phase trials [55]. For example, many mRNA-based drugs are in Phase I in the area of infectious diseases against influenza, Zika and Chikungunya viruses (Moderna Therapeutics, MA, USA; www.modernatx.com/pipeline), whereas RNAi-loaded lipid nanoparticles targeting chronic hepatitis B virus are actually in Phase II (Arbutus Biopharma, PA, USA; http://www.arbutusbio.com/portfolio/rd-portfolio.php).

## LNA-based oligonucleotides for *in vivo* applications (GapmeRs)

**Figure 5.**
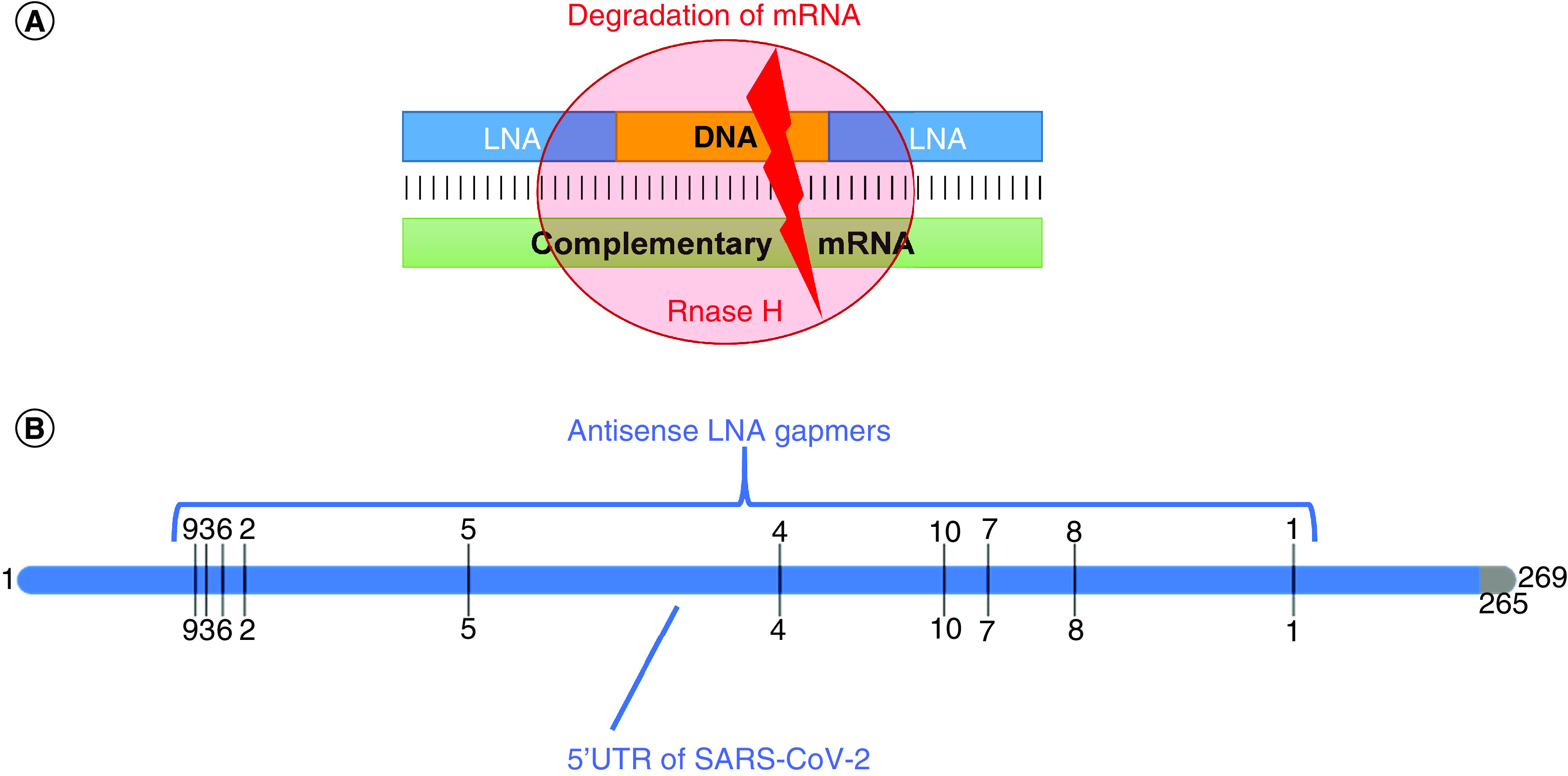
Antisense LNA GapmeRs. Schematic representation of **(A)** the functioning of GapmeRs oligonucleotides made by a DNA oligonucleotide 5’- and 3’-linked to two LNA terminals that is able to activate the degradation of mRNA by RNase H; **(B)** GapmeRs indicated with numbers have been designed starting from the 5’UTR sequence. LNA: Locked nucleic acid.

In the last few years, novel single-stranded antisense oligonucleotides, named GapmeRs, designed to silence mRNA and other long noncoding RNAs *in vitro* and *in vivo* appeared on the market. These oligonucleotides have particular properties imparted by the linking of the 2’-O and 4’-C atoms of the ribose ring that lead to a ‘locked’ conformation, which is ideal for Watson-Crick pairing. The antisense ‘locked’ (or ‘bridged’) nucleic acid (LNA or bridged nucleic acid [BNA], respectively) GapmeRs can pair more rapidly with a complementary nucleotide strand and the stability of the resulting duplex is increased compared with traditional oligonucleotides. GapmeRs are generally designed to have a DNA portion flanked by LNA (Figure 5A). Duplexes of DNA hybridized to RNA generally catalyze RNase H-dependent degradation of the RNA strand, whereas LNA does not activate RNase H. For this reason, antisense LNA GapmeRs are able to cleave efficiently the target RNA.

Despite the advantages of using siRNAs or GapmeRs to treat viral infections, we have to mention also the potential pitfalls associated with these approaches [56]. One of the most common issues, especially for the ‘first-generation’ antisense oligonucleotides, is off-target effects that are the unintentional binding of RNA oligonucleotides to cell surface proteins or to intracellular proteins (i.e., Toll-like receptors). This lack of hybridization selectivity might activate the innate immune response [57]. Another off-target effect caused by an ineffective hybridization could be the recognition of DNA/RNA molecules that are not the intended target of the designed siRNA/GapmeR. Again, this binding might consequently modulate the expression of genes with other physiological (and undesired) effects. Finally, dsRNAs introduced massively into cells may co-opt or interfere with the endogenous machinery of RNAi and block for example the normal function of miRNAs [58].

A recent study reported that RNase H1-dependent delocalization of paraspeckle proteins to nucleoli is an early event in the toxicity displayed by modified antisense oligonucleotides [59]. This event is followed by nucleolar stress, p53 activation and apoptotic cell death. In the past, all of these side effects, coupled to kidney and/or liver toxicity, have severely limited the clinical application of RNA-based drugs [60–62]. Interestingly, one recent study reported that the introduction of a single 2’-O-methyl modification at the second nucleoside in the DNA gap reduced protein-binding and substantially decreased hepatotoxicity and improved the therapeutic index with minimal impairment of antisense activity [59].

LNA GapmeRs, originally designed for silencing lncRNAs and mRNAs *in vitro* and *in vivo*, have been extended also to study the inhibition of virus replication. In fact, specific LNA GapmeRs have been designed and employed to target the 5’UTR region of coxsackievirus B3 [63] and the HCV internal ribosome entry site containing the distal and proximal miR-122 binding sites [64]. We outline that these are the only two studies reported in the literature employing GapmeRs as innovative drugs for viral therapy and are both done *in vitro*. So far, no *in vivo* experiments have been conducted with GapmeRs in antiviral therapy and this strengthen the possibility to accelerate research and discover innovative molecules in the future. In order to identify GapmerRs able to target the 5’UTR region of SARS-CoV-2 we interrogated the free tool online GeneGlobe (https://geneglobe.qiagen.com) and we identified many regions of the 5’UTR where the putative GapmeRs could be designed (Figure 5B). These GapmeRs could elicit RNAse H and induce a degradation of the viral RNA that in some cases would determine a loss of substantial parts of the 5’UTR of SARS-CoV-2 (i.e., GapmeR no. 1 in Figure 5B).

These data emphasize that GapmeRs could be optimized (i.e., proper binding region, sequence length, prevention of off-target effects) and effectively employed to inhibit the replication activity of SARS-CoV-2.

## Conclusion

We have summarized and discussed here the panorama of noncoding RNA molecules that can be employed to target the 5’UTR region of SARS-CoV-2 with the aim to find novel drugs or innovative therapeutic strategies (Table 1).

**Table 1.** List of the relevant literature papers cited in the present manuscript.

Topic	Ref.
**5’UTR and the leader sequence of SARS-CoV**	
Definition of the 5’UTR and the leader regions	[11,12]
Viral transcription mechanisms	[13–17,19,24]
**RNAi against SARS-CoV**	
siRNAs against the 5’UTR and the leader region	[20,21,24]
siRNAs against Spike or M proteins	[22,23]
**5’UTR and the leader sequence of SARS-CoV-2**	[28]
**Deletion of specific regions in the 5’UTR of SARS-CoV**	[29–32]
**Secondary structures of coronaviruses 5’UTR**	
Stem-loop regions and viral replication	[33–36]
**Locked nucleic acid antisense oligonucleotides**	
Miravirsen against the miR-122 encoding region in HCV	[37,38]
**RNA-based drugs in viral infection**	[46,47,55]
Nucleoside and nucleotide analogues for:	
- *Flaviviridae*	[42]
- *Coronaviridae*	[43]
- *Filoviridae*	[44]
- *Rhabdoviridea*	[45]
Encapsulated RNA-based molecules	[48,49]
Self-replicating RNA molecules	[50–52]
Encapsulated nucleoside-modified RNA molecules	[53,54]
**LNA-based oligonucleotides for *in vivo* applications (GapmeRs)**	[63,64]
Oligonucleotide-based drugs on the market	[65–81]

HCV: Hepatitis C virus; LNA: Locked nucleic acid; SARS-CoV: Severe acute respiratory syndrome coronavirus; SARS-CoV-2: Severe acute respiratory syndrome coronavirus 2.

Recently, a couple of papers emphasized the directions of RNAi therapeutics and their possible use as a tool to control viral infections [26,82]. Moreover, eight RNAi oligonucleotide-based drugs are already on the market for the treatment of several human diseases: givosiran for the treatment of acute intermittent porphyria [65], patisiran [66–69] and inotersen [70] for amyloidosis, nusinersen for spinal muscular atrophy [71,72], eteplirsen for Duchenne muscular dystrophy [73,74], defibrotide for severe veno-occlusive diseases [75–77], mipomersen for hypercholesterolemia [78,79] and pegaptanib in ophthalmology [80,81].

The process to commercialize a novel drug is lengthy and costly owing to the regulatory processes related to preclinical and clinical studies. Despite this, we think that many of the molecules already studied and developed after the previous SARS-CoV infection in 2002 can be the starting point to develop effective molecules also for the recent SARS-CoV-2 infection. One of the interesting aspects of these molecules is the possibility to alter not only the functioning of the viral replication machinery but also to impair the secondary structure of the crucial 5’UTR genomic region. However, many aspects must be considered to optimize this approach: the amount of drugs in relation to the viral genome to target, the presence of mutated viruses (i.e., quasispecies) that may be implicated in viral escape [83] and the exact localization of the delivered drug (i.e., nuclear or cytoplasmic depending on the chemical functionalization of the RNA-based drug). Many of the molecules described in this work are natural RNA molecules or already proved to display no toxicity *in vivo* and to enter into cells without the need of delivery vehicles or transfecting agents (i.e., GapmeRs). This is an advantageous property as generally RNA molecules are not very effective *per se.* In fact, they require a mean to reach the cells, such as in the case of the recent Phase I clinical trial on COVID-19 patients that employs mRNA-1273 delivered by lipid nanoparticles (sponsored by the NIAID, NCT04283461).

Independently by the use of siRNAs, miRNAs or GapmeRs, the arsenal that can be employed to fight SARS-CoV-2 is promising and potentially very powerful. In principle, some of these therapeutic options could alleviate COVID-19 symptoms or reduce viral replication. We strongly believe that none of these possibilities should be left unassessed, as the discovery of an effective vaccine could be a very long and uncertain path.

## Future perspective

One of the possible future directions in the optimization of RNA-based drug efficiency and in the development of novel therapeutic molecules could be focusing the research on the chemistry of these antisense molecules. In particular, designs employing chemistries other than the phosphorothioates (i.e., 2’-fluoroarabinonucleic acids) have shown promises in the improvement of affinity and stability of RNase H–oligonucleotide duplex [84]. Moreover, also the presence of stereoisomers in the modified phosphorothioate backbone (i.e., stereoselective GapmeRs) may have an effect in modulating the efficiency of these molecules [85]. Finally, another class of modified RNA-based drugs, referred as steric-blockers, are emerging in the arsenal of therapeutic molecules as they can alter splicing (i.e., nusinersen and eteplirsen for the treatment of spinal muscular atrophy and Duchenne muscular dystrophy, respectively), without activating RNase H, although their protein binding pattern and their reduced toxicity compared with GapmeRs remain to be fully elucidated.

**Executive summary**

**The importance of viral 5’UTR**

- The 5’UTR region is crucial to design novel antiviral molecules.
- The 5’UTR region encodes for a ‘leader’ sequence important for viral replication.
- The 5’UTR of severe acute respiratory syndrome coronavirus (SARS-CoV) can be effectively targeted by RNA-based drugs (i.e., siRNAs).
- The 5’UTR and the leader sequence of severe acute respiratory syndrome coronavirus 2 are very similar to those of SARS-CoV.
- The secondary structure of SARS-CoV 5’UTR is very similar to that of severe acute respiratory syndrome coronavirus 2.

**Antiviral strategies**

- Deletions or RNAi within the 5’UTR region can suppress SARS-CoV replication.
- Locked nucleic acids and GapmeRs are two innovative RNA-based molecules that showed promises as antiviral molecules.
