# Supplementary material for: Potential use of noncoding RNAs and innovative therapeutic strategies to target the 5’UTR of SARS-CoV-2
Source: Epigenomics. 2020 Sep 2:10.2217/epi-2020-0162. doi: 10.2217/epi-2020-0162 (PMC7466951; doi:10.2217/epi-2020-0162)
Supplement: Supplementary file 1 [file epi-2020-0162-supp-table-1.pdf]

```
Reference sequence (1): AP006557.1_SARS_coronavirus_TWH_genomic_RNA_complete_genome
Identities normalised by aligned length.
Colored by: identity

                                                                                            cov    pid   1 [         .         .         .         .         :         .         .         . 80
  1 AP006557.1_SARS_coronavirus_TWH_genomic_RNA_complete_genome                          100.0% 100.0%     ----------------------------------ATATTAGGTTTTTACCTACCCAGGAAAAGCCAACCAACCTCGATCT
  2 AP006558.1_SARS_coronavirus_TWJ_genomic_RNA_complete_genome                          100.0% 100.0%     ----------------------------------ATATTAGGTTTTTACCTACCCAGGAAAAGCCAACCAACCTCGATCT
  3 AP006559.1_SARS_coronavirus_TWK_genomic_RNA_complete_genome                          100.0% 100.0%     ----------------------------------ATATTAGGTTTTTACCTACCCAGGAAAAGCCAACCAACCTCGATCT
  4 AP006560.1_SARS_coronavirus_TWS_genomic_RNA_complete_genome                          100.0% 100.0%     ----------------------------------ATATTAGGTTTTTACCTACCCAGGAAAAGCCAACCAACCTCGATCT
  5 AP006561.1_SARS_coronavirus_TWY_genomic_RNA_complete_genome                          100.0% 100.0%     ----------------------------------ATATTAGGTTTTTACCTACCCAGGAAAAGCCAACCAACCTCGATCT
  6 AY274119.3_Severe_acute_respiratory_syndrome-related_coronavirus_isolate_Tor2_complete_genome 100.0% 100.0%     ----------------------------------ATATTAGGTTTTTACCTACCCAGGAAAAGCCAACCAACCTCGATCT
  7 AY278487.3_SARS_coronavirus_BJ02_complete_genome                                     100.0% 100.0%     ----------------------------------ATATTAGGTTTTTACCTACCCAGGAAAAGCCAACCAACCTCGATCT
  8 AY278488.2_SARS_coronavirus_BJ01_complete_genome                                      93.7%  93.7%     -----------------------------------------------------CCAGGAAAAGCCAACCAACCTCGATCT
  9 AY278489.2_SARS_coronavirus_GD01_complete_genome                                      94.7%  94.7%     --------------------------------------------------TACCCAGGAAAAGCCAACCAACCTCGATCT
 10 AY278490.3_SARS_coronavirus_BJ03_complete_genome                                      98.7%  98.7%     --------------------------------------TAGGTTTTTACCTACCCAGGAAAAGCCAACCAACCTCGATCT
 11 AY278491.2_SARS_coronavirus_HKU-39849_complete_genome                                100.0% 100.0%     ----------------------------------ATATTAGGTTTTTACCTACCCAGGAAAAGCCAACCAACCTCGATCT
 12 AY278554.2_SARS_coronavirus_CUHK-W1_complete_genome                                   95.0%  95.0%     -------------------------------------------------CTACCCAGGAAAAGCCAACCAACCTCGATCT
 13 AY278741.1_SARS_coronavirus_Urbani_complete_genome                                   100.0% 100.0%     ----------------------------------ATATTAGGTTTTTACCTACCCAGGAAAAGCCAACCAACCTCGATCT
 14 AY279354.2_SARS_coronavirus_BJ04_complete_genome                                      94.7%  94.7%     --------------------------------------------------TACCCAGGAAAAGCCAACCAACCTCGATCT
 15 AY282752.2_SARS_coronavirus_CUHK-Su10_complete_genome                                 95.0%  95.0%     -------------------------------------------------CTACCCAGGAAAAGCCAACCAACCTCGATCT
 16 AY283794.1_SARS_coronavirus_Sin2500_complete_genome                                   94.7%  94.7%     --------------------------------------------------TACCCAGGAAAAGCCAACCAACCTCGATCT
 17 AY283795.1_SARS_coronavirus_Sin2677_complete_genome                                   94.7%  94.7%     --------------------------------------------------TACCCAGGAAAAGCCAACCAACCTCGATCT
 18 AY283796.1_SARS_coronavirus_Sin2679_complete_genome                                   94.7%  94.7%     --------------------------------------------------TACCCAGGAAAAGCCAACCAACCTCGATCT
 19 AY283797.1_SARS_coronavirus_Sin2748_complete_genome                                   94.7%  94.7%     --------------------------------------------------TACCCAGGAAAAGCCAACCAACCTCGATCT
 20 AY283798.2_SARS_coronavirus_Sin2774_complete_genome                                   94.7%  94.7%     --------------------------------------------------TACCCAGGAAAAGCCAACCAACCTCGATCT
 21 AY291315.1_SARS_coronavirus_Frankfurt_1_complete_genome                              100.0% 100.0%     ----------------------------------ATATTAGGTTTTTACCTACCCAGGAAAAGCCAACCAACCTCGATCT
 22 AY291451.1_SARS_coronavirus_TW1_complete_genome                                      100.0% 100.0%     ----------------------------------ATATTAGGTTTTTACCTACCCAGGAAAAGCCAACCAACCTCGATCT
 23 AY297028.1_SARS_coronavirus_ZJ01_complete_genome                                      95.3%  95.3%     ------------------------------------------------CCTACCCAGGAAAAGCCAACCAACCTCGATCT
 24 AY304486.1_SARS_coronavirus_SZ3_complete_genome                                       95.0%  95.0%     -------------------------------------------------CTACCCAGGAAAAGCCAACCAACCTCGATCT
 25 AY304488.1_SARS_coronavirus_SZ16_complete_genome                                      95.0%  95.0%     -------------------------------------------------CTACCCAGGAAAAGCCAACCAACCTCGATCT
 26 AY304495.1_SARS_coronavirus_GZ50_complete_genome                                      95.0%  95.0%     -------------------------------------------------CTACCCAGGAAAAGCCAACCAACCTCGATCT
 27 AY310120.1_SARS_coronavirus_FRA_complete_genome                                      100.0% 100.0%     ----------------------------------ATATTAGGTTTTTACCTACCCAGGAAAAGCCAACCAACCTCGATCT
 28 AY313906.1_SARS_coronavirus_GD69_complete_genome                                     100.0% 100.0%     ----------------------------------ATATTAGGTTTTTACCTACCCAGGAAAAGCCAACCAACCTCGATCT
 29 AY321118.1_SARS_coronavirus_TWC_complete_genome                                      100.0% 100.0%     ----------------------------------ATATTAGGTTTTTACCTACCCAGGAAAAGCCAACCAACCTCGATCT
 30 AY323977.2_SARS_coronavirus_HSR_1_complete_genome                                    100.0% 100.0%     ----------------------------------ATATTAGGTTTTTACCTACCCAGGAAAAGCCAACCAACCTCGATCT
 31 AY338174.1_SARS_coronavirus_Taiwan_TC1_complete_genome                                77.0%  77.0%     --------------------------------------------------------------------------------
 32 AY338175.1_SARS_coronavirus_Taiwan_TC2_complete_genome                                77.0%  77.0%     --------------------------------------------------------------------------------
 33 AY345986.1_SARS_coronavirus_CUHK-AG01_complete_genome                                 95.0%  95.0%     -------------------------------------------------CTACCCAGGAAAAGCCAACCAACCTCGATCT
 34 AY345987.1_SARS_coronavirus_CUHK-AG02_complete_genome                                 95.0%  95.0%     -------------------------------------------------CTACCCAGGAAAAGCCAACCAACCTCGATCT
 35 AY345988.1_SARS_coronavirus_CUHK-AG03_complete_genome                                 95.0%  95.0%     -------------------------------------------------CTACCCAGGAAAAGCCAACCAACCTCGATCT
 36 AY348314.1_SARS_coronavirus_Taiwan_TC3_complete_genome                                77.0%  77.0%     --------------------------------------------------------------------------------
 37 AY350750.1_SARS_coronavirus_PUMC01_complete_genome                                    95.7%  95.7%     -----------------------------------------------ACCTACCCAGGAAAAGCCAACCAACCTCGATCT
 38 AY351680.1_SARS_coronavirus_ZMY_1_complete_genome                                    100.0%  99.7%     ----------------------------------TTATTAGGTTTTTACCTACCCAGGAAAAGCCAACCAACCTCGATCT
 39 AY357075.1_SARS_coronavirus_PUMC02_complete_genome                                    95.3%  95.3%     ------------------------------------------------CCTACCCAGGAAAAGCCAACCAACCTCGATCT
 40 AY357076.1_SARS_coronavirus_PUMC03_complete_genome                                    95.3%  95.3%     ------------------------------------------------CCTACCCAGGAAAAGCCAACCAACCTCGATCT
 41 AY362698.1_SARS_coronavirus_TWC2_complete_genome                                     100.0% 100.0%     ----------------------------------ATATTAGGTTTTTACCTACCCAGGAAAAGCCAACCAACCTCGATCT
 42 AY362699.1_SARS_coronavirus_TWC3_complete_genome                                     100.0% 100.0%     ----------------------------------ATATTAGGTTTTTACCTACCCAGGAAAAGCCAACCAACCTCGATCT
 43 AY390556.1_SARS_coronavirus_GZ02_complete_genome                                     100.0% 100.0%     ----------------------------------ATATTAGGTTTTTACCTACCCAGGAAAAGCCAACCAACCTCGATCT
 44 AY394850.2_SARS_coronavirus_WHU_complete_genome                                      100.0% 100.0%     ----------------------------------ATATTAGGTTTTTACCTACCCAGGAAAAGCCAACCAACCTCGATCT
 45 AY394977.1_SARS_coronavirus_GZ-A_partial_genome                                       83.0%  83.0%     --------------------------------------------------------------------------------
 46 AY394978.1_SARS_coronavirus_GZ-B_complete_genome                                      76.0%  76.0%     --------------------------------------------------------------------------------
 47 AY394979.1_SARS_coronavirus_GZ-C_complete_genome                                      82.7%  82.7%     --------------------------------------------------------------------------------
 48 AY394980.1_SARS_coronavirus_GZ-D_partial_genome                                       84.7%  84.3%     --------------------------------------------------------------------------------
 49 AY394981.1_SARS_coronavirus_HGZ8L1-A_partial_genome                                   83.0%  83.0%     --------------------------------------------------------------------------------
 50 AY394982.1_SARS_coronavirus_HGZ8L1-B_partial_genome                                   82.7%  82.7%     --------------------------------------------------------------------------------
 51 AY394983.1_SARS_coronavirus_HSZ2-A_complete_genome                                    82.7%  82.7%     --------------------------------------------------------------------------------
 52 AY394984.1_SARS_coronavirus_HSZ-A_partial_genome                                      84.3%  84.3%     --------------------------------------------------------------------------------
 53 AY394985.1_SARS_coronavirus_HSZ-Bb_complete_genome                                    16.7%  16.7%     --------------------------------------------------------------------------------
 54 AY394986.1_SARS_coronavirus_HSZ-Cb_complete_genome                                    83.0%  83.0%     --------------------------------------------------------------------------------
 55 AY394987.1_SARS_coronavirus_HZS2-Fb_complete_genome                                   86.0%  86.0%     ----------------------------------------------------------------------------ATCT
 56 AY394988.1_SARS_coronavirus_JMD_partial_genome                                        71.3%  71.0%     --------------------------------------------------------------------------------
 57 AY394989.1_SARS_coronavirus_HZS2-D_complete_genome                                    95.0%  95.0%     -------------------------------------------------CTACCCAGGAAAAGCCAACCAACCTCGATCT
 58 AY394990.1_SARS_coronavirus_HZS2-E_complete_genome                                    95.0%  95.0%     -------------------------------------------------CTACCCAGGAAAAGCCAACCAACCTCGATCT
 59 AY394991.1_SARS_coronavirus_HZS2-Fc_complete_genome                                   95.0%  95.0%     -------------------------------------------------CTACCCAGGAAAAGCCAACCAACCTCGATCT
 60 AY394992.1_SARS_coronavirus_HZS2-C_complete_genome                                    95.0%  95.0%     -------------------------------------------------CTACCCAGGAAAAGCCAACCAACCTCGATCT
 61 AY394993.1_SARS_coronavirus_HGZ8L2_complete_genome                                    95.0%  95.0%     -------------------------------------------------CTACCCAGGAAAAGCCAACCAACCTCGATCT
 62 AY394994.1_SARS_coronavirus_HSZ-Bc_complete_genome                                    95.0%  95.0%     -------------------------------------------------CTACCCAGGAAAAGCCAACCAACCTCGATCT
 63 AY394995.1_SARS_coronavirus_HSZ-Cc_complete_genome                                    95.0%  95.0%     -------------------------------------------------CTACCCAGGAAAAGCCAACCAACCTCGATCT
 64 AY394996.1_SARS_coronavirus_ZS-B_complete_genome                                      95.0%  95.0%     -------------------------------------------------CTACCCAGGAAAAGCCAACCAACCTCGATCT
 65 AY394997.1_SARS_coronavirus_ZS-A_complete_genome                                      95.0%  95.0%     -------------------------------------------------CTACCCAGGAAAAGCCAACCAACCTCGATCT
 66 AY394998.1_SARS_coronavirus_LC1_complete_genome                                       95.0%  95.0%     -------------------------------------------------CTACCCAGGAAAAGCCAACCAACCTCGATCT
 67 AY394999.1_SARS_coronavirus_LC2_complete_genome                                       95.0%  95.0%     -------------------------------------------------CTACCCAGGAAAAGCCAACCAACCTCGATCT
 68 AY395000.1_SARS_coronavirus_LC3_complete_genome                                       95.0%  95.0%     -------------------------------------------------CTACCCAGGAAAAGCCAACCAACCTCGATCT
 69 AY395001.1_SARS_coronavirus_LC4_complete_genome                                       95.0%  95.0%     -------------------------------------------------CTACCCAGGAAAAGCCAACCAACCTCGATCT
 70 AY395002.1_SARS_coronavirus_LC5_complete_genome                                       95.0%  95.0%     -------------------------------------------------CTACCCAGGAAAAGCCAACCAACCTCGATCT
 71 AY395003.1_SARS_coronavirus_ZS-C_complete_genome                                      83.0%  83.0%     --------------------------------------------------------------------------------
 72 AY395004.1_SARS_coronavirus_HZS2-Bb_partial_genome                                    84.3%  84.3%     --------------------------------------------------------------------------------
 73 AY427439.1_SARS_coronavirus_AS_complete_genome                                        94.7%  94.7%     --------------------------------------------------TACCCAGGAAAAGCCAACCAACCTCGATCT
 74 AY461660.1_SARS_coronavirus_SoD_complete_genome                                       95.0%  94.7%     -------------------------------------------------CTACCCAGGAAAAGCCAACGAACCTCGATCT
 75 AY463059.1_SARS_coronavirus_ShanghaiQXC1_complete_genome                              73.7%  73.7%     --------------------------------------------------------------------------------
 76 AY463060.1_SARS_coronavirus_ShanghaiQXC2_complete_genome                              73.7%  73.7%     --------------------------------------------------------------------------------
 77 AY485277.1_SARS_coronavirus_Sino1-11_complete_genome                                 100.0% 100.0%     ----------------------------------ATATTAGGTTTTTACCTACCCAGGAAAAGCCAACCAACCTCGATCT
 78 AY485278.1_SARS_coronavirus_Sino3-11_complete_genome                                 100.0% 100.0%     ----------------------------------ATATTAGGTTTTTACCTACCCAGGAAAAGCCAACCAACCTCGATCT
 79 AY502923.1_SARS_coronavirus_TW10_complete_genome                                     100.0% 100.0%     ----------------------------------ATATTAGGTTTTTACCTACCCAGGAAAAGCCAACCAACCTCGATCT
 80 AY502924.1_SARS_coronavirus_TW11_complete_genome                                     100.0% 100.0%     ----------------------------------ATATTAGGTTTTTACCTACCCAGGAAAAGCCAACCAACCTCGATCT
 81 AY502925.1_SARS_coronavirus_TW2_complete_genome                                      100.0% 100.0%     ----------------------------------ATATTAGGTTTTTACCTACCCAGGAAAAGCCAACCAACCTCGATCT
 82 AY502926.1_SARS_coronavirus_TW3_complete_genome                                      100.0% 100.0%     ----------------------------------ATATTAGGTTTTTACCTACCCAGGAAAAGCCAACCAACCTCGATCT
 83 AY502927.1_SARS_coronavirus_TW4_complete_genome                                      100.0% 100.0%     ----------------------------------ATATTAGGTTTTTACCTACCCAGGAAAAGCCAACCAACCTCGATCT
 84 AY502928.1_SARS_coronavirus_TW5_complete_genome                                      100.0% 100.0%     ----------------------------------ATATTAGGTTTTTACCTACCCAGGAAAAGCCAACCAACCTCGATCT
 85 AY502929.1_SARS_coronavirus_TW6_complete_genome                                      100.0% 100.0%     ----------------------------------ATATTAGGTTTTTACCTACCCAGGAAAAGCCAACCAACCTCGATCT
 86 AY502930.1_SARS_coronavirus_TW7_complete_genome                                      100.0% 100.0%     ----------------------------------ATATTAGGTTTTTACCTACCCAGGAAAAGCCAACCAACCTCGATCT
 87 AY502931.1_SARS_coronavirus_TW8_complete_genome                                      100.0% 100.0%     ----------------------------------ATATTAGGTTTTTACCTACCCAGGAAAAGCCAACCAACCTCGATCT
 88 AY502932.1_SARS_coronavirus_TW9_complete_genome                                      100.0% 100.0%     ----------------------------------ATATTAGGTTTTTACCTACCCAGGAAAAGCCAACCAACCTCGATCT
 89 AY508724.1_SARS_coronavirus_NS-1_complete_genome                                      94.7%  94.7%     --------------------------------------------------TACCCAGGAAAAGCCAACCAACCTCGATCT
 90 AY559081.1_SARS_coronavirus_Sin842_complete_genome                                    95.7%  95.3%     -----------------------------------------------ACGTACCCAGGAAAAGCCAACCAACCTCGATCT
 91 AY559082.1_SARS_coronavirus_Sin852_complete_genome                                    99.7%  96.3%     -----------------------------------AAATTTTGTTTCATCTATACAGGAAAAGCCAACCAACCTCGATCT
 92 AY559083.1_SARS_coronavirus_Sin3408_complete_genome                                   88.7%  85.3%     AAATTTTGTTTCATCTATACAGGAAAAGCCAACCAACCTCGATCTCTTGTTACCCAGGAAAAGCCAACCAACCTCGATCT
 93 AY559084.1_SARS_coronavirus_Sin3765V_complete_genome                                  94.7%  94.7%     --------------------------------------------------TACCCAGGAAAAGCCAACCAACCTCGATCT
 94 AY559085.1_SARS_coronavirus_Sin848_complete_genome                                    94.7%  94.7%     --------------------------------------------------TACCCAGGAAAAGCCAACCAACCTCGATCT
 95 AY559086.1_SARS_coronavirus_Sin849_complete_genome                                    94.7%  94.7%     --------------------------------------------------TACCCAGGAAAAGCCAACCAACCTCGATCT
 96 AY559087.1_SARS_coronavirus_Sin3725V_complete_genome                                  94.7%  94.7%     --------------------------------------------------TACCCAGGAAAAGCCAACCAACCTCGATCT
 97 AY559088.1_SARS_coronavirus_SinP1_complete_genome                                     94.7%  94.7%     --------------------------------------------------TACCCAGGAAAAGCCAACCAACCTCGATCT
 98 AY559089.1_SARS_coronavirus_SinP2_complete_genome                                     94.7%  94.7%     --------------------------------------------------TACCCAGGAAAAGCCAACCAACCTCGATCT
 99 AY559090.1_SARS_coronavirus_SinP3_complete_genome                                     95.3%  92.7%     ------------------------------------------------TACCCAGGGGGAAAGCCAACCAACCTCGATCT
100 AY559091.1_SARS_coronavirus_SinP4_complete_genome                                     94.7%  94.7%     --------------------------------------------------TACCCAGGAAAAGCCAACCAACCTCGATCT
101 AY559092.1_SARS_coronavirus_SinP5_complete_genome                                     94.7%  94.7%     --------------------------------------------------TACCCAGGAAAAGCCAACCAACCTCGATCT
102 AY559093.1_SARS_coronavirus_Sin845_complete_genome                                    94.7%  94.7%     --------------------------------------------------TACCCAGGAAAAGCCAACCAACCTCGATCT
103 AY559094.1_SARS_coronavirus_Sin846_complete_genome                                    94.7%  94.7%     --------------------------------------------------TACCCAGGAAAAGCCAACCAACCTCGATCT
104 AY559095.1_SARS_coronavirus_Sin847_complete_genome                                    96.7%  96.0%     --------------------------------------------TTTCTCTACCCAGGAAAAGCCAACCAACCTCGATCT
105 AY559096.1_SARS_coronavirus_Sin850_complete_genome                                    97.3%  95.7%     ------------------------------------------ATATTAGGTAACCAGGAAAAGCCAACCAACCTCGATCT
106 AY559097.1_SARS_coronavirus_Sin3408L_complete_genome                                  94.7%  94.7%     --------------------------------------------------TACCCAGGAAAAGCCAACCAACCTCGATCT
107 AY595412.1_SARS_coronavirus_LLJ-2004_complete_genome                                  93.0%  93.0%     -------------------------------------------------------AGGAAAAGCCAACCAACCTCGATCT
108 AY654624.1_SARS_coronavirus_TJF_complete_genome                                       99.7%  99.0%     -----------------------------------TATTAGGTTTTTACCTACCCAGGAAAAGCCAACCGACCTCGATCT
109 AY714217.1_SARS_Coronavirus_CDC*200301157_complete_genome                            100.0% 100.0%     ----------------------------------ATATTAGGTTTTTACCTACCCAGGAAAAGCCAACCAACCTCGATCT
110 CQ903009.1_Sequence_13_from_Patent_WO2004094667                                      100.0%  99.7%     ----------------------------------TTATTAGGTTTTTACCTACCCAGGAAAAGCCAACCAACCTCGATCT
111 CQ918584.1_Sequence_1_from_Patent_WO2004096842                                        95.0%  95.0%     -------------------------------------------------CTACCCAGGAAAAGCCAACCAACCTCGATCT
112 CQ918585.1_Sequence_2_from_Patent_WO2004096842                                        95.0%  95.0%     -------------------------------------------------CTACCCAGGAAAAGCCAACCAACCTCGATCT
113 CQ918598.1_Sequence_15_from_Patent_WO2004096842                                      100.0% 100.0%     ----------------------------------ATATTAGGTTTTTACCTACCCAGGAAAAGCCAACCAACCTCGATCT
114 NC_004718.3_SARS_coronavirus_complete_genome                                         100.0% 100.0%     ----------------------------------ATATTAGGTTTTTACCTACCCAGGAAAAGCCAACCAACCTCGATCT
    consensus/100%                                                                                         ................................................................................
    consensus/90%                                                                                          ................................................................................
    consensus/80%                                                                                          ..................................................TAsCCAGGAAAAGCCAACCAACCTCGATCT
    consensus/70%                                                                                          ..................................................TACCCAGGAAAAGCCAACCAACCTCGATCT

                                                                                            cov    pid  81         .         1         .         .         .         .         :         . 160
  1 AP006557.1_SARS_coronavirus_TWH_genomic_RNA_complete_genome                          100.0% 100.0%     CTTGTAGATCTGTTCTCTAAACGAACTTTAAAATCTGTGTAGCTGTCGCTCGGCTGCATGCCTAGTGCACCTACGCAGTA
  2 AP006558.1_SARS_coronavirus_TWJ_genomic_RNA_complete_genome                          100.0% 100.0%     CTTGTAGATCTGTTCTCTAAACGAACTTTAAAATCTGTGTAGCTGTCGCTCGGCTGCATGCCTAGTGCACCTACGCAGTA
  3 AP006559.1_SARS_coronavirus_TWK_genomic_RNA_complete_genome                          100.0% 100.0%     CTTGTAGATCTGTTCTCTAAACGAACTTTAAAATCTGTGTAGCTGTCGCTCGGCTGCATGCCTAGTGCACCTACGCAGTA
  4 AP006560.1_SARS_coronavirus_TWS_genomic_RNA_complete_genome                          100.0% 100.0%     CTTGTAGATCTGTTCTCTAAACGAACTTTAAAATCTGTGTAGCTGTCGCTCGGCTGCATGCCTAGTGCACCTACGCAGTA
  5 AP006561.1_SARS_coronavirus_TWY_genomic_RNA_complete_genome                          100.0% 100.0%     CTTGTAGATCTGTTCTCTAAACGAACTTTAAAATCTGTGTAGCTGTCGCTCGGCTGCATGCCTAGTGCACCTACGCAGTA
  6 AY274119.3_Severe_acute_respiratory_syndrome-related_coronavirus_isolate_Tor2_complete_genome 100.0% 100.0%     CTTGTAGATCTGTTCTCTAAACGAACTTTAAAATCTGTGTAGCTGTCGCTCGGCTGCATGCCTAGTGCACCTACGCAGTA
  7 AY278487.3_SARS_coronavirus_BJ02_complete_genome                                     100.0% 100.0%     CTTGTAGATCTGTTCTCTAAACGAACTTTAAAATCTGTGTAGCTGTCGCTCGGCTGCATGCCTAGTGCACCTACGCAGTA
  8 AY278488.2_SARS_coronavirus_BJ01_complete_genome                                      93.7%  93.7%     CTTGTAGATCTGTTCTCTAAACGAACTTTAAAATCTGTGTAGCTGTCGCTCGGCTGCATGCCTAGTGCACCTACGCAGTA
  9 AY278489.2_SARS_coronavirus_GD01_complete_genome                                      94.7%  94.7%     CTTGTAGATCTGTTCTCTAAACGAACTTTAAAATCTGTGTAGCTGTCGCTCGGCTGCATGCCTAGTGCACCTACGCAGTA
 10 AY278490.3_SARS_coronavirus_BJ03_complete_genome                                      98.7%  98.7%     CTTGTAGATCTGTTCTCTAAACGAACTTTAAAATCTGTGTAGCTGTCGCTCGGCTGCATGCCTAGTGCACCTACGCAGTA
 11 AY278491.2_SARS_coronavirus_HKU-39849_complete_genome                                100.0% 100.0%     CTTGTAGATCTGTTCTCTAAACGAACTTTAAAATCTGTGTAGCTGTCGCTCGGCTGCATGCCTAGTGCACCTACGCAGTA
 12 AY278554.2_SARS_coronavirus_CUHK-W1_complete_genome                                   95.0%  95.0%     CTTGTAGATCTGTTCTCTAAACGAACTTTAAAATCTGTGTAGCTGTCGCTCGGCTGCATGCCTAGTGCACCTACGCAGTA
 13 AY278741.1_SARS_coronavirus_Urbani_complete_genome                                   100.0% 100.0%     CTTGTAGATCTGTTCTCTAAACGAACTTTAAAATCTGTGTAGCTGTCGCTCGGCTGCATGCCTAGTGCACCTACGCAGTA
 14 AY279354.2_SARS_coronavirus_BJ04_complete_genome                                      94.7%  94.7%     CTTGTAGATCTGTTCTCTAAACGAACTTTAAAATCTGTGTAGCTGTCGCTCGGCTGCATGCCTAGTGCACCTACGCAGTA
 15 AY282752.2_SARS_coronavirus_CUHK-Su10_complete_genome                                 95.0%  95.0%     CTTGTAGATCTGTTCTCTAAACGAACTTTAAAATCTGTGTAGCTGTCGCTCGGCTGCATGCCTAGTGCACCTACGCAGTA
 16 AY283794.1_SARS_coronavirus_Sin2500_complete_genome                                   94.7%  94.7%     CTTGTAGATCTGTTCTCTAAACGAACTTTAAAATCTGTGTAGCTGTCGCTCGGCTGCATGCCTAGTGCACCTACGCAGTA
 17 AY283795.1_SARS_coronavirus_Sin2677_complete_genome                                   94.7%  94.7%     CTTGTAGATCTGTTCTCTAAACGAACTTTAAAATCTGTGTAGCTGTCGCTCGGCTGCATGCCTAGTGCACCTACGCAGTA
 18 AY283796.1_SARS_coronavirus_Sin2679_complete_genome                                   94.7%  94.7%     CTTGTAGATCTGTTCTCTAAACGAACTTTAAAATCTGTGTAGCTGTCGCTCGGCTGCATGCCTAGTGCACCTACGCAGTA
 19 AY283797.1_SARS_coronavirus_Sin2748_complete_genome                                   94.7%  94.7%     CTTGTAGATCTGTTCTCTAAACGAACTTTAAAATCTGTGTAGCTGTCGCTCGGCTGCATGCCTAGTGCACCTACGCAGTA
 20 AY283798.2_SARS_coronavirus_Sin2774_complete_genome                                   94.7%  94.7%     CTTGTAGATCTGTTCTCTAAACGAACTTTAAAATCTGTGTAGCTGTCGCTCGGCTGCATGCCTAGTGCACCTACGCAGTA
 21 AY291315.1_SARS_coronavirus_Frankfurt_1_complete_genome                              100.0% 100.0%     CTTGTAGATCTGTTCTCTAAACGAACTTTAAAATCTGTGTAGCTGTCGCTCGGCTGCATGCCTAGTGCACCTACGCAGTA
 22 AY291451.1_SARS_coronavirus_TW1_complete_genome                                      100.0% 100.0%     CTTGTAGATCTGTTCTCTAAACGAACTTTAAAATCTGTGTAGCTGTCGCTCGGCTGCATGCCTAGTGCACCTACGCAGTA
 23 AY297028.1_SARS_coronavirus_ZJ01_complete_genome                                      95.3%  95.3%     CTTGTAGATCTGTTCTCTAAACGAACTTTAAAATCTGTGTAGCTGTCGCTCGGCTGCATGCCTAGTGCACCTACGCAGTA
 24 AY304486.1_SARS_coronavirus_SZ3_complete_genome                                       95.0%  95.0%     CTTGTAGATCTGTTCTCTAAACGAACTTTAAAATCTGTGTAGCTGTCGCTCGGCTGCATGCCTAGTGCACCTACGCAGTA
 25 AY304488.1_SARS_coronavirus_SZ16_complete_genome                                      95.0%  95.0%     CTTGTAGATCTGTTCTCTAAACGAACTTTAAAATCTGTGTAGCTGTCGCTCGGCTGCATGCCTAGTGCACCTACGCAGTA
 26 AY304495.1_SARS_coronavirus_GZ50_complete_genome                                      95.0%  95.0%     CTTGTAGATCTGTTCTCTAAACGAACTTTAAAATCTGTGTAGCTGTCGCTCGGCTGCATGCCTAGTGCACCTACGCAGTA
 27 AY310120.1_SARS_coronavirus_FRA_complete_genome                                      100.0% 100.0%     CTTGTAGATCTGTTCTCTAAACGAACTTTAAAATCTGTGTAGCTGTCGCTCGGCTGCATGCCTAGTGCACCTACGCAGTA
 28 AY313906.1_SARS_coronavirus_GD69_complete_genome                                     100.0% 100.0%     CTTGTAGATCTGTTCTCTAAACGAACTTTAAAATCTGTGTAGCTGTCGCTCGGCTGCATGCCTAGTGCACCTACGCAGTA
 29 AY321118.1_SARS_coronavirus_TWC_complete_genome                                      100.0% 100.0%     CTTGTAGATCTGTTCTCTAAACGAACTTTAAAATCTGTGTAGCTGTCGCTCGGCTGCATGCCTAGTGCACCTACGCAGTA
 30 AY323977.2_SARS_coronavirus_HSR_1_complete_genome                                    100.0% 100.0%     CTTGTAGATCTGTTCTCTAAACGAACTTTAAAATCTGTGTAGCTGTCGCTCGGCTGCATGCCTAGTGCACCTACGCAGTA
 31 AY338174.1_SARS_coronavirus_Taiwan_TC1_complete_genome                                77.0%  77.0%     -----------------------AACTTTAAAATCTGTGTAGCTGTCGCTCGGCTGCATGCCTAGTGCACCTACGCAGTA
 32 AY338175.1_SARS_coronavirus_Taiwan_TC2_complete_genome                                77.0%  77.0%     -----------------------AACTTTAAAATCTGTGTAGCTGTCGCTCGGCTGCATGCCTAGTGCACCTACGCAGTA
```

```
 33 AY345986.1_SARS_coronavirus_CUHK-AG01_complete_genome                                      95.0%  95.0%    CTTGTAGATCTGTTCTCTAAACGAACTTTAAAATCTGTGTAGCTGTCGCTCGGCTGCATGCCTAGTGCACCTACGCAGTA
 34 AY345987.1_SARS_coronavirus_CUHK-AG02_complete_genome                                      95.0%  95.0%    CTTGTAGATCTGTTCTCTAAACGAACTTTAAAATCTGTGTAGCTGTCGCTCGGCTGCATGCCTAGTGCACCTACGCAGTA
 35 AY345988.1_SARS_coronavirus_CUHK-AG03_complete_genome                                      95.0%  95.0%    CTTGTAGATCTGTTCTCTAAACGAACTTTAAAATCTGTGTAGCTGTCGCTCGGCTGCATGCCTAGTGCACCTACGCAGTA
 36 AY348314.1_SARS_coronavirus_Taiwan_TC3_complete_genome                                     77.0%  77.0%    -----------------------AACTTTAAAATCTGTGTAGCTGTCGCTCGGCTGCATGCCTAGTGCACCTACGCAGTA
 37 AY350750.1_SARS_coronavirus_PUMC01_complete_genome                                         95.7%  95.7%    CTTGTAGATCTGTTCTCTAAACGAACTTTAAAATCTGTGTAGCTGTCGCTCGGCTGCATGCCTAGTGCACCTACGCAGTA
 38 AY351680.1_SARS_coronavirus_ZMY_1_complete_genome                                         100.0%  99.7%    CTTGTAGATCTGTTCTCTAAACGAACTTTAAAATCTGTGTAGCTGTCGCTCGGCTGCATGCCTAGTGCACCTACGCAGTA
 39 AY357075.1_SARS_coronavirus_PUMC02_complete_genome                                         95.3%  95.3%    CTTGTAGATCTGTTCTCTAAACGAACTTTAAAATCTGTGTAGCTGTCGCTCGGCTGCATGCCTAGTGCACCTACGCAGTA
 40 AY357076.1_SARS_coronavirus_PUMC03_complete_genome                                         95.3%  95.3%    CTTGTAGATCTGTTCTCTAAACGAACTTTAAAATCTGTGTAGCTGTCGCTCGGCTGCATGCCTAGTGCACCTACGCAGTA
 41 AY362698.1_SARS_coronavirus_TWC2_complete_genome                                          100.0% 100.0%    CTTGTAGATCTGTTCTCTAAACGAACTTTAAAATCTGTGTAGCTGTCGCTCGGCTGCATGCCTAGTGCACCTACGCAGTA
 42 AY362699.1_SARS_coronavirus_TWC3_complete_genome                                          100.0% 100.0%    CTTGTAGATCTGTTCTCTAAACGAACTTTAAAATCTGTGTAGCTGTCGCTCGGCTGCATGCCTAGTGCACCTACGCAGTA
 43 AY390556.1_SARS_coronavirus_GZ02_complete_genome                                          100.0% 100.0%    CTTGTAGATCTGTTCTCTAAACGAACTTTAAAATCTGTGTAGCTGTCGCTCGGCTGCATGCCTAGTGCACCTACGCAGTA
 44 AY394850.2_SARS_coronavirus_WHU_complete_genome                                           100.0% 100.0%    CTTGTAGATCTGTTCTCTAAACGAACTTTAAAATCTGTGTAGCTGTCGCTCGGCTGCATGCCTAGTGCACCTACGCAGTA
 45 AY394977.1_SARS_coronavirus_GZ-A_partial_genome                                            83.0%  83.0%    -----AGATCTGTTCTCTAAACGAACTTTAAAATCTGTGTAGCTGTCGCTCGGCTGCATGCCTAGTGCACCTACGCAGTA
 46 AY394978.1_SARS_coronavirus_GZ-B_complete_genome                                           76.0%  76.0%    --------------------------TTTAAAATCTGTGTAGCTGTCGCTCGGCTGCATGCCTAGTGCACCTACGCAGTA
 47 AY394979.1_SARS_coronavirus_GZ-C_complete_genome                                           82.7%  82.7%    ------GATCTGTTCTCTAAACGAACTTTAAAATCTGTGTAGCTGTCGCTCGGCTGCATGCCTAGTGCACCTACGCAGTA
 48 AY394980.1_SARS_coronavirus_GZ-D_partial_genome                                            84.7%  84.3%    CTTGTAGATCTGTTCTCTAAACGAACTTTAAAATCTGTGTAGCTGTCGCTCGGCTGCATGCCTAGTGCACCTACGCAGTA
 49 AY394981.1_SARS_coronavirus_HGZ8L1-A_partial_genome                                        83.0%  83.0%    -----AGATCTGTTCTCTAAACGAACTTTAAAATCTGTGTAGCTGTCGCTCGGCTGCATGCCTAGTGCACCTACGCAGTA
 50 AY394982.1_SARS_coronavirus_HGZ8L1-B_partial_genome                                        82.7%  82.7%    ------GATCTGTTCTCTAAACGAACTTTAAAATCTGTGTAGCTGTCGCTCGGCTGCATGCCTAGTGCACCTACGCAGTA
 51 AY394983.1_SARS_coronavirus_HSZ2-A_complete_genome                                         82.7%  82.7%    ------GATCTGTTCTCTAAACGAACTTTAAAATCTGTGTAGCTGTCGCTCGGCTGCATGCCTAGTGCACCTACGCAGTA
 52 AY394984.1_SARS_coronavirus_HSZ-A_partial_genome                                           84.3%  84.3%    -TTGTAGATCTGTTCTCTAAACGAACTTTAAAATCTGTGTAGCTGTCGCTCGGCTGCATGCCTAGTGCACCTACGCAGTA
 53 AY394985.1_SARS_coronavirus_HSZ-Bb_complete_genome                                         16.7%  16.7%    --------------------------------------------------------------------------------
 54 AY394986.1_SARS_coronavirus_HSZ-Cb_complete_genome                                         83.0%  83.0%    -----AGATCTGTTCTCTAAACGAACTTTAAAATCTGTGTAGCTGTCGCTCGGCTGCATGCCTAGTGCACCTACGCAGTA
 55 AY394987.1_SARS_coronavirus_HZS2-Fb_complete_genome                                        86.0%  86.0%    CTTGTAGATCTGTTCTCTAAACGAACTTTAAAATCTGTGTAGCTGTCGCTCGGCTGCATGCCTAGTGCACCTACGCAGTA
 56 AY394988.1_SARS_coronavirus_JMD_partial_genome                                             71.3%  71.0%    ----------------------------------------AACTGTCGCTCGGCTGCATGCCTAGTGCACCTACGCAGTA
 57 AY394989.1_SARS_coronavirus_HZS2-D_complete_genome                                         95.0%  95.0%    CTTGTAGATCTGTTCTCTAAACGAACTTTAAAATCTGTGTAGCTGTCGCTCGGCTGCATGCCTAGTGCACCTACGCAGTA
 58 AY394990.1_SARS_coronavirus_HZS2-E_complete_genome                                         95.0%  95.0%    CTTGTAGATCTGTTCTCTAAACGAACTTTAAAATCTGTGTAGCTGTCGCTCGGCTGCATGCCTAGTGCACCTACGCAGTA
 59 AY394991.1_SARS_coronavirus_HZS2-Fc_complete_genome                                        95.0%  95.0%    CTTGTAGATCTGTTCTCTAAACGAACTTTAAAATCTGTGTAGCTGTCGCTCGGCTGCATGCCTAGTGCACCTACGCAGTA
 60 AY394992.1_SARS_coronavirus_HZS2-C_complete_genome                                         95.0%  95.0%    CTTGTAGATCTGTTCTCTAAACGAACTTTAAAATCTGTGTAGCTGTCGCTCGGCTGCATGCCTAGTGCACCTACGCAGTA
 61 AY394993.1_SARS_coronavirus_HGZ8L2_complete_genome                                         95.0%  95.0%    CTTGTAGATCTGTTCTCTAAACGAACTTTAAAATCTGTGTAGCTGTCGCTCGGCTGCATGCCTAGTGCACCTACGCAGTA
 62 AY394994.1_SARS_coronavirus_HSZ-Bc_complete_genome                                         95.0%  95.0%    CTTGTAGATCTGTTCTCTAAACGAACTTTAAAATCTGTGTAGCTGTCGCTCGGCTGCATGCCTAGTGCACCTACGCAGTA
 63 AY394995.1_SARS_coronavirus_HSZ-Cc_complete_genome                                         95.0%  95.0%    CTTGTAGATCTGTTCTCTAAACGAACTTTAAAATCTGTGTAGCTGTCGCTCGGCTGCATGCCTAGTGCACCTACGCAGTA
 64 AY394996.1_SARS_coronavirus_ZS-B_complete_genome                                           95.0%  95.0%    CTTGTAGATCTGTTCTCTAAACGAACTTTAAAATCTGTGTAGCTGTCGCTCGGCTGCATGCCTAGTGCACCTACGCAGTA
 65 AY394997.1_SARS_coronavirus_ZS-A_complete_genome                                           95.0%  95.0%    CTTGTAGATCTGTTCTCTAAACGAACTTTAAAATCTGTGTAGCTGTCGCTCGGCTGCATGCCTAGTGCACCTACGCAGTA
 66 AY394998.1_SARS_coronavirus_LC1_complete_genome                                            95.0%  95.0%    CTTGTAGATCTGTTCTCTAAACGAACTTTAAAATCTGTGTAGCTGTCGCTCGGCTGCATGCCTAGTGCACCTACGCAGTA
 67 AY394999.1_SARS_coronavirus_LC2_complete_genome                                            95.0%  95.0%    CTTGTAGATCTGTTCTCTAAACGAACTTTAAAATCTGTGTAGCTGTCGCTCGGCTGCATGCCTAGTGCACCTACGCAGTA
 68 AY395000.1_SARS_coronavirus_LC3_complete_genome                                            95.0%  95.0%    CTTGTAGATCTGTTCTCTAAACGAACTTTAAAATCTGTGTAGCTGTCGCTCGGCTGCATGCCTAGTGCACCTACGCAGTA
 69 AY395001.1_SARS_coronavirus_LC4_complete_genome                                            95.0%  95.0%    CTTGTAGATCTGTTCTCTAAACGAACTTTAAAATCTGTGTAGCTGTCGCTCGGCTGCATGCCTAGTGCACCTACGCAGTA
 70 AY395002.1_SARS_coronavirus_LC5_complete_genome                                            95.0%  95.0%    CTTGTAGATCTGTTCTCTAAACGAACTTTAAAATCTGTGTAGCTGTCGCTCGGCTGCATGCCTAGTGCACCTACGCAGTA
 71 AY395003.1_SARS_coronavirus_ZS-C_complete_genome                                           83.0%  83.0%    -----AGATCTGTTCTCTAAACGAACTTTAAAATCTGTGTAGCTGTCGCTCGGCTGCATGCCTAGTGCACCTACGCAGTA
 72 AY395004.1_SARS_coronavirus_HZS2-Bb_partial_genome                                         84.3%  84.3%    -TTGTAGATCTGTTCTCTAAACGAACTTTAAAATCTGTGTAGCTGTCGCTCGGCTGCATGCCTAGTGCACCTACGCAGTA
 73 AY427439.1_SARS_coronavirus_AS_complete_genome                                             94.7%  94.7%    CTTGTAGATCTGTTCTCTAAACGAACTTTAAAATCTGTGTAGCTGTCGCTCGGCTGCATGCCTAGTGCACCTACGCAGTA
 74 AY461660.1_SARS_coronavirus_SoD_complete_genome                                            95.0%  94.7%    CTTGTAGATCTGTTCTCTAAACGAACTTTAAAATCTGTGTAGCTGTCGCTCGGCTGCATGCCTAGTGCACCTACGCAGTA
 75 AY463059.1_SARS_coronavirus_ShanghaiQXC1_complete_genome                                   73.7%  73.7%    ---------------------------------TCTGTGTAGCTGTCGCTCGGCTGCATGCCTAGTGCACCTACGCAGTA
 76 AY463060.1_SARS_coronavirus_ShanghaiQXC2_complete_genome                                   73.7%  73.7%    ---------------------------------TCTGTGTAGCTGTCGCTCGGCTGCATGCCTAGTGCACCTACGCAGTA
 77 AY485277.1_SARS_coronavirus_Sino1-11_complete_genome                                      100.0% 100.0%    CTTGTAGATCTGTTCTCTAAACGAACTTTAAAATCTGTGTAGCTGTCGCTCGGCTGCATGCCTAGTGCACCTACGCAGTA
 78 AY485278.1_SARS_coronavirus_Sino3-11_complete_genome                                      100.0% 100.0%    CTTGTAGATCTGTTCTCTAAACGAACTTTAAAATCTGTGTAGCTGTCGCTCGGCTGCATGCCTAGTGCACCTACGCAGTA
 79 AY502923.1_SARS_coronavirus_TW10_complete_genome                                          100.0% 100.0%    CTTGTAGATCTGTTCTCTAAACGAACTTTAAAATCTGTGTAGCTGTCGCTCGGCTGCATGCCTAGTGCACCTACGCAGTA
 80 AY502924.1_SARS_coronavirus_TW11_complete_genome                                          100.0% 100.0%    CTTGTAGATCTGTTCTCTAAACGAACTTTAAAATCTGTGTAGCTGTCGCTCGGCTGCATGCCTAGTGCACCTACGCAGTA
 81 AY502925.1_SARS_coronavirus_TW2_complete_genome                                           100.0% 100.0%    CTTGTAGATCTGTTCTCTAAACGAACTTTAAAATCTGTGTAGCTGTCGCTCGGCTGCATGCCTAGTGCACCTACGCAGTA
 82 AY502926.1_SARS_coronavirus_TW3_complete_genome                                           100.0% 100.0%    CTTGTAGATCTGTTCTCTAAACGAACTTTAAAATCTGTGTAGCTGTCGCTCGGCTGCATGCCTAGTGCACCTACGCAGTA
 83 AY502927.1_SARS_coronavirus_TW4_complete_genome                                           100.0% 100.0%    CTTGTAGATCTGTTCTCTAAACGAACTTTAAAATCTGTGTAGCTGTCGCTCGGCTGCATGCCTAGTGCACCTACGCAGTA
 84 AY502928.1_SARS_coronavirus_TW5_complete_genome                                           100.0% 100.0%    CTTGTAGATCTGTTCTCTAAACGAACTTTAAAATCTGTGTAGCTGTCGCTCGGCTGCATGCCTAGTGCACCTACGCAGTA
 85 AY502929.1_SARS_coronavirus_TW6_complete_genome                                           100.0% 100.0%    CTTGTAGATCTGTTCTCTAAACGAACTTTAAAATCTGTGTAGCTGTCGCTCGGCTGCATGCCTAGTGCACCTACGCAGTA
 86 AY502930.1_SARS_coronavirus_TW7_complete_genome                                           100.0% 100.0%    CTTGTAGATCTGTTCTCTAAACGAACTTTAAAATCTGTGTAGCTGTCGCTCGGCTGCATGCCTAGTGCACCTACGCAGTA
 87 AY502931.1_SARS_coronavirus_TW8_complete_genome                                           100.0% 100.0%    CTTGTAGATCTGTTCTCTAAACGAACTTTAAAATCTGTGTAGCTGTCGCTCGGCTGCATGCCTAGTGCACCTACGCAGTA
 88 AY502932.1_SARS_coronavirus_TW9_complete_genome                                           100.0% 100.0%    CTTGTAGATCTGTTCTCTAAACGAACTTTAAAATCTGTGTAGCTGTCGCTCGGCTGCATGCCTAGTGCACCTACGCAGTA
 89 AY508724.1_SARS_coronavirus_NS-1_complete_genome                                           94.7%  94.7%    CTTGTAGATCTGTTCTCTAAACGAACTTTAAAATCTGTGTAGCTGTCGCTCGGCTGCATGCCTAGTGCACCTACGCAGTA
 90 AY559081.1_SARS_coronavirus_Sin842_complete_genome                                         95.7%  95.3%    CTTGTAGATCTGTTCTCTAAACGAACTTTAAAATCTGTGTAGCTGTCGCTCGGCTGCATGCCTAGTGCACCTACGCAGTA
 91 AY559082.1_SARS_coronavirus_Sin852_complete_genome                                         99.7%  96.3%    CTTGTAGATCTGTTCTCTAAACGAACTTTAAAATCTGTGTAGCTGTCGCTCGGCTGCATGCCTAGTGCACCTACGCAGTA
 92 AY559083.1_SARS_coronavirus_Sin3408_complete_genome                                        88.7%  85.3%    CTTGTAGATCTGTTCTCTAAACGAACTTTAAAATCTGTGTAGCTGTCGCTCGGCTGCATGCCTAGTGCACCTACGCAGTA
 93 AY559084.1_SARS_coronavirus_Sin3765V_complete_genome                                       94.7%  94.7%    CTTGTAGATCTGTTCTCTAAACGAACTTTAAAATCTGTGTAGCTGTCGCTCGGCTGCATGCCTAGTGCACCTACGCAGTA
 94 AY559085.1_SARS_coronavirus_Sin848_complete_genome                                         94.7%  94.7%    CTTGTAGATCTGTTCTCTAAACGAACTTTAAAATCTGTGTAGCTGTCGCTCGGCTGCATGCCTAGTGCACCTACGCAGTA
 95 AY559086.1_SARS_coronavirus_Sin849_complete_genome                                         94.7%  94.7%    CTTGTAGATCTGTTCTCTAAACGAACTTTAAAATCTGTGTAGCTGTCGCTCGGCTGCATGCCTAGTGCACCTACGCAGTA
 96 AY559087.1_SARS_coronavirus_Sin3725V_complete_genome                                       94.7%  94.7%    CTTGTAGATCTGTTCTCTAAACGAACTTTAAAATCTGTGTAGCTGTCGCTCGGCTGCATGCCTAGTGCACCTACGCAGTA
 97 AY559088.1_SARS_coronavirus_SinP1_complete_genome                                          94.7%  94.7%    CTTGTAGATCTGTTCTCTAAACGAACTTTAAAATCTGTGTAGCTGTCGCTCGGCTGCATGCCTAGTGCACCTACGCAGTA
 98 AY559089.1_SARS_coronavirus_SinP2_complete_genome                                          94.7%  94.7%    CTTGTAGATCTGTTCTCTAAACGAACTTTAAAATCTGTGTAGCTGTCGCTCGGCTGCATGCCTAGTGCACCTACGCAGTA
 99 AY559090.1_SARS_coronavirus_SinP3_complete_genome                                          95.3%  92.7%    CTTGTAGATCTGTTCTCTAAACGAACTTTAAAATCTGTGTAGCTGTCGCTCGGCTGCATGCCTAGTGCACCTACGCAGTA
100 AY559091.1_SARS_coronavirus_SinP4_complete_genome                                          94.7%  94.7%    CTTGTAGATCTGTTCTCTAAACGAACTTTAAAATCTGTGTAGCTGTCGCTCGGCTGCATGCCTAGTGCACCTACGCAGTA
101 AY559092.1_SARS_coronavirus_SinP5_complete_genome                                          94.7%  94.7%    CTTGTAGATCTGTTCTCTAAACGAACTTTAAAATCTGTGTAGCTGTCGCTCGGCTGCATGCCTAGTGCACCTACGCAGTA
102 AY559093.1_SARS_coronavirus_Sin845_complete_genome                                         94.7%  94.7%    CTTGTAGATCTGTTCTCTAAACGAACTTTAAAATCTGTGTAGCTGTCGCTCGGCTGCATGCCTAGTGCACCTACGCAGTA
103 AY559094.1_SARS_coronavirus_Sin846_complete_genome                                         94.7%  94.7%    CTTGTAGATCTGTTCTCTAAACGAACTTTAAAATCTGTGTAGCTGTCGCTCGGCTGCATGCCTAGTGCACCTACGCAGTA
104 AY559095.1_SARS_coronavirus_Sin847_complete_genome                                         96.7%  96.0%    CTTGTAGATCTGTTCTCTAAACGAACTTTAAAATCTGTGTAGCTGTCGCTCGGCTGCATGCCTAGTGCACCTACGCAGTA
105 AY559096.1_SARS_coronavirus_Sin850_complete_genome                                         97.3%  95.7%    CTTGTAGATCTGTTCTCTAAACGAACTTTAAAATCTGTGTAGCTGTCGCTCGGCTGCATGCCTAGTGCACCTACGCAGTA
106 AY559097.1_SARS_coronavirus_Sin3408L_complete_genome                                       94.7%  94.7%    CTTGTAGATCTGTTCTCTAAACGAACTTTAAAATCTGTGTAGCTGTCGCTCGGCTGCATGCCTAGTGCACCTACGCAGTA
107 AY595412.1_SARS_coronavirus_LLJ-2004_complete_genome                                       93.0%  93.0%    CTTGTAGATCTGTTCTCTAAACGAACTTTAAAATCTGTGTAGCTGTCGCTCGGCTGCATGCCTAGTGCACCTACGCAGTA
108 AY654624.1_SARS_coronavirus_TJF_complete_genome                                            99.7%  99.0%    CTTGTGGATCTGTTCTCTAAACGAACTTTAAAATCTGTGTAGCTGTCGCTCGGCTGCATGCCTAGTGCACCTACGCAGTA
109 AY714217.1_SARS_Coronavirus_CDC*200301157_complete_genome                                 100.0% 100.0%    CTTGTAGATCTGTTCTCTAAACGAACTTTAAAATCTGTGTAGCTGTCGCTCGGCTGCATGCCTAGTGCACCTACGCAGTA
110 CQ903009.1_Sequence_13_from_Patent_WO2004094667                                           100.0%  99.7%    CTTGTAGATCTGTTCTCTAAACGAACTTTAAAATCTGTGTAGCTGTCGCTCGGCTGCATGCCTAGTGCACCTACGCAGTA
111 CQ918584.1_Sequence_1_from_Patent_WO2004096842                                             95.0%  95.0%    CTTGTAGATCTGTTCTCTAAACGAACTTTAAAATCTGTGTAGCTGTCGCTCGGCTGCATGCCTAGTGCACCTACGCAGTA
112 CQ918585.1_Sequence_2_from_Patent_WO2004096842                                             95.0%  95.0%    CTTGTAGATCTGTTCTCTAAACGAACTTTAAAATCTGTGTAGCTGTCGCTCGGCTGCATGCCTAGTGCACCTACGCAGTA
113 CQ918598.1_Sequence_15_from_Patent_WO2004096842                                           100.0% 100.0%    CTTGTAGATCTGTTCTCTAAACGAACTTTAAAATCTGTGTAGCTGTCGCTCGGCTGCATGCCTAGTGCACCTACGCAGTA
114 NC_004718.3_SARS_coronavirus_complete_genome                                              100.0% 100.0%    CTTGTAGATCTGTTCTCTAAACGAACTTTAAAATCTGTGTAGCTGTCGCTCGGCTGCATGCCTAGTGCACCTACGCAGTA
    consensus/100%                                                                                              ................................................................................
    consensus/90%                                                                                               .....uGATCTGTTCTCTAAACGAACTTTAAAATCTGTGTAGCTGTCGCTCGGCTGCATGCCTAGTGCACCTACGCAGTA
    consensus/80%                                                                                               CTTGTAGATCTGTTCTCTAAACGAACTTTAAAATCTGTGTAGCTGTCGCTCGGCTGCATGCCTAGTGCACCTACGCAGTA
    consensus/70%                                                                                               CTTGTAGATCTGTTCTCTAAACGAACTTTAAAATCTGTGTAGCTGTCGCTCGGCTGCATGCCTAGTGCACCTACGCAGTA

                                                                                              cov    pid 161        .         .         .         2         .         .         .         . 240
  1 AP006557.1_SARS_coronavirus_TWH_genomic_RNA_complete_genome                              100.0% 100.0%    TAAACAATAATAAATTTTACTGTCGTTGACAAGAAACGAGTAACTCGTCCCTCTTCTGCAGACTGCTTACGGTTTCGTCC
  2 AP006558.1_SARS_coronavirus_TWJ_genomic_RNA_complete_genome                              100.0% 100.0%    TAAACAATAATAAATTTTACTGTCGTTGACAAGAAACGAGTAACTCGTCCCTCTTCTGCAGACTGCTTACGGTTTCGTCC
  3 AP006559.1_SARS_coronavirus_TWK_genomic_RNA_complete_genome                              100.0% 100.0%    TAAACAATAATAAATTTTACTGTCGTTGACAAGAAACGAGTAACTCGTCCCTCTTCTGCAGACTGCTTACGGTTTCGTCC
  4 AP006560.1_SARS_coronavirus_TWS_genomic_RNA_complete_genome                              100.0% 100.0%    TAAACAATAATAAATTTTACTGTCGTTGACAAGAAACGAGTAACTCGTCCCTCTTCTGCAGACTGCTTACGGTTTCGTCC
  5 AP006561.1_SARS_coronavirus_TWY_genomic_RNA_complete_genome                              100.0% 100.0%    TAAACAATAATAAATTTTACTGTCGTTGACAAGAAACGAGTAACTCGTCCCTCTTCTGCAGACTGCTTACGGTTTCGTCC
  6 AY274119.3_Severe_acute_respiratory_syndrome-related_coronavirus_isolate_Tor2_complete_genome 100.0% 100.0%    TAAACAATAATAAATTTTACTGTCGTTGACAAGAAACGAGTAACTCGTCCCTCTTCTGCAGACTGCTTACGGTTTCGTCC
  7 AY278487.3_SARS_coronavirus_BJ02_complete_genome                                          100.0% 100.0%    TAAACAATAATAAATTTTACTGTCGTTGACAAGAAACGAGTAACTCGTCCCTCTTCTGCAGACTGCTTACGGTTTCGTCC
  8 AY278488.2_SARS_coronavirus_BJ01_complete_genome                                           93.7%  93.7%    TAAACAATAATAAATTTTACTGTCGTTGACAAGAAACGAGTAACTCGTCCCTCTTCTGCAGACTGCTTACGGTTTCGTCC
  9 AY278489.2_SARS_coronavirus_GD01_complete_genome                                           94.7%  94.7%    TAAACAATAATAAATTTTACTGTCGTTGACAAGAAACGAGTAACTCGTCCCTCTTCTGCAGACTGCTTACGGTTTCGTCC
 10 AY278490.3_SARS_coronavirus_BJ03_complete_genome                                           98.7%  98.7%    TAAACAATAATAAATTTTACTGTCGTTGACAAGAAACGAGTAACTCGTCCCTCTTCTGCAGACTGCTTACGGTTTCGTCC
 11 AY278491.2_SARS_coronavirus_HKU-39849_complete_genome                                     100.0% 100.0%    TAAACAATAATAAATTTTACTGTCGTTGACAAGAAACGAGTAACTCGTCCCTCTTCTGCAGACTGCTTACGGTTTCGTCC
 12 AY278554.2_SARS_coronavirus_CUHK-W1_complete_genome                                        95.0%  95.0%    TAAACAATAATAAATTTTACTGTCGTTGACAAGAAACGAGTAACTCGTCCCTCTTCTGCAGACTGCTTACGGTTTCGTCC
 13 AY278741.1_SARS_coronavirus_Urbani_complete_genome                                        100.0% 100.0%    TAAACAATAATAAATTTTACTGTCGTTGACAAGAAACGAGTAACTCGTCCCTCTTCTGCAGACTGCTTACGGTTTCGTCC
 14 AY279354.2_SARS_coronavirus_BJ04_complete_genome                                           94.7%  94.7%    TAAACAATAATAAATTTTACTGTCGTTGACAAGAAACGAGTAACTCGTCCCTCTTCTGCAGACTGCTTACGGTTTCGTCC
 15 AY282752.2_SARS_coronavirus_CUHK-Su10_complete_genome                                      95.0%  95.0%    TAAACAATAATAAATTTTACTGTCGTTGACAAGAAACGAGTAACTCGTCCCTCTTCTGCAGACTGCTTACGGTTTCGTCC
 16 AY283794.1_SARS_coronavirus_Sin2500_complete_genome                                        94.7%  94.7%    TAAACAATAATAAATTTTACTGTCGTTGACAAGAAACGAGTAACTCGTCCCTCTTCTGCAGACTGCTTACGGTTTCGTCC
 17 AY283795.1_SARS_coronavirus_Sin2677_complete_genome                                        94.7%  94.7%    TAAACAATAATAAATTTTACTGTCGTTGACAAGAAACGAGTAACTCGTCCCTCTTCTGCAGACTGCTTACGGTTTCGTCC
 18 AY283796.1_SARS_coronavirus_Sin2679_complete_genome                                        94.7%  94.7%    TAAACAATAATAAATTTTACTGTCGTTGACAAGAAACGAGTAACTCGTCCCTCTTCTGCAGACTGCTTACGGTTTCGTCC
 19 AY283797.1_SARS_coronavirus_Sin2748_complete_genome                                        94.7%  94.7%    TAAACAATAATAAATTTTACTGTCGTTGACAAGAAACGAGTAACTCGTCCCTCTTCTGCAGACTGCTTACGGTTTCGTCC
 20 AY283798.2_SARS_coronavirus_Sin2774_complete_genome                                        94.7%  94.7%    TAAACAATAATAAATTTTACTGTCGTTGACAAGAAACGAGTAACTCGTCCCTCTTCTGCAGACTGCTTACGGTTTCGTCC
 21 AY291315.1_SARS_coronavirus_Frankfurt_1_complete_genome                                   100.0% 100.0%    TAAACAATAATAAATTTTACTGTCGTTGACAAGAAACGAGTAACTCGTCCCTCTTCTGCAGACTGCTTACGGTTTCGTCC
 22 AY291451.1_SARS_coronavirus_TW1_complete_genome                                           100.0% 100.0%    TAAACAATAATAAATTTTACTGTCGTTGACAAGAAACGAGTAACTCGTCCCTCTTCTGCAGACTGCTTACGGTTTCGTCC
 23 AY297028.1_SARS_coronavirus_ZJ01_complete_genome                                           95.3%  95.3%    TAAACAATAATAAATTTTACTGTCGTTGACAAGAAACGAGTAACTCGTCCCTCTTCTGCAGACTGCTTACGGTTTCGTCC
 24 AY304486.1_SARS_coronavirus_SZ3_complete_genome                                            95.0%  95.0%    TAAACAATAATAAATTTTACTGTCGTTGACAAGAAACGAGTAACTCGTCCCTCTTCTGCAGACTGCTTACGGTTTCGTCC
 25 AY304488.1_SARS_coronavirus_SZ16_complete_genome                                           95.0%  95.0%    TAAACAATAATAAATTTTACTGTCGTTGACAAGAAACGAGTAACTCGTCCCTCTTCTGCAGACTGCTTACGGTTTCGTCC
 26 AY304495.1_SARS_coronavirus_GZ50_complete_genome                                           95.0%  95.0%    TAAACAATAATAAATTTTACTGTCGTTGACAAGAAACGAGTAACTCGTCCCTCTTCTGCAGACTGCTTACGGTTTCGTCC
 27 AY310120.1_SARS_coronavirus_FRA_complete_genome                                           100.0% 100.0%    TAAACAATAATAAATTTTACTGTCGTTGACAAGAAACGAGTAACTCGTCCCTCTTCTGCAGACTGCTTACGGTTTCGTCC
 28 AY313906.1_SARS_coronavirus_GD69_complete_genome                                          100.0% 100.0%    TAAACAATAATAAATTTTACTGTCGTTGACAAGAAACGAGTAACTCGTCCCTCTTCTGCAGACTGCTTACGGTTTCGTCC
 29 AY321118.1_SARS_coronavirus_TWC_complete_genome                                           100.0% 100.0%    TAAACAATAATAAATTTTACTGTCGTTGACAAGAAACGAGTAACTCGTCCCTCTTCTGCAGACTGCTTACGGTTTCGTCC
 30 AY323977.2_SARS_coronavirus_HSR_1_complete_genome                                         100.0% 100.0%    TAAACAATAATAAATTTTACTGTCGTTGACAAGAAACGAGTAACTCGTCCCTCTTCTGCAGACTGCTTACGGTTTCGTCC
 31 AY338174.1_SARS_coronavirus_Taiwan_TC1_complete_genome                                     77.0%  77.0%    TAAACAATAATAAATTTTACTGTCGTTGACAAGAAACGAGTAACTCGTCCCTCTTCTGCAGACTGCTTACGGTTTCGTCC
 32 AY338175.1_SARS_coronavirus_Taiwan_TC2_complete_genome                                     77.0%  77.0%    TAAACAATAATAAATTTTACTGTCGTTGACAAGAAACGAGTAACTCGTCCCTCTTCTGCAGACTGCTTACGGTTTCGTCC
 33 AY345986.1_SARS_coronavirus_CUHK-AG01_complete_genome                                      95.0%  95.0%    TAAACAATAATAAATTTTACTGTCGTTGACAAGAAACGAGTAACTCGTCCCTCTTCTGCAGACTGCTTACGGTTTCGTCC
 34 AY345987.1_SARS_coronavirus_CUHK-AG02_complete_genome                                      95.0%  95.0%    TAAACAATAATAAATTTTACTGTCGTTGACAAGAAACGAGTAACTCGTCCCTCTTCTGCAGACTGCTTACGGTTTCGTCC
 35 AY345988.1_SARS_coronavirus_CUHK-AG03_complete_genome                                      95.0%  95.0%    TAAACAATAATAAATTTTACTGTCGTTGACAAGAAACGAGTAACTCGTCCCTCTTCTGCAGACTGCTTACGGTTTCGTCC
 36 AY348314.1_SARS_coronavirus_Taiwan_TC3_complete_genome                                     77.0%  77.0%    TAAACAATAATAAATTTTACTGTCGTTGACAAGAAACGAGTAACTCGTCCCTCTTCTGCAGACTGCTTACGGTTTCGTCC
 37 AY350750.1_SARS_coronavirus_PUMC01_complete_genome                                         95.7%  95.7%    TAAACAATAATAAATTTTACTGTCGTTGACAAGAAACGAGTAACTCGTCCCTCTTCTGCAGACTGCTTACGGTTTCGTCC
 38 AY351680.1_SARS_coronavirus_ZMY_1_complete_genome                                         100.0%  99.7%    TAAACAATAATAAATTTTACTGTCGTTGACAAGAAACGAGTAACTCGTCCCTCTTCTGCAGACTGCTTACGGTTTCGTCC
 39 AY357075.1_SARS_coronavirus_PUMC02_complete_genome                                         95.3%  95.3%    TAAACAATAATAAATTTTACTGTCGTTGACAAGAAACGAGTAACTCGTCCCTCTTCTGCAGACTGCTTACGGTTTCGTCC
 40 AY357076.1_SARS_coronavirus_PUMC03_complete_genome                                         95.3%  95.3%    TAAACAATAATAAATTTTACTGTCGTTGACAAGAAACGAGTAACTCGTCCCTCTTCTGCAGACTGCTTACGGTTTCGTCC
 41 AY362698.1_SARS_coronavirus_TWC2_complete_genome                                          100.0% 100.0%    TAAACAATAATAAATTTTACTGTCGTTGACAAGAAACGAGTAACTCGTCCCTCTTCTGCAGACTGCTTACGGTTTCGTCC
 42 AY362699.1_SARS_coronavirus_TWC3_complete_genome                                          100.0% 100.0%    TAAACAATAATAAATTTTACTGTCGTTGACAAGAAACGAGTAACTCGTCCCTCTTCTGCAGACTGCTTACGGTTTCGTCC
 43 AY390556.1_SARS_coronavirus_GZ02_complete_genome                                          100.0% 100.0%    TAAACAATAATAAATTTTACTGTCGTTGACAAGAAACGAGTAACTCGTCCCTCTTCTGCAGACTGCTTACGGTTTCGTCC
 44 AY394850.2_SARS_coronavirus_WHU_complete_genome                                           100.0% 100.0%    TAAACAATAATAAATTTTACTGTCGTTGACAAGAAACGAGTAACTCGTCCCTCTTCTGCAGACTGCTTACGGTTTCGTCC
 45 AY394977.1_SARS_coronavirus_GZ-A_partial_genome                                            83.0%  83.0%    TAAACAATAATAAATTTTACTGTCGTTGACAAGAAACGAGTAACTCGTCCCTCTTCTGCAGACTGCTTACGGTTTCGTCC
 46 AY394978.1_SARS_coronavirus_GZ-B_complete_genome                                           76.0%  76.0%    TAAACAATAATAAATTTTACTGTCGTTGACAAGAAACGAGTAACTCGTCCCTCTTCTGCAGACTGCTTACGGTTTCGTCC
 47 AY394979.1_SARS_coronavirus_GZ-C_complete_genome                                           82.7%  82.7%    TAAACAATAATAAATTTTACTGTCGTTGACAAGAAACGAGTAACTCGTCCCTCTTCTGCAGACTGCTTACGGTTTCGTCC
 48 AY394980.1_SARS_coronavirus_GZ-D_partial_genome                                            84.7%  84.3%    TAAACAATAATAAATTTTACTGTCGTTGACAAGAAACGAGTAACTCGTCCCTCTTTTGCAGACTGCTTACGGTTTCGTCC
 49 AY394981.1_SARS_coronavirus_HGZ8L1-A_partial_genome                                        83.0%  83.0%    TAAACAATAATAAATTTTACTGTCGTTGACAAGAAACGAGTAACTCGTCCCTCTTCTGCAGACTGCTTACGGTTTCGTCC
 50 AY394982.1_SARS_coronavirus_HGZ8L1-B_partial_genome                                        82.7%  82.7%    TAAACAATAATAAATTTTACTGTCGTTGACAAGAAACGAGTAACTCGTCCCTCTTCTGCAGACTGCTTACGGTTTCGTCC
 51 AY394983.1_SARS_coronavirus_HSZ2-A_complete_genome                                         82.7%  82.7%    TAAACAATAATAAATTTTACTGTCGTTGACAAGAAACGAGTAACTCGTCCCTCTTCTGCAGACTGCTTACGGTTTCGTCC
 52 AY394984.1_SARS_coronavirus_HSZ-A_partial_genome                                           84.3%  84.3%    TAAACAATAATAAATTTTACTGTCGTTGACAAGAAACGAGTAACTCGTCCCTCTTCTGCAGACTGCTTACGGTTTCGTCC
 53 AY394985.1_SARS_coronavirus_HSZ-Bb_complete_genome                                         16.7%  16.7%    --------------------------------------------------------------------------------
 54 AY394986.1_SARS_coronavirus_HSZ-Cb_complete_genome                                         83.0%  83.0%    TAAACAATAATAAATTTTACTGTCGTTGACAAGAAACGAGTAACTCGTCCCTCTTCTGCAGACTGCTTACGGTTTCGTCC
 55 AY394987.1_SARS_coronavirus_HZS2-Fb_complete_genome                                        86.0%  86.0%    TAAACAATAATAAATTTTACTGTCGTTGACAAGAAACGAGTAACTCGTCCCTCTTCTGCAGACTGCTTACGGTTTCGTCC
 56 AY394988.1_SARS_coronavirus_JMD_partial_genome                                             71.3%  71.0%    TAAACAATAATAAATTTTACTGTCGTTGACAAGAAACGAGTAACTCGTCCCTCTTCTGCAGACTGCTTACGGTTTCGTCC
 57 AY394989.1_SARS_coronavirus_HZS2-D_complete_genome                                         95.0%  95.0%    TAAACAATAATAAATTTTACTGTCGTTGACAAGAAACGAGTAACTCGTCCCTCTTCTGCAGACTGCTTACGGTTTCGTCC
 58 AY394990.1_SARS_coronavirus_HZS2-E_complete_genome                                         95.0%  95.0%    TAAACAATAATAAATTTTACTGTCGTTGACAAGAAACGAGTAACTCGTCCCTCTTCTGCAGACTGCTTACGGTTTCGTCC
 59 AY394991.1_SARS_coronavirus_HZS2-Fc_complete_genome                                        95.0%  95.0%    TAAACAATAATAAATTTTACTGTCGTTGACAAGAAACGAGTAACTCGTCCCTCTTCTGCAGACTGCTTACGGTTTCGTCC
 60 AY394992.1_SARS_coronavirus_HZS2-C_complete_genome                                         95.0%  95.0%    TAAACAATAATAAATTTTACTGTCGTTGACAAGAAACGAGTAACTCGTCCCTCTTCTGCAGACTGCTTACGGTTTCGTCC
 61 AY394993.1_SARS_coronavirus_HGZ8L2_complete_genome                                         95.0%  95.0%    TAAACAATAATAAATTTTACTGTCGTTGACAAGAAACGAGTAACTCGTCCCTCTTCTGCAGACTGCTTACGGTTTCGTCC
 62 AY394994.1_SARS_coronavirus_HSZ-Bc_complete_genome                                         95.0%  95.0%    TAAACAATAATAAATTTTACTGTCGTTGACAAGAAACGAGTAACTCGTCCCTCTTCTGCAGACTGCTTACGGTTTCGTCC
 63 AY394995.1_SARS_coronavirus_HSZ-Cc_complete_genome                                         95.0%  95.0%    TAAACAATAATAAATTTTACTGTCGTTGACAAGAAACGAGTAACTCGTCCCTCTTCTGCAGACTGCTTACGGTTTCGTCC
 64 AY394996.1_SARS_coronavirus_ZS-B_complete_genome                                           95.0%  95.0%    TAAACAATAATAAATTTTACTGTCGTTGACAAGAAACGAGTAACTCGTCCCTCTTCTGCAGACTGCTTACGGTTTCGTCC
 65 AY394997.1_SARS_coronavirus_ZS-A_complete_genome                                           95.0%  95.0%    TAAACAATAATAAATTTTACTGTCGTTGACAAGAAACGAGTAACTCGTCCCTCTTCTGCAGACTGCTTACGGTTTCGTCC
 66 AY394998.1_SARS_coronavirus_LC1_complete_genome                                            95.0%  95.0%    TAAACAATAATAAATTTTACTGTCGTTGACAAGAAACGAGTAACTCGTCCCTCTTCTGCAGACTGCTTACGGTTTCGTCC
 67 AY394999.1_SARS_coronavirus_LC2_complete_genome                                            95.0%  95.0%    TAAACAATAATAAATTTTACTGTCGTTGACAAGAAACGAGTAACTCGTCCCTCTTCTGCAGACTGCTTACGGTTTCGTCC
 68 AY395000.1_SARS_coronavirus_LC3_complete_genome                                            95.0%  95.0%    TAAACAATAATAAATTTTACTGTCGTTGACAAGAAACGAGTAACTCGTCCCTCTTCTGCAGACTGCTTACGGTTTCGTCC
 69 AY395001.1_SARS_coronavirus_LC4_complete_genome                                            95.0%  95.0%    TAAACAATAATAAATTTTACTGTCGTTGACAAGAAACGAGTAACTCGTCCCTCTTCTGCAGACTGCTTACGGTTTCGTCC
 70 AY395002.1_SARS_coronavirus_LC5_complete_genome                                            95.0%  95.0%    TAAACAATAATAAATTTTACTGTCGTTGACAAGAAACGAGTAACTCGTCCCTCTTCTGCAGACTGCTTACGGTTTCGTCC
 71 AY395003.1_SARS_coronavirus_ZS-C_complete_genome                                           83.0%  83.0%    TAAACAATAATAAATTTTACTGTCGTTGACAAGAAACGAGTAACTCGTCCCTCTTCTGCAGACTGCTTACGGTTTCGTCC
```

```
 72 AY395004.1_SARS_coronavirus_HZS2-Bb_partial_genome                      84.3%  84.3%    TAAACAATAATAAATTTTACTGTCGTTGACAAGAAACGAGTAACTCGTCCCTCTTCTGCAGACTGCTTACGGTTTCGTCC
 73 AY427439.1_SARS_coronavirus_AS_complete_genome                          94.7%  94.7%    TAAACAATAATAAATTTTACTGTCGTTGACAAGAAACGAGTAACTCGTCCCTCTTCTGCAGACTGCTTACGGTTTCGTCC
 74 AY461660.1_SARS_coronavirus_SoD_complete_genome                         95.0%  94.7%    TAAACAATAATAAATTTTACTGTCGTTGACAAGAAACGAGTAACTCGTCCCTCTTCTGCAGACTGCTTACGGTTTCGTCC
 75 AY463059.1_SARS_coronavirus_ShanghaiQXC1_complete_genome                73.7%  73.7%    TAAACAATAATAAATTTTACTGTCGTTGACAAGAAACGAGTAACTCGTCCCTCTTCTGCAGACTGCTTACGGTTTCGTCC
 76 AY463060.1_SARS_coronavirus_ShanghaiQXC2_complete_genome                73.7%  73.7%    TAAACAATAATAAATTTTACTGTCGTTGACAAGAAACGAGTAACTCGTCCCTCTTCTGCAGACTGCTTACGGTTTCGTCC
 77 AY485277.1_SARS_coronavirus_Sino1-11_complete_genome                   100.0% 100.0%    TAAACAATAATAAATTTTACTGTCGTTGACAAGAAACGAGTAACTCGTCCCTCTTCTGCAGACTGCTTACGGTTTCGTCC
 78 AY485278.1_SARS_coronavirus_Sino3-11_complete_genome                   100.0% 100.0%    TAAACAATAATAAATTTTACTGTCGTTGACAAGAAACGAGTAACTCGTCCCTCTTCTGCAGACTGCTTACGGTTTCGTCC
 79 AY502923.1_SARS_coronavirus_TW10_complete_genome                       100.0% 100.0%    TAAACAATAATAAATTTTACTGTCGTTGACAAGAAACGAGTAACTCGTCCCTCTTCTGCAGACTGCTTACGGTTTCGTCC
 80 AY502924.1_SARS_coronavirus_TW11_complete_genome                       100.0% 100.0%    TAAACAATAATAAATTTTACTGTCGTTGACAAGAAACGAGTAACTCGTCCCTCTTCTGCAGACTGCTTACGGTTTCGTCC
 81 AY502925.1_SARS_coronavirus_TW2_complete_genome                        100.0% 100.0%    TAAACAATAATAAATTTTACTGTCGTTGACAAGAAACGAGTAACTCGTCCCTCTTCTGCAGACTGCTTACGGTTTCGTCC
 82 AY502926.1_SARS_coronavirus_TW3_complete_genome                        100.0% 100.0%    TAAACAATAATAAATTTTACTGTCGTTGACAAGAAACGAGTAACTCGTCCCTCTTCTGCAGACTGCTTACGGTTTCGTCC
 83 AY502927.1_SARS_coronavirus_TW4_complete_genome                        100.0% 100.0%    TAAACAATAATAAATTTTACTGTCGTTGACAAGAAACGAGTAACTCGTCCCTCTTCTGCAGACTGCTTACGGTTTCGTCC
 84 AY502928.1_SARS_coronavirus_TW5_complete_genome                        100.0% 100.0%    TAAACAATAATAAATTTTACTGTCGTTGACAAGAAACGAGTAACTCGTCCCTCTTCTGCAGACTGCTTACGGTTTCGTCC
 85 AY502929.1_SARS_coronavirus_TW6_complete_genome                        100.0% 100.0%    TAAACAATAATAAATTTTACTGTCGTTGACAAGAAACGAGTAACTCGTCCCTCTTCTGCAGACTGCTTACGGTTTCGTCC
 86 AY502930.1_SARS_coronavirus_TW7_complete_genome                        100.0% 100.0%    TAAACAATAATAAATTTTACTGTCGTTGACAAGAAACGAGTAACTCGTCCCTCTTCTGCAGACTGCTTACGGTTTCGTCC
 87 AY502931.1_SARS_coronavirus_TW8_complete_genome                        100.0% 100.0%    TAAACAATAATAAATTTTACTGTCGTTGACAAGAAACGAGTAACTCGTCCCTCTTCTGCAGACTGCTTACGGTTTCGTCC
 88 AY502932.1_SARS_coronavirus_TW9_complete_genome                        100.0% 100.0%    TAAACAATAATAAATTTTACTGTCGTTGACAAGAAACGAGTAACTCGTCCCTCTTCTGCAGACTGCTTACGGTTTCGTCC
 89 AY508724.1_SARS_coronavirus_NS-1_complete_genome                        94.7%  94.7%    TAAACAATAATAAATTTTACTGTCGTTGACAAGAAACGAGTAACTCGTCCCTCTTCTGCAGACTGCTTACGGTTTCGTCC
 90 AY559081.1_SARS_coronavirus_Sin842_complete_genome                      95.7%  95.3%    TAAACAATAATAAATTTTACTGTCGTTGACAAGAAACGAGTAACTCGTCCCTCTTCTGCAGACTGCTTACGGTTTCGTCC
 91 AY559082.1_SARS_coronavirus_Sin852_complete_genome                      99.7%  96.3%    TAAACAATAATAAATTTTACTGTCGTTGACAAGAAACGAGTAACTCGTCCCTCTTCTGCAGACTGCTTACGGTTTCGTCC
 92 AY559083.1_SARS_coronavirus_Sin3408_complete_genome                     88.7%  85.3%    TAAACAATAATAAATTTTACTGTCGTTGACAAGAAACGAGTAACTCGTCCCTCTTCTGCAGACTGCTTACGGTTTCGTCC
 93 AY559084.1_SARS_coronavirus_Sin3765V_complete_genome                    94.7%  94.7%    TAAACAATAATAAATTTTACTGTCGTTGACAAGAAACGAGTAACTCGTCCCTCTTCTGCAGACTGCTTACGGTTTCGTCC
 94 AY559085.1_SARS_coronavirus_Sin848_complete_genome                      94.7%  94.7%    TAAACAATAATAAATTTTACTGTCGTTGACAAGAAACGAGTAACTCGTCCCTCTTCTGCAGACTGCTTACGGTTTCGTCC
 95 AY559086.1_SARS_coronavirus_Sin849_complete_genome                      94.7%  94.7%    TAAACAATAATAAATTTTACTGTCGTTGACAAGAAACGAGTAACTCGTCCCTCTTCTGCAGACTGCTTACGGTTTCGTCC
 96 AY559087.1_SARS_coronavirus_Sin3725V_complete_genome                    94.7%  94.7%    TAAACAATAATAAATTTTACTGTCGTTGACAAGAAACGAGTAACTCGTCCCTCTTCTGCAGACTGCTTACGGTTTCGTCC
 97 AY559088.1_SARS_coronavirus_SinP1_complete_genome                       94.7%  94.7%    TAAACAATAATAAATTTTACTGTCGTTGACAAGAAACGAGTAACTCGTCCCTCTTCTGCAGACTGCTTACGGTTTCGTCC
 98 AY559089.1_SARS_coronavirus_SinP2_complete_genome                       94.7%  94.7%    TAAACAATAATAAATTTTACTGTCGTTGACAAGAAACGAGTAACTCGTCCCTCTTCTGCAGACTGCTTACGGTTTCGTCC
 99 AY559090.1_SARS_coronavirus_SinP3_complete_genome                       95.3%  92.7%    TAAACAATAATAAATTTTACTGTCGTTGACAAGAAACGAGTAACTCGTCCCTCTTCTGCAGACTGCTTACGGTTTCGTCC
100 AY559091.1_SARS_coronavirus_SinP4_complete_genome                       94.7%  94.7%    TAAACAATAATAAATTTTACTGTCGTTGACAAGAAACGAGTAACTCGTCCCTCTTCTGCAGACTGCTTACGGTTTCGTCC
101 AY559092.1_SARS_coronavirus_SinP5_complete_genome                       94.7%  94.7%    TAAACAATAATAAATTTTACTGTCGTTGACAAGAAACGAGTAACTCGTCCCTCTTCTGCAGACTGCTTACGGTTTCGTCC
102 AY559093.1_SARS_coronavirus_Sin845_complete_genome                      94.7%  94.7%    TAAACAATAATAAATTTTACTGTCGTTGACAAGAAACGAGTAACTCGTCCCTCTTCTGCAGACTGCTTACGGTTTCGTCC
103 AY559094.1_SARS_coronavirus_Sin846_complete_genome                      94.7%  94.7%    TAAACAATAATAAATTTTACTGTCGTTGACAAGAAACGAGTAACTCGTCCCTCTTCTGCAGACTGCTTACGGTTTCGTCC
104 AY559095.1_SARS_coronavirus_Sin847_complete_genome                      96.7%  96.0%    TAAACAATAATAAATTTTACTGTCGTTGACAAGAAACGAGTAACTCGTCCCTCTTCTGCAGACTGCTTACGGTTTCGTCC
105 AY559096.1_SARS_coronavirus_Sin850_complete_genome                      97.3%  95.7%    TAAACAATAATAAATTTTACTGTCGTTGACAAGAAACGAGTAACTCGTCCCTCTTCTGCAGACTGCTTACGGTTTCGTCC
106 AY559097.1_SARS_coronavirus_Sin3408L_complete_genome                    94.7%  94.7%    TAAACAATAATAAATTTTACTGTCGTTGACAAGAAACGAGTAACTCGTCCCTCTTCTGCAGACTGCTTACGGTTTCGTCC
107 AY595412.1_SARS_coronavirus_LLJ-2004_complete_genome                    93.0%  93.0%    TAAACAATAATAAATTTTACTGTCGTTGACAAGAAACGAGTAACTCGTCCCTCTTCTGCAGACTGCTTACGGTTTCGTCC
108 AY654624.1_SARS_coronavirus_TJF_complete_genome                         99.7%  99.0%    TAAACAATAATAAATTTTACTGTCGTTGACAAGAAACGAGTAACTCGTCCCTCTTCTGCAGACTGCTTACGGTTTCGTCC
109 AY714217.1_SARS_Coronavirus_CDC*200301157_complete_genome              100.0% 100.0%    TAAACAATAATAAATTTTACTGTCGTTGACAAGAAACGAGTAACTCGTCCCTCTTCTGCAGACTGCTTACGGTTTCGTCC
110 CQ903009.1_Sequence_13_from_Patent_WO2004094667                        100.0%  99.7%    TAAACAATAATAAATTTTACTGTCGTTGACAAGAAACGAGTAACTCGTCCCTCTTCTGCAGACTGCTTACGGTTTCGTCC
111 CQ918584.1_Sequence_1_from_Patent_WO2004096842                          95.0%  95.0%    TAAACAATAATAAATTTTACTGTCGTTGACAAGAAACGAGTAACTCGTCCCTCTTCTGCAGACTGCTTACGGTTTCGTCC
112 CQ918585.1_Sequence_2_from_Patent_WO2004096842                          95.0%  95.0%    TAAACAATAATAAATTTTACTGTCGTTGACAAGAAACGAGTAACTCGTCCCTCTTCTGCAGACTGCTTACGGTTTCGTCC
113 CQ918598.1_Sequence_15_from_Patent_WO2004096842                        100.0% 100.0%    TAAACAATAATAAATTTTACTGTCGTTGACAAGAAACGAGTAACTCGTCCCTCTTCTGCAGACTGCTTACGGTTTCGTCC
114 NC_004718.3_SARS_coronavirus_complete_genome                           100.0% 100.0%    TAAACAATAATAAATTTTACTGTCGTTGACAAGAAACGAGTAACTCGTCCCTCTTCTGCAGACTGCTTACGGTTTCGTCC
    consensus/100%                                                                      ................................................................................
    consensus/90%                                                                       TAAACAATAATAAATTTTACTGTCGTTGACAAGAAACGAGTAACTCGTCCCTCTTCTGCAGACTGCTTACGGTTTCGTCC
    consensus/80%                                                                       TAAACAATAATAAATTTTACTGTCGTTGACAAGAAACGAGTAACTCGTCCCTCTTCTGCAGACTGCTTACGGTTTCGTCC
    consensus/70%                                                                       TAAACAATAATAAATTTTACTGTCGTTGACAAGAAACGAGTAACTCGTCCCTCTTCTGCAGACTGCTTACGGTTTCGTCC

                                                                                 cov    pid 241        :         .         .         .         .         3         .         . 320
  1 AP006557.1_SARS_coronavirus_TWH_genomic_RNA_complete_genome            100.0% 100.0%    GTGTTGCAGTCGATCATCAGCATACCTAGGTTTCGTCCGGGTGTGACCGAAAGGTAAGATGGAGAGCCTTGTTCTTGGTG
  2 AP006558.1_SARS_coronavirus_TWJ_genomic_RNA_complete_genome            100.0% 100.0%    GTGTTGCAGTCGATCATCAGCATACCTAGGTTTCGTCCGGGTGTGACCGAAAGGTAAGATGGAGAGCCTTGTTCTTGGTG
  3 AP006559.1_SARS_coronavirus_TWK_genomic_RNA_complete_genome            100.0% 100.0%    GTGTTGCAGTCGATCATCAGCATACCTAGGTTTCGTCCGGGTGTGACCGAAAGGTAAGATGGAGAGCCTTGTTCTTGGTG
  4 AP006560.1_SARS_coronavirus_TWS_genomic_RNA_complete_genome            100.0% 100.0%    GTGTTGCAGTCGATCATCAGCATACCTAGGTTTCGTCCGGGTGTGACCGAAAGGTAAGATGGAGAGCCTTGTTCTTGGTG
  5 AP006561.1_SARS_coronavirus_TWY_genomic_RNA_complete_genome            100.0% 100.0%    GTGTTGCAGTCGATCATCAGCATACCTAGGTTTCGTCCGGGTGTGACCGAAAGGTAAGATGGAGAGCCTTGTTCTTGGTG
  6 AY274119.3_Severe_acute_respiratory_syndrome-related_coronavirus_isolate_Tor2_complete_genome 100.0% 100.0%    GTGTTGCAGTCGATCATCAGCATACCTAGGTTTCGTCCGGGTGTGACCGAAAGGTAAGATGGAGAGCCTTGTTCTTGGTG
  7 AY278487.3_SARS_coronavirus_BJ02_complete_genome                       100.0% 100.0%    GTGTTGCAGTCGATCATCAGCATACCTAGGTTTCGTCCGGGTGTGACCGAAAGGTAAGATGGAGAGCCTTGTTCTTGGTG
  8 AY278488.2_SARS_coronavirus_BJ01_complete_genome                        93.7%  93.7%    GTGTTGCAGTCGATCATCAGCATACCTAGGTTTCGTCCGGGTGTGACCGAAAGGTAAGATGGAGAGCCTTGTTCTTGGTG
  9 AY278489.2_SARS_coronavirus_GD01_complete_genome                        94.7%  94.7%    GTGTTGCAGTCGATCATCAGCATACCTAGGTTTCGTCCGGGTGTGACCGAAAGGTAAGATGGAGAGCCTTGTTCTTGGTG
 10 AY278490.3_SARS_coronavirus_BJ03_complete_genome                        98.7%  98.7%    GTGTTGCAGTCGATCATCAGCATACCTAGGTTTCGTCCGGGTGTGACCGAAAGGTAAGATGGAGAGCCTTGTTCTTGGTG
 11 AY278491.2_SARS_coronavirus_HKU-39849_complete_genome                  100.0% 100.0%    GTGTTGCAGTCGATCATCAGCATACCTAGGTTTCGTCCGGGTGTGACCGAAAGGTAAGATGGAGAGCCTTGTTCTTGGTG
 12 AY278554.2_SARS_coronavirus_CUHK-W1_complete_genome                     95.0%  95.0%    GTGTTGCAGTCGATCATCAGCATACCTAGGTTTCGTCCGGGTGTGACCGAAAGGTAAGATGGAGAGCCTTGTTCTTGGTG
 13 AY278741.1_SARS_coronavirus_Urbani_complete_genome                     100.0% 100.0%    GTGTTGCAGTCGATCATCAGCATACCTAGGTTTCGTCCGGGTGTGACCGAAAGGTAAGATGGAGAGCCTTGTTCTTGGTG
 14 AY279354.2_SARS_coronavirus_BJ04_complete_genome                        94.7%  94.7%    GTGTTGCAGTCGATCATCAGCATACCTAGGTTTCGTCCGGGTGTGACCGAAAGGTAAGATGGAGAGCCTTGTTCTTGGTG
 15 AY282752.2_SARS_coronavirus_CUHK-Su10_complete_genome                   95.0%  95.0%    GTGTTGCAGTCGATCATCAGCATACCTAGGTTTCGTCCGGGTGTGACCGAAAGGTAAGATGGAGAGCCTTGTTCTTGGTG
 16 AY283794.1_SARS_coronavirus_Sin2500_complete_genome                     94.7%  94.7%    GTGTTGCAGTCGATCATCAGCATACCTAGGTTTCGTCCGGGTGTGACCGAAAGGTAAGATGGAGAGCCTTGTTCTTGGTG
 17 AY283795.1_SARS_coronavirus_Sin2677_complete_genome                     94.7%  94.7%    GTGTTGCAGTCGATCATCAGCATACCTAGGTTTCGTCCGGGTGTGACCGAAAGGTAAGATGGAGAGCCTTGTTCTTGGTG
 18 AY283796.1_SARS_coronavirus_Sin2679_complete_genome                     94.7%  94.7%    GTGTTGCAGTCGATCATCAGCATACCTAGGTTTCGTCCGGGTGTGACCGAAAGGTAAGATGGAGAGCCTTGTTCTTGGTG
 19 AY283797.1_SARS_coronavirus_Sin2748_complete_genome                     94.7%  94.7%    GTGTTGCAGTCGATCATCAGCATACCTAGGTTTCGTCCGGGTGTGACCGAAAGGTAAGATGGAGAGCCTTGTTCTTGGTG
 20 AY283798.2_SARS_coronavirus_Sin2774_complete_genome                     94.7%  94.7%    GTGTTGCAGTCGATCATCAGCATACCTAGGTTTCGTCCGGGTGTGACCGAAAGGTAAGATGGAGAGCCTTGTTCTTGGTG
 21 AY291315.1_SARS_coronavirus_Frankfurt_1_complete_genome                100.0% 100.0%    GTGTTGCAGTCGATCATCAGCATACCTAGGTTTCGTCCGGGTGTGACCGAAAGGTAAGATGGAGAGCCTTGTTCTTGGTG
 22 AY291451.1_SARS_coronavirus_TW1_complete_genome                        100.0% 100.0%    GTGTTGCAGTCGATCATCAGCATACCTAGGTTTCGTCCGGGTGTGACCGAAAGGTAAGATGGAGAGCCTTGTTCTTGGTG
 23 AY297028.1_SARS_coronavirus_ZJ01_complete_genome                        95.3%  95.3%    GTGTTGCAGTCGATCATCAGCATACCTAGGTTTCGTCCGGGTGTGACCGAAAGGTAAGATGGAGAGCCTTGTTCTTGGTG
 24 AY304486.1_SARS_coronavirus_SZ3_complete_genome                         95.0%  95.0%    GTGTTGCAGTCGATCATCAGCATACCTAGGTTTCGTCCGGGTGTGACCGAAAGGTAAGATGGAGAGCCTTGTTCTTGGTG
 25 AY304488.1_SARS_coronavirus_SZ16_complete_genome                        95.0%  95.0%    GTGTTGCAGTCGATCATCAGCATACCTAGGTTTCGTCCGGGTGTGACCGAAAGGTAAGATGGAGAGCCTTGTTCTTGGTG
 26 AY304495.1_SARS_coronavirus_GZ50_complete_genome                        95.3%  95.0%    GTGTTGCAGTCGATCATCAGCATACCTAGGTTTCGTCCGGGTGTGACCGAAAGGTAAGATGGAGAGCCTTGTTCTTGGTG
 27 AY310120.1_SARS_coronavirus_FRA_complete_genome                        100.0% 100.0%    GTGTTGCAGTCGATCATCAGCATACCTAGGTTTCGTCCGGGTGTGACCGAAAGGTAAGATGGAGAGCCTTGTTCTTGGTG
 28 AY313906.1_SARS_coronavirus_GD69_complete_genome                       100.0% 100.0%    GTGTTGCAGTCGATCATCAGCATACCTAGGTTTCGTCCGGGTGTGACCGAAAGGTAAGATGGAGAGCCTTGTTCTTGGTG
 29 AY321118.1_SARS_coronavirus_TWC_complete_genome                        100.0% 100.0%    GTGTTGCAGTCGATCATCAGCATACCTAGGTTTCGTCCGGGTGTGACCGAAAGGTAAGATGGAGAGCCTTGTTCTTGGTG
 30 AY323977.2_SARS_coronavirus_HSR_1_complete_genome                      100.0% 100.0%    GTGTTGCAGTCGATCATCAGCATACCTAGGTTTCGTCCGGGTGTGACCGAAAGGTAAGATGGAGAGCCTTGTTCTTGGTG
 31 AY338174.1_SARS_coronavirus_Taiwan_TC1_complete_genome                  77.0%  77.0%    GTGTTGCAGTCGATCATCAGCATACCTAGGTTTCGTCCGGGTGTGACCGAAAGGTAAGATGGAGAGCCTTGTTCTTGGTG
 32 AY338175.1_SARS_coronavirus_Taiwan_TC2_complete_genome                  77.0%  77.0%    GTGTTGCAGTCGATCATCAGCATACCTAGGTTTCGTCCGGGTGTGACCGAAAGGTAAGATGGAGAGCCTTGTTCTTGGTG
 33 AY345986.1_SARS_coronavirus_CUHK-AG01_complete_genome                   95.0%  95.0%    GTGTTGCAGTCGATCATCAGCATACCTAGGTTTCGTCCGGGTGTGACCGAAAGGTAAGATGGAGAGCCTTGTTCTTGGTG
 34 AY345987.1_SARS_coronavirus_CUHK-AG02_complete_genome                   95.0%  95.0%    GTGTTGCAGTCGATCATCAGCATACCTAGGTTTCGTCCGGGTGTGACCGAAAGGTAAGATGGAGAGCCTTGTTCTTGGTG
 35 AY345988.1_SARS_coronavirus_CUHK-AG03_complete_genome                   95.0%  95.0%    GTGTTGCAGTCGATCATCAGCATACCTAGGTTTCGTCCGGGTGTGACCGAAAGGTAAGATGGAGAGCCTTGTTCTTGGTG
 36 AY348314.1_SARS_coronavirus_Taiwan_TC3_complete_genome                  77.0%  77.0%    GTGTTGCAGTCGATCATCAGCATACCTAGGTTTCGTCCGGGTGTGACCGAAAGGTAAGATGGAGAGCCTTGTTCTTGGTG
 37 AY350750.1_SARS_coronavirus_PUMC01_complete_genome                      95.7%  95.7%    GTGTTGCAGTCGATCATCAGCATACCTAGGTTTCGTCCGGGTGTGACCGAAAGGTAAGATGGAGAGCCTTGTTCTTGGTG
 38 AY351680.1_SARS_coronavirus_ZMY_1_complete_genome                      100.0%  99.7%    GTGTTGCAGTCGATCATCAGCATACCTAGGTTTCGTCCGGGTGTGACCGAAAGGTAAGATGGAGAGCCTTGTTCTTGGTG
 39 AY357075.1_SARS_coronavirus_PUMC02_complete_genome                      95.3%  95.3%    GTGTTGCAGTCGATCATCAGCATACCTAGGTTTCGTCCGGGTGTGACCGAAAGGTAAGATGGAGAGCCTTGTTCTTGGTG
 40 AY357076.1_SARS_coronavirus_PUMC03_complete_genome                      95.3%  95.3%    GTGTTGCAGTCGATCATCAGCATACCTAGGTTTCGTCCGGGTGTGACCGAAAGGTAAGATGGAGAGCCTTGTTCTTGGTG
 41 AY362698.1_SARS_coronavirus_TWC2_complete_genome                       100.0% 100.0%    GTGTTGCAGTCGATCATCAGCATACCTAGGTTTCGTCCGGGTGTGACCGAAAGGTAAGATGGAGAGCCTTGTTCTTGGTG
 42 AY362699.1_SARS_coronavirus_TWC3_complete_genome                       100.0% 100.0%    GTGTTGCAGTCGATCATCAGCATACCTAGGTTTCGTCCGGGTGTGACCGAAAGGTAAGATGGAGAGCCTTGTTCTTGGTG
 43 AY390556.1_SARS_coronavirus_GZ02_complete_genome                       100.0% 100.0%    GTGTTGCAGTCGATCATCAGCATACCTAGGTTTCGTCCGGGTGTGACCGAAAGGTAAGATGGAGAGCCTTGTTCTTGGTG
 44 AY394850.2_SARS_coronavirus_WHU_complete_genome                        100.0% 100.0%    GTGTTGCAGTCGATCATCAGCATACCTAGGTTTCGTCCGGGTGTGACCGAAAGGTAAGATGGAGAGCCTTGTTCTTGGTG
 45 AY394977.1_SARS_coronavirus_GZ-A_partial_genome                         83.0%  83.0%    GTGTTGCAGTCGATCATCAGCATACCTAGGTTTCGTCCGGGTGTGACCGAAAGGTAAGATGGAGAGCCTTGTTCTTGGTG
 46 AY394978.1_SARS_coronavirus_GZ-B_complete_genome                        76.0%  76.0%    GTGTTGCAGTCGATCATCAGCATACCTAGGTTTCGTCCGGGTGTGACCGAAAGGTAAGATGGAGAGCCTTGTTCTTGGTG
 47 AY394979.1_SARS_coronavirus_GZ-C_complete_genome                        82.7%  82.7%    GTGTTGCAGTCGATCATCAGCATACCTAGGTTTCGTCCGGGTGTGACCGAAAGGTAAGATGGAGAGCCTTGTTCTTGGTG
 48 AY394980.1_SARS_coronavirus_GZ-D_partial_genome                         84.7%  84.3%    GTGTTGCAGTCGATCATCAGCATACCTAGGTTTCGTCCGGGTGTGACCGAAAGGTAAGATGGAGAGCCTTGTTCTTGGTG
 49 AY394981.1_SARS_coronavirus_HGZ8L1-A_partial_genome                     83.0%  83.0%    GTGTTGCAGTCGATCATCAGCATACCTAGGTTTCGTCCGGGTGTGACCGAAAGGTAAGATGGAGAGCCTTGTTCTTGGTG
 50 AY394982.1_SARS_coronavirus_HGZ8L1-B_partial_genome                     82.7%  82.7%    GTGTTGCAGTCGATCATCAGCATACCTAGGTTTCGTCCGGGTGTGACCGAAAGGTAAGATGGAGAGCCTTGTTCTTGGTG
 51 AY394983.1_SARS_coronavirus_HSZ2-A_complete_genome                      82.7%  82.7%    GTGTTGCAGTCGATCATCAGCATACCTAGGTTTCGTCCGGGTGTGACCGAAAGGTAAGATGGAGAGCCTTGTTCTTGGTG
 52 AY394984.1_SARS_coronavirus_HSZ-A_partial_genome                        84.3%  84.3%    GTGTTGCAGTCGATCATCAGCATACCTAGGTTTCGTCCGGGTGTGACCGAAAGGTAAGATGGAGAGCCTTGTTCTTGGTG
 53 AY394985.1_SARS_coronavirus_HSZ-Bb_partial_genome                       16.7%  16.7%    --------------------------------------------GACCGAAAGGTAAGATGGAGAGCCTTGTTCTTGGTG
 54 AY394986.1_SARS_coronavirus_HSZ-Cb_complete_genome                      83.0%  83.0%    GTGTTGCAGTCGATCATCAGCATACCTAGGTTTCGTCCGGGTGTGACCGAAAGGTAAGATGGAGAGCCTTGTTCTTGGTG
 55 AY394987.1_SARS_coronavirus_HZS2-Fb_complete_genome                     86.0%  86.0%    GTGTTGCAGTCGATCATCAGCATACCTAGGTTTCGTCCGGGTGTGACCGAAAGGTAAGATGGAGAGCCTTGTTCTTGGTG
 56 AY394988.1_SARS_coronavirus_JMD_partial_genome                          71.3%  71.0%    GTGTTGCAGTCGATCATCAGCATACCTAGGTTTCGTCCGGGTGTGACCGAAAGGTAAGATGGAGAGCCTTGTTCTTGGTG
 57 AY394989.1_SARS_coronavirus_HZS2-D_complete_genome                      95.0%  95.0%    GTGTTGCAGTCGATCATCAGCATACCTAGGTTTCGTCCGGGTGTGACCGAAAGGTAAGATGGAGAGCCTTGTTCTTGGTG
 58 AY394990.1_SARS_coronavirus_HZS2-E_complete_genome                      95.0%  95.0%    GTGTTGCAGTCGATCATCAGCATACCTAGGTTTCGTCCGGGTGTGACCGAAAGGTAAGATGGAGAGCCTTGTTCTTGGTG
 59 AY394991.1_SARS_coronavirus_HZS2-Fc_complete_genome                     95.0%  95.0%    GTGTTGCAGTCGATCATCAGCATACCTAGGTTTCGTCCGGGTGTGACCGAAAGGTAAGATGGAGAGCCTTGTTCTTGGTG
 60 AY394992.1_SARS_coronavirus_HZS2-C_complete_genome                      95.0%  95.0%    GTGTTGCAGTCGATCATCAGCATACCTAGGTTTCGTCCGGGTGTGACCGAAAGGTAAGATGGAGAGCCTTGTTCTTGGTG
 61 AY394993.1_SARS_coronavirus_HGZ8L2_complete_genome                      95.0%  95.0%    GTGTTGCAGTCGATCATCAGCATACCTAGGTTTCGTCCGGGTGTGACCGAAAGGTAAGATGGAGAGCCTTGTTCTTGGTG
 62 AY394994.1_SARS_coronavirus_HSZ-Bc_complete_genome                      95.0%  95.0%    GTGTTGCAGTCGATCATCAGCATACCTAGGTTTCGTCCGGGTGTGACCGAAAGGTAAGATGGAGAGCCTTGTTCTTGGTG
 63 AY394995.1_SARS_coronavirus_HSZ-Cc_complete_genome                      95.0%  95.0%    GTGTTGCAGTCGATCATCAGCATACCTAGGTTTCGTCCGGGTGTGACCGAAAGGTAAGATGGAGAGCCTTGTTCTTGGTG
 64 AY394996.1_SARS_coronavirus_ZS-B_complete_genome                        95.0%  95.0%    GTGTTGCAGTCGATCATCAGCATACCTAGGTTTCGTCCGGGTGTGACCGAAAGGTAAGATGGAGAGCCTTGTTCTTGGTG
 65 AY394997.1_SARS_coronavirus_ZS-A_complete_genome                        95.0%  95.0%    GTGTTGCAGTCGATCATCAGCATACCTAGGTTTCGTCCGGGTGTGACCGAAAGGTAAGATGGAGAGCCTTGTTCTTGGTG
 66 AY394998.1_SARS_coronavirus_LC1_complete_genome                         95.0%  95.0%    GTGTTGCAGTCGATCATCAGCATACCTAGGTTTCGTCCGGGTGTGACCGAAAGGTAAGATGGAGAGCCTTGTTCTTGGTG
 67 AY394999.1_SARS_coronavirus_LC2_complete_genome                         95.0%  95.0%    GTGTTGCAGTCGATCATCAGCATACCTAGGTTTCGTCCGGGTGTGACCGAAAGGTAAGATGGAGAGCCTTGTTCTTGGTG
 68 AY395000.1_SARS_coronavirus_LC3_complete_genome                         95.0%  95.0%    GTGTTGCAGTCGATCATCAGCATACCTAGGTTTCGTCCGGGTGTGACCGAAAGGTAAGATGGAGAGCCTTGTTCTTGGTG
 69 AY395001.1_SARS_coronavirus_LC4_complete_genome                         95.0%  95.0%    GTGTTGCAGTCGATCATCAGCATACCTAGGTTTCGTCCGGGTGTGACCGAAAGGTAAGATGGAGAGCCTTGTTCTTGGTG
 70 AY395002.1_SARS_coronavirus_LC5_complete_genome                         95.0%  95.0%    GTGTTGCAGTCGATCATCAGCATACCTAGGTTTCGTCCGGGTGTGACCGAAAGGTAAGATGGAGAGCCTTGTTCTTGGTG
 71 AY395003.1_SARS_coronavirus_ZS-C_complete_genome                        83.0%  83.0%    GTGTTGCAGTCGATCATCAGCATACCTAGGTTTCGTCCGGGTGTGACCGAAAGGTAAGATGGAGAGCCTTGTTCTTGGTG
 72 AY395004.1_SARS_coronavirus_HZS2-Bb_partial_genome                      84.3%  84.3%    GTGTTGCAGTCGATCATCAGCATACCTAGGTTTCGTCCGGGTGTGACCGAAAGGTAAGATGGAGAGCCTTGTTCTTGGTG
 73 AY427439.1_SARS_coronavirus_AS_complete_genome                          94.7%  94.7%    GTGTTGCAGTCGATCATCAGCATACCTAGGTTTCGTCCGGGTGTGACCGAAAGGTAAGATGGAGAGCCTTGTTCTTGGTG
 74 AY461660.1_SARS_coronavirus_SoD_complete_genome                         95.0%  94.7%    GTGTTGCAGTCGATCATCAGCATACCTAGGTTTCGTCCGGGTGTGACCGAAAGGTAAGATGGAGAGCCTTGTTCTTGGTG
 75 AY463059.1_SARS_coronavirus_ShanghaiQXC1_complete_genome                73.7%  73.7%    GTGTTGCAGTCGATCATCAGCATACCTAGGTTTCGTCCGGGTGTGACCGAAAGGTAAGATGGAGAGCCTTGTTCTTGGTG
 76 AY463060.1_SARS_coronavirus_ShanghaiQXC2_complete_genome                73.7%  73.7%    GTGTTGCAGTCGATCATCAGCATACCTAGGTTTCGTCCGGGTGTGACCGAAAGGTAAGATGGAGAGCCTTGTTCTTGGTG
 77 AY485277.1_SARS_coronavirus_Sino1-11_complete_genome                   100.0% 100.0%    GTGTTGCAGTCGATCATCAGCATACCTAGGTTTCGTCCGGGTGTGACCGAAAGGTAAGATGGAGAGCCTTGTTCTTGGTG
 78 AY485278.1_SARS_coronavirus_Sino3-11_complete_genome                   100.0% 100.0%    GTGTTGCAGTCGATCATCAGCATACCTAGGTTTCGTCCGGGTGTGACCGAAAGGTAAGATGGAGAGCCTTGTTCTTGGTG
 79 AY502923.1_SARS_coronavirus_TW10_complete_genome                       100.0% 100.0%    GTGTTGCAGTCGATCATCAGCATACCTAGGTTTCGTCCGGGTGTGACCGAAAGGTAAGATGGAGAGCCTTGTTCTTGGTG
 80 AY502924.1_SARS_coronavirus_TW11_complete_genome                       100.0% 100.0%    GTGTTGCAGTCGATCATCAGCATACCTAGGTTTCGTCCGGGTGTGACCGAAAGGTAAGATGGAGAGCCTTGTTCTTGGTG
 81 AY502925.1_SARS_coronavirus_TW2_complete_genome                        100.0% 100.0%    GTGTTGCAGTCGATCATCAGCATACCTAGGTTTCGTCCGGGTGTGACCGAAAGGTAAGATGGAGAGCCTTGTTCTTGGTG
 82 AY502926.1_SARS_coronavirus_TW3_complete_genome                        100.0% 100.0%    GTGTTGCAGTCGATCATCAGCATACCTAGGTTTCGTCCGGGTGTGACCGAAAGGTAAGATGGAGAGCCTTGTTCTTGGTG
 83 AY502927.1_SARS_coronavirus_TW4_complete_genome                        100.0% 100.0%    GTGTTGCAGTCGATCATCAGCATACCTAGGTTTCGTCCGGGTGTGACCGAAAGGTAAGATGGAGAGCCTTGTTCTTGGTG
 84 AY502928.1_SARS_coronavirus_TW5_complete_genome                        100.0% 100.0%    GTGTTGCAGTCGATCATCAGCATACCTAGGTTTCGTCCGGGTGTGACCGAAAGGTAAGATGGAGAGCCTTGTTCTTGGTG
 85 AY502929.1_SARS_coronavirus_TW6_complete_genome                        100.0% 100.0%    GTGTTGCAGTCGATCATCAGCATACCTAGGTTTCGTCCGGGTGTGACCGAAAGGTAAGATGGAGAGCCTTGTTCTTGGTG
 86 AY502930.1_SARS_coronavirus_TW7_complete_genome                        100.0% 100.0%    GTGTTGCAGTCGATCATCAGCATACCTAGGTTTCGTCCGGGTGTGACCGAAAGGTAAGATGGAGAGCCTTGTTCTTGGTG
 87 AY502931.1_SARS_coronavirus_TW8_complete_genome                        100.0% 100.0%    GTGTTGCAGTCGATCATCAGCATACCTAGGTTTCGTCCGGGTGTGACCGAAAGGTAAGATGGAGAGCCTTGTTCTTGGTG
 88 AY502932.1_SARS_coronavirus_TW9_complete_genome                        100.0% 100.0%    GTGTTGCAGTCGATCATCAGCATACCTAGGTTTCGTCCGGGTGTGACCGAAAGGTAAGATGGAGAGCCTTGTTCTTGGTG
 89 AY508724.1_SARS_coronavirus_NS-1_complete_genome                        94.7%  94.7%    GTGTTGCAGTCGATCATCAGCATACCTAGGTTTCGTCCGGGTGTGACCGAAAGGTAAGATGGAGAGCCTTGTTCTTGGTG
 90 AY559081.1_SARS_coronavirus_Sin842_complete_genome                      95.7%  95.3%    GTGTTGCAGTCGATCATCAGCATACCTAGGTTTCGTCCGGGTGTGACCGAAAGGTAAGATGGAGAGCCTTGTTCTTGGTG
 91 AY559082.1_SARS_coronavirus_Sin852_complete_genome                      99.7%  96.3%    GTGTTGCAGTCGATCATCAGCATACCTAGGTTTCGTCCGGGTGTGACCGAAAGGTAAGATGGAGAGCCTTGTTCTTGGTG
 92 AY559083.1_SARS_coronavirus_Sin3408_complete_genome                     88.7%  85.3%    GTGTTGCAGTCGATCATCAGCATACCTAGGTTTCGTCCGGGTGTGACCGAAAGGTAAGAT--------------------
 93 AY559084.1_SARS_coronavirus_Sin3765V_complete_genome                    94.7%  94.7%    GTGTTGCAGTCGATCATCAGCATACCTAGGTTTCGTCCGGGTGTGACCGAAAGGTAAGATGGAGAGCCTTGTTCTTGGTG
 94 AY559085.1_SARS_coronavirus_Sin848_complete_genome                      94.7%  94.7%    GTGTTGCAGTCGATCATCAGCATACCTAGGTTTCGTCCGGGTGTGACCGAAAGGTAAGATGGAGAGCCTTGTTCTTGGTG
 95 AY559086.1_SARS_coronavirus_Sin849_complete_genome                      94.7%  94.7%    GTGTTGCAGTCGATCATCAGCATACCTAGGTTTCGTCCGGGTGTGACCGAAAGGTAAGATGGAGAGCCTTGTTCTTGGTG
 96 AY559087.1_SARS_coronavirus_Sin3725V_complete_genome                    94.7%  94.7%    GTGTTGCAGTCGATCATCAGCATACCTAGGTTTCGTCCGGGTGTGACCGAAAGGTAAGATGGAGAGCCTTGTTCTTGGTG
 97 AY559088.1_SARS_coronavirus_SinP1_complete_genome                       94.7%  94.7%    GTGTTGCAGTCGATCATCAGCATACCTAGGTTTCGTCCGGGTGTGACCGAAAGGTAAGATGGAGAGCCTTGTTCTTGGTG
 98 AY559089.1_SARS_coronavirus_SinP2_complete_genome                       94.7%  94.7%    GTGTTGCAGTCGATCATCAGCATACCTAGGTTTCGTCCGGGTGTGACCGAAAGGTAAGATGGAGAGCCTTGTTCTTGGTG
 99 AY559090.1_SARS_coronavirus_SinP3_complete_genome                       95.3%  92.7%    GTGTTGCAGTCGATCATCAGCATACCTAGGTTTCGTCCGGGTGTGACCGAAAGGTAAGATGGAGAGCCTTGTTCTTGGTG
100 AY559091.1_SARS_coronavirus_SinP4_complete_genome                       94.7%  94.7%    GTGTTGCAGTCGATCATCAGCATACCTAGGTTTCGTCCGGGTGTGACCGAAAGGTAAGATGGAGAGCCTTGTTCTTGGTG
101 AY559092.1_SARS_coronavirus_SinP5_complete_genome                       94.7%  94.7%    GTGTTGCAGTCGATCATCAGCATACCTAGGTTTCGTCCGGGTGTGACCGAAAGGTAAGATGGAGAGCCTTGTTCTTGGTG
102 AY559093.1_SARS_coronavirus_Sin845_complete_genome                      94.7%  94.7%    GTGTTGCAGTCGATCATCAGCATACCTAGGTTTCGTCCGGGTGTGACCGAAAGGTAAGATGGAGAGCCTTGTTCTTGGTG
103 AY559094.1_SARS_coronavirus_Sin846_complete_genome                      94.7%  94.7%    GTGTTGCAGTCGATCATCAGCATACCTAGGTTTCGTCCGGGTGTGACCGAAAGGTAAGATGGAGAGCCTTGTTCTTGGTG
104 AY559095.1_SARS_coronavirus_Sin847_complete_genome                      96.7%  96.0%    GTGTTGCAGTCGATCATCAGCATACCTAGGTTTCGTCCGGGTGTGACCGAAAGGTAAGATGGAGAGCCTTGTTCTTGGTG
105 AY559096.1_SARS_coronavirus_Sin850_complete_genome                      97.3%  95.7%    GTGTTGCAGTCGATCATCAGCATACCTAGGTTTCGTCCGGGTGTGACCGAAAGGTAAGATGGAGAGCCTTGTTCTTGGTG
106 AY559097.1_SARS_coronavirus_Sin3408L_complete_genome                    94.7%  94.7%    GTGTTGCAGTCGATCATCAGCATACCTAGGTTTCGTCCGGGTGTGACCGAAAGGTAAGATGGAGAGCCTTGTTCTTGGTG
107 AY595412.1_SARS_coronavirus_LLJ-2004_complete_genome                    93.0%  93.0%    GTGTTGCAGTCGATCATCAGCATACCTAGGTTTCGTCCGGGTGTGACCGAAAGGTAAGATGGAGAGCCTTGTTCTTGGTG
108 AY654624.1_SARS_coronavirus_TJF_complete_genome                         99.7%  99.0%    GTGTTGCAGTCGATCATCAGCATACCTAGGTTTCGTCCGGGTGTGACCGAAAGGTAAGATGGAGAGCCTTGTTCTTGGTG
109 AY714217.1_SARS_Coronavirus_CDC*200301157_complete_genome              100.0% 100.0%    GTGTTGCAGTCGATCATCAGCATACCTAGGTTTCGTCCGGGTGTGACCGAAAGGTAAGATGGAGAGCCTTGTTCTTGGTG
110 CQ903009.1_Sequence_13_from_Patent_WO2004094667                        100.0%  99.7%    GTGTTGCAGTCGATCATCAGCATACCTAGGTTTCGTCCGGGTGTGACCGAAAGGTAAGATGGAGAGCCTTGTTCTTGGTG
```

```
111 CQ918584.1_Sequence_1_from_Patent_WO2004096842                                   95.0%  95.0%     GTGTTGCAGTCGATCATCAGCATACCTAGGTTTCGTCCGGGTGTGACCGAAAGGTAAGATGGAGAGCCTTGTTCTTGGTG
112 CQ918585.1_Sequence_2_from_Patent_WO2004096842                                   95.0%  95.0%     GTGTTGCAGTCGATCATCAGCATACCTAGGTTTCGTCCGGGTGTGACCGAAAGGTAAGATGGAGAGCCTTGTTCTTGGTG
113 CQ918598.1_Sequence_15_from_Patent_WO2004096842                                 100.0% 100.0%     GTGTTGCAGTCGATCATCAGCATACCTAGGTTTCGTCCGGGTGTGACCGAAAGGTAAGATGGAGAGCCTTGTTCTTGGTG
114 NC_004718.3_SARS_coronavirus_complete_genome                                    100.0% 100.0%     GTGTTGCAGTCGATCATCAGCATACCTAGGTTTCGTCCGGGTGTGACCGAAAGGTAAGATGGAGAGCCTTGTTCTTGGTG
    consensus/100%                                                                                 ............................................GACCGAAAGGTAAGAT....................
    consensus/90%                                                                                  GTGTTGCAGTCGATCATCAGCATACCTAGGTTTCGTCCGGGTGTGACCGAAAGGTAAGATGGAGAGCCTTGTTCTTGGTG
    consensus/80%                                                                                  GTGTTGCAGTCGATCATCAGCATACCTAGGTTTCGTCCGGGTGTGACCGAAAGGTAAGATGGAGAGCCTTGTTCTTGGTG
    consensus/70%                                                                                  GTGTTGCAGTCGATCATCAGCATACCTAGGTTTCGTCCGGGTGTGACCGAAAGGTAAGATGGAGAGCCTTGTTCTTGGTG

                                                                                     cov    pid 321         .         .         :         .         .         .         .         4 400
  1 AP006557.1_SARS_coronavirus_TWH_genomic_RNA_complete_genome                      100.0% 100.0%     TCAACGAGAAAACA------------------------------------------------------------------
  2 AP006558.1_SARS_coronavirus_TWJ_genomic_RNA_complete_genome                      100.0% 100.0%     TCAACGAGAAAACA------------------------------------------------------------------
  3 AP006559.1_SARS_coronavirus_TWK_genomic_RNA_complete_genome                      100.0% 100.0%     TCAACGAGAAAACA------------------------------------------------------------------
  4 AP006560.1_SARS_coronavirus_TWS_genomic_RNA_complete_genome                      100.0% 100.0%     TCAACGAGAAAACA------------------------------------------------------------------
  5 AP006561.1_SARS_coronavirus_TWY_genomic_RNA_complete_genome                      100.0% 100.0%     TCAACGAGAAAACA------------------------------------------------------------------
  6 AY274119.3_Severe_acute_respiratory_syndrome-related_coronavirus_isolate_Tor2_complete_genome 100.0% 100.0%     TCAACGAGAAAACA------------------------------------------------------------------
  7 AY278487.3_SARS_coronavirus_BJ02_complete_genome                                100.0% 100.0%     TCAACGAGAAAACA------------------------------------------------------------------
  8 AY278488.2_SARS_coronavirus_BJ01_complete_genome                                 93.7%  93.7%     TCAACGAGAAAACACACGTCCAACTCAGTTTGC-----------------------------------------------
  9 AY278489.2_SARS_coronavirus_GD01_complete_genome                                 94.7%  94.7%     TCAACGAGAAAACACACGTCCAACTCAGTT--------------------------------------------------
 10 AY278490.3_SARS_coronavirus_BJ03_complete_genome                                 98.7%  98.7%     TCAACGAGAAAACACACG--------------------------------------------------------------
 11 AY278491.2_SARS_coronavirus_HKU-39849_complete_genome                           100.0% 100.0%     TCAACGAGAAAACA------------------------------------------------------------------
 12 AY278554.2_SARS_coronavirus_CUHK-W1_complete_genome                              95.0%  95.0%     TCAACGAGAAAACACACGTCCAACTCAGT---------------------------------------------------
 13 AY278741.1_SARS_coronavirus_Urbani_complete_genome                              100.0% 100.0%     TCAACGAGAAAACA------------------------------------------------------------------
 14 AY279354.2_SARS_coronavirus_BJ04_complete_genome                                 94.7%  94.7%     TCAACGAGAAAACACACGTCCAACTCAGTT--------------------------------------------------
 15 AY282752.2_SARS_coronavirus_CUHK-Su10_complete_genome                            95.0%  95.0%     TCAACGAGAAAACACACGTCCAACTCAGT---------------------------------------------------
 16 AY283794.1_SARS_coronavirus_Sin2500_complete_genome                              94.7%  94.7%     TCAACGAGAAAACACACGTCCAACTCAGTT--------------------------------------------------
 17 AY283795.1_SARS_coronavirus_Sin2677_complete_genome                              94.7%  94.7%     TCAACGAGAAAACACACGTCCAACTCAGTT--------------------------------------------------
 18 AY283796.1_SARS_coronavirus_Sin2679_complete_genome                              94.7%  94.7%     TCAACGAGAAAACACACGTCCAACTCAGTT--------------------------------------------------
 19 AY283797.1_SARS_coronavirus_Sin2748_complete_genome                              94.7%  94.7%     TCAACGAGAAAACACACGTCCAACTCAGTT--------------------------------------------------
 20 AY283798.2_SARS_coronavirus_Sin2774_complete_genome                              94.7%  94.7%     TCAACGAGAAAACACACGTCCAACTCAGTT--------------------------------------------------
 21 AY291315.1_SARS_coronavirus_Frankfurt_1_complete_genome                         100.0% 100.0%     TCAACGAGAAAACA------------------------------------------------------------------
 22 AY291451.1_SARS_coronavirus_TW1_complete_genome                                 100.0% 100.0%     TCAACGAGAAAACA------------------------------------------------------------------
 23 AY297028.1_SARS_coronavirus_ZJ01_complete_genome                                 95.3%  95.3%     TCAACGAGAAAACACACGTCCAACTCAG----------------------------------------------------
 24 AY304486.1_SARS_coronavirus_SZ3_complete_genome                                  95.0%  95.0%     TCAACGAGAAAACACACGTCCAACTCAGT---------------------------------------------------
 25 AY304488.1_SARS_coronavirus_SZ16_complete_genome                                 95.0%  95.0%     TCAACGAGAAAACACACGTCCAACTCAGT---------------------------------------------------
 26 AY304495.1_SARS_coronavirus_GZ50_complete_genome                                 95.0%  95.0%     TCAACGAGAAAACACACGTCCAACTCAGT---------------------------------------------------
 27 AY310120.1_SARS_coronavirus_FRA_complete_genome                                 100.0% 100.0%     TCAACGAGAAAACA------------------------------------------------------------------
 28 AY313906.1_SARS_coronavirus_GD69_complete_genome                                100.0% 100.0%     TCAACGAGAAAACA------------------------------------------------------------------
 29 AY321118.1_SARS_coronavirus_TWC_complete_genome                                 100.0% 100.0%     TCAACGAGAAAACA------------------------------------------------------------------
 30 AY323977.2_SARS_coronavirus_HSR_1_complete_genome                               100.0% 100.0%     TCAACGAGAAAACA------------------------------------------------------------------
 31 AY338174.1_SARS_coronavirus_Taiwan_TC1_complete_genome                           77.0%  77.0%     TCAACGAGAAAACACACGTCCAACTCAGTTTGCCTGTCCTTCAGGTTAGAGACGTGCTAGTGCGTGGCTTCGGGGACTCT
 32 AY338175.1_SARS_coronavirus_Taiwan_TC2_complete_genome                           77.0%  77.0%     TCAACGAGAAAACACACGTCCAACTCAGTTTGCCTGTCCTTCAGGTTAGAGACGTGCTAGTGCGTGGCTTCGGGGACTCT
 33 AY345986.1_SARS_coronavirus_CUHK-AG01_complete_genome                            95.0%  95.0%     TCAACGAGAAAACACACGTCCAACTCAGT---------------------------------------------------
 34 AY345987.1_SARS_coronavirus_CUHK-AG02_complete_genome                            95.0%  95.0%     TCAACGAGAAAACACACGTCCAACTCAGT---------------------------------------------------
 35 AY345988.1_SARS_coronavirus_CUHK-AG03_complete_genome                            95.0%  95.0%     TCAACGAGAAAACACACGTCCAACTCAGT---------------------------------------------------
 36 AY348314.1_SARS_coronavirus_Taiwan_TC3_complete_genome                           77.0%  77.0%     TCAACGAGAAAACACACGTCCAACTCAGTTTGCCTGTCCTTCAGGTTAGAGACGTGCTAGTGCGTGGCTTCGGGGACTCT
 37 AY350750.1_SARS_coronavirus_PUMC01_complete_genome                               95.7%  95.7%     TCAACGAGAAAACACACGTCCAACTCA-----------------------------------------------------
 38 AY351680.1_SARS_coronavirus_ZMY_1_complete_genome                               100.0%  99.7%     TCAACGAGAAAACA------------------------------------------------------------------
 39 AY357075.1_SARS_coronavirus_PUMC02_complete_genome                               95.3%  95.3%     TCAACGAGAAAACACACGTCCAACTCAG----------------------------------------------------
 40 AY357076.1_SARS_coronavirus_PUMC03_complete_genome                               95.3%  95.3%     TCAACGAGAAAACACACGTCCAACTCAG----------------------------------------------------
 41 AY362698.1_SARS_coronavirus_TWC2_complete_genome                                100.0% 100.0%     TCAACGAGAAAACA------------------------------------------------------------------
 42 AY362699.1_SARS_coronavirus_TWC3_complete_genome                                100.0% 100.0%     TCAACGAGAAAACA------------------------------------------------------------------
 43 AY390556.1_SARS_coronavirus_GZ02_complete_genome                                100.0% 100.0%     TCAACGAGAAAACA------------------------------------------------------------------
 44 AY394850.2_SARS_coronavirus_WHU_complete_genome                                 100.0% 100.0%     TCAACGAGAAAACA------------------------------------------------------------------
 45 AY394977.1_SARS_coronavirus_GZ-A_partial_genome                                  83.0%  83.0%     TCAACGAGAAAACACACGTCCAACTCAGTTTGCCTGTCCTTCAGGTTAGAGACGTGCTAGTGCGT---------------
 46 AY394978.1_SARS_coronavirus_GZ-B_complete_genome                                 76.0%  76.0%     TCAACGAGAAAACACACGTCCAACTCAGTTTGCCTGTCCTTCAGGTTAGAGACGTGCTAGTGCGTGGCTTCGGGGACTCT
 47 AY394979.1_SARS_coronavirus_GZ-C_complete_genome                                 82.7%  82.7%     TCAACGAGAAAACACACGTCCAACTCAGTTTGCCTGTCCTTCAGGTTAGAGACGTGCTAGTGCGTG--------------
 48 AY394980.1_SARS_coronavirus_GZ-D_partial_genome                                  84.7%  84.3%     TCAACGAGAAAACACACGTCCAACTCAGTTTGCCTGTCCTTCAGGTTAGAGACGTGCTAG--------------------
 49 AY394981.1_SARS_coronavirus_HGZ8L1-A_partial_genome                              83.0%  83.0%     TCAACGAGAAAACACACGTCCAACTCAGTTTGCCTGTCCTTCAGGTTAGAGACGTGCTAGTGCGT---------------
 50 AY394982.1_SARS_coronavirus_HGZ8L1-B_partial_genome                              82.7%  82.7%     TCAACGAGAAAACACACGTCCAACTCAGTTTGCCTGTCCTTCAGGTTAGAGACGTGCTAGTGCGTG--------------
 51 AY394983.1_SARS_coronavirus_HSZ2-A_complete_genome                               82.7%  82.7%     TCAACGAGAAAACACACGTCCAACTCAGTTTGCCTGTCCTTCAGGTTAGAGACGTGCTAGTGCGTG--------------
 52 AY394984.1_SARS_coronavirus_HSZ-A_partial_genome                                 84.3%  84.3%     TCAACGAGAAAACACACGTCCAACTCAGTTTGCCTGTCCTTCAGGTTAGAGACGTGCTAGT-------------------
 53 AY394985.1_SARS_coronavirus_HSZ-Bb_complete_genome                               16.7%  16.7%     TCAACGAGAAAACACACGTCCAACTCAGTTTGCCTGTCCTTCAGGTTAGAGACGTGCTAGTGCGTGGCTTCGGGGACTCT
 54 AY394986.1_SARS_coronavirus_HSZ-Cb_complete_genome                               83.0%  83.0%     TCAACGAGAAAACACACGTCCAACTCAGTTTGCCTGTCCTTCAGGTTAGAGACGTGCTAGTGCGT---------------
 55 AY394987.1_SARS_coronavirus_HZS2-Fb_complete_genome                              86.0%  86.0%     TCAACGAGAAAACACACGTCCAACTCAGTTTGCCTGTCCTTCAGGTTAGAGACGTG------------------------
 56 AY394988.1_SARS_coronavirus_JMD_partial_genome                                   71.3%  71.0%     TCAACGAGAAAACACACGTCCAACTCAGTTTGCCTGTCCTTCAGGTTAGAGACGTGCTAGTGCGTGGCTTCGGGGACTCT
 57 AY394989.1_SARS_coronavirus_HZS2-D_complete_genome                               95.0%  95.0%     TCAACGAGAAAACACACGTCCAACTCAGT---------------------------------------------------
 58 AY394990.1_SARS_coronavirus_HZS2-E_complete_genome                               95.0%  95.0%     TCAACGAGAAAACACACGTCCAACTCAGT---------------------------------------------------
 59 AY394991.1_SARS_coronavirus_HZS2-Fc_complete_genome                              95.0%  95.0%     TCAACGAGAAAACACACGTCCAACTCAGT---------------------------------------------------
 60 AY394992.1_SARS_coronavirus_HZS2-C_complete_genome                               95.0%  95.0%     TCAACGAGAAAACACACGTCCAACTCAGT---------------------------------------------------
 61 AY394993.1_SARS_coronavirus_HGZ8L2_complete_genome                               95.0%  95.0%     TCAACGAGAAAACACACGTCCAACTCAGT---------------------------------------------------
 62 AY394994.1_SARS_coronavirus_HSZ-Bc_complete_genome                               95.0%  95.0%     TCAACGAGAAAACACACGTCCAACTCAGT---------------------------------------------------
 63 AY394995.1_SARS_coronavirus_HSZ-Cc_complete_genome                               95.0%  95.0%     TCAACGAGAAAACACACGTCCAACTCAGT---------------------------------------------------
 64 AY394996.1_SARS_coronavirus_ZS-B_complete_genome                                 95.0%  95.0%     TCAACGAGAAAACACACGTCCAACTCAGT---------------------------------------------------
 65 AY394997.1_SARS_coronavirus_ZS-A_complete_genome                                 95.0%  95.0%     TCAACGAGAAAACACACGTCCAACTCAGT---------------------------------------------------
 66 AY394998.1_SARS_coronavirus_LC1_complete_genome                                  95.0%  95.0%     TCAACGAGAAAACACACGTCCAACTCAGT---------------------------------------------------
 67 AY394999.1_SARS_coronavirus_LC2_complete_genome                                  95.0%  95.0%     TCAACGAGAAAACACACGTCCAACTCAGT---------------------------------------------------
 68 AY395000.1_SARS_coronavirus_LC3_complete_genome                                  95.0%  95.0%     TCAACGAGAAAACACACGTCCAACTCAGT---------------------------------------------------
 69 AY395001.1_SARS_coronavirus_LC4_complete_genome                                  95.0%  95.0%     TCAACGAGAAAACACACGTCCAACTCAGT---------------------------------------------------
 70 AY395002.1_SARS_coronavirus_LC5_complete_genome                                  95.0%  95.0%     TCAACGAGAAAACACACGTCCAACTCAGT---------------------------------------------------
 71 AY395003.1_SARS_coronavirus_ZS-C_complete_genome                                 83.0%  83.0%     TCAACGAGAAAACACACGTCCAACTCAGTTTGCCTGTCCTTCAGGTTAGAGACGTGCTAGTGCGT---------------
 72 AY395004.1_SARS_coronavirus_HZS2-Bb_partial_genome                               84.3%  84.3%     TCAACGAGAAAACACACGTCCAACTCAGTTTGCCTGTCCTTCAGGTTAGAGACGTGCTAGT-------------------
 73 AY427439.1_SARS_coronavirus_AS_complete_genome                                   94.7%  94.7%     TCAACGAGAAAACACACGTCCAACTCAGTT--------------------------------------------------
 74 AY461660.1_SARS_coronavirus_SoD_complete_genome                                  95.0%  94.7%     TCAACGAGAAAACACACGTCCAACTCAGT---------------------------------------------------
 75 AY463059.1_SARS_coronavirus_ShanghaiQXC1_complete_genome                         73.7%  73.7%     TCAACGAGAAAACACACGTCCAACTCAGTTTGCCTGTCCTTCAGGTTAGAGACGTGCTAGTGCGTGGCTTCGGGGACTCT
 76 AY463060.1_SARS_coronavirus_ShanghaiQXC2_complete_genome                         73.7%  73.7%     TCAACGAGAAAACACACGTCCAACTCAGTTTGCCTGTCCTTCAGGTTAGAGACGTGCTAGTGCGTGGCTTCGGGGACTCT
 77 AY485277.1_SARS_coronavirus_Sino1-11_complete_genome                            100.0% 100.0%     TCAACGAGAAAACA------------------------------------------------------------------
 78 AY485278.1_SARS_coronavirus_Sino3-11_complete_genome                            100.0% 100.0%     TCAACGAGAAAACA------------------------------------------------------------------
 79 AY502923.1_SARS_coronavirus_TW10_complete_genome                                100.0% 100.0%     TCAACGAGAAAACA------------------------------------------------------------------
 80 AY502924.1_SARS_coronavirus_TW11_complete_genome                                100.0% 100.0%     TCAACGAGAAAACA------------------------------------------------------------------
 81 AY502925.1_SARS_coronavirus_TW2_complete_genome                                 100.0% 100.0%     TCAACGAGAAAACA------------------------------------------------------------------
 82 AY502926.1_SARS_coronavirus_TW3_complete_genome                                 100.0% 100.0%     TCAACGAGAAAACA------------------------------------------------------------------
 83 AY502927.1_SARS_coronavirus_TW4_complete_genome                                 100.0% 100.0%     TCAACGAGAAAACA------------------------------------------------------------------
 84 AY502928.1_SARS_coronavirus_TW5_complete_genome                                 100.0% 100.0%     TCAACGAGAAAACA------------------------------------------------------------------
 85 AY502929.1_SARS_coronavirus_TW6_complete_genome                                 100.0% 100.0%     TCAACGAGAAAACA------------------------------------------------------------------
 86 AY502930.1_SARS_coronavirus_TW7_complete_genome                                 100.0% 100.0%     TCAACGAGAAAACA------------------------------------------------------------------
 87 AY502931.1_SARS_coronavirus_TW8_complete_genome                                 100.0% 100.0%     TCAACGAGAAAACA------------------------------------------------------------------
 88 AY502932.1_SARS_coronavirus_TW9_complete_genome                                 100.0% 100.0%     TCAACGAGAAAACA------------------------------------------------------------------
 89 AY508724.1_SARS_coronavirus_NS-1_complete_genome                                 94.7%  94.7%     TCAACGAGAAAACACACGTCCAACTCAGTT--------------------------------------------------
 90 AY559081.1_SARS_coronavirus_Sin842_complete_genome                               95.7%  95.3%     TCAACGAGAAAACACACGTCCAACTCA-----------------------------------------------------
 91 AY559082.1_SARS_coronavirus_Sin852_complete_genome                               99.7%  96.3%     TCAACGAGAAAACAC-----------------------------------------------------------------
 92 AY559083.1_SARS_coronavirus_Sin3408_complete_genome                              88.7%  85.3%     --------------------------------------------------------------------------------
 93 AY559084.1_SARS_coronavirus_Sin3765V_complete_genome                             94.7%  94.7%     TCAACGAGAAAACACACGTCCAACTCAGTT--------------------------------------------------
 94 AY559085.1_SARS_coronavirus_Sin848_complete_genome                               94.7%  94.7%     TCAACGAGAAAACACACGTCCAACTCAGTT--------------------------------------------------
 95 AY559086.1_SARS_coronavirus_Sin849_complete_genome                               94.7%  94.7%     TCAACGAGAAAACACACGTCCAACTCAGTT--------------------------------------------------
 96 AY559087.1_SARS_coronavirus_Sin3725V_complete_genome                             94.7%  94.7%     TCAACGAGAAAACACACGTCCAACTCAGTT--------------------------------------------------
 97 AY559088.1_SARS_coronavirus_SinP1_complete_genome                                94.7%  94.7%     TCAACGAGAAAACACACGTCCAACTCAGTT--------------------------------------------------
 98 AY559089.1_SARS_coronavirus_SinP2_complete_genome                                94.7%  94.7%     TCAACGAGAAAACACACGTCCAACTCAGTT--------------------------------------------------
 99 AY559090.1_SARS_coronavirus_SinP3_complete_genome                                95.3%  92.7%     TCAACGAGAAAACACACGTCCAACTCAG----------------------------------------------------
100 AY559091.1_SARS_coronavirus_SinP4_complete_genome                                94.7%  94.7%     TCAACGAGAAAACACACGTCCAACTCAGTT--------------------------------------------------
101 AY559092.1_SARS_coronavirus_SinP5_complete_genome                                94.7%  94.7%     TCAACGAGAAAACACACGTCCAACTCAGTT--------------------------------------------------
102 AY559093.1_SARS_coronavirus_Sin845_complete_genome                               94.7%  94.7%     TCAACGAGAAAACACACGTCCAACTCAGTT--------------------------------------------------
103 AY559094.1_SARS_coronavirus_Sin846_complete_genome                               94.7%  94.7%     TCAACGAGAAAACACACGTCCAACTCAGTT--------------------------------------------------
104 AY559095.1_SARS_coronavirus_Sin847_complete_genome                               96.7%  96.0%     TCAACGAGAAAACACACGTCCAAC--------------------------------------------------------
105 AY559096.1_SARS_coronavirus_Sin850_complete_genome                               97.3%  95.7%     TCAACGAGAAAACACACGTCCA----------------------------------------------------------
106 AY559097.1_SARS_coronavirus_Sin3408L_complete_genome                             94.7%  94.7%     TCAACGAGAAAACACACGTCCAACTCAGTT--------------------------------------------------
107 AY595412.1_SARS_coronavirus_LLJ-2004_complete_genome                             93.0%  93.0%     TCAACGAGAAAACACACGTCCAACTCAGTTTGCCT---------------------------------------------
108 AY654624.1_SARS_coronavirus_TJF_complete_genome                                  99.7%  99.0%     TCAACGAGAAAACAC-----------------------------------------------------------------
109 AY714217.1_SARS_Coronavirus_CDC*200301157_complete_genome                       100.0% 100.0%     TCAACGAGAAAACA------------------------------------------------------------------
110 CQ903009.1_Sequence_13_from_Patent_WO2004094667                                 100.0%  99.7%     TCAACGAGAAAACA------------------------------------------------------------------
111 CQ918584.1_Sequence_1_from_Patent_WO2004096842                                   95.0%  95.0%     TCAACGAGAAAACACACGTCCAACTCAGT---------------------------------------------------
112 CQ918585.1_Sequence_2_from_Patent_WO2004096842                                   95.0%  95.0%     TCAACGAGAAAACACACGTCCAACTCAGT---------------------------------------------------
113 CQ918598.1_Sequence_15_from_Patent_WO2004096842                                 100.0% 100.0%     TCAACGAGAAAACA------------------------------------------------------------------
114 NC_004718.3_SARS_coronavirus_complete_genome                                    100.0% 100.0%     TCAACGAGAAAACA------------------------------------------------------------------
    consensus/100%                                                                                 ................................................................................
    consensus/90%                                                                                  TCAACGAGAAAACA..................................................................
    consensus/80%                                                                                  TCAACGAGAAAACA..................................................................
    consensus/70%                                                                                  TCAACGAGAAAACA..................................................................

                                                                                     cov    pid 401         .         .         .         .         :         .         .         . 480
  1 AP006557.1_SARS_coronavirus_TWH_genomic_RNA_complete_genome                      100.0% 100.0%     --------------------------------------------------------------------------------
  2 AP006558.1_SARS_coronavirus_TWJ_genomic_RNA_complete_genome                      100.0% 100.0%     --------------------------------------------------------------------------------
  3 AP006559.1_SARS_coronavirus_TWK_genomic_RNA_complete_genome                      100.0% 100.0%     --------------------------------------------------------------------------------
  4 AP006560.1_SARS_coronavirus_TWS_genomic_RNA_complete_genome                      100.0% 100.0%     --------------------------------------------------------------------------------
  5 AP006561.1_SARS_coronavirus_TWY_genomic_RNA_complete_genome                      100.0% 100.0%     --------------------------------------------------------------------------------
  6 AY274119.3_Severe_acute_respiratory_syndrome-related_coronavirus_isolate_Tor2_complete_genome 100.0% 100.0%     --------------------------------------------------------------------------------
  7 AY278487.3_SARS_coronavirus_BJ02_complete_genome                                100.0% 100.0%     --------------------------------------------------------------------------------
  8 AY278488.2_SARS_coronavirus_BJ01_complete_genome                                 93.7%  93.7%     --------------------------------------------------------------------------------
  9 AY278489.2_SARS_coronavirus_GD01_complete_genome                                 94.7%  94.7%     --------------------------------------------------------------------------------
 10 AY278490.3_SARS_coronavirus_BJ03_complete_genome                                 98.7%  98.7%     --------------------------------------------------------------------------------
 11 AY278491.2_SARS_coronavirus_HKU-39849_complete_genome                           100.0% 100.0%     --------------------------------------------------------------------------------
 12 AY278554.2_SARS_coronavirus_CUHK-W1_complete_genome                              95.0%  95.0%     --------------------------------------------------------------------------------
 13 AY278741.1_SARS_coronavirus_Urbani_complete_genome                              100.0% 100.0%     --------------------------------------------------------------------------------
 14 AY279354.2_SARS_coronavirus_BJ04_complete_genome                                 94.7%  94.7%     --------------------------------------------------------------------------------
 15 AY282752.2_SARS_coronavirus_CUHK-Su10_complete_genome                            95.0%  95.0%     --------------------------------------------------------------------------------
 16 AY283794.1_SARS_coronavirus_Sin2500_complete_genome                              94.7%  94.7%     --------------------------------------------------------------------------------
 17 AY283795.1_SARS_coronavirus_Sin2677_complete_genome                              94.7%  94.7%     --------------------------------------------------------------------------------
 18 AY283796.1_SARS_coronavirus_Sin2679_complete_genome                              94.7%  94.7%     --------------------------------------------------------------------------------
 19 AY283797.1_SARS_coronavirus_Sin2748_complete_genome                              94.7%  94.7%     --------------------------------------------------------------------------------
 20 AY283798.2_SARS_coronavirus_Sin2774_complete_genome                              94.7%  94.7%     --------------------------------------------------------------------------------
 21 AY291315.1_SARS_coronavirus_Frankfurt_1_complete_genome                         100.0% 100.0%     --------------------------------------------------------------------------------
 22 AY291451.1_SARS_coronavirus_TW1_complete_genome                                 100.0% 100.0%     --------------------------------------------------------------------------------
 23 AY297028.1_SARS_coronavirus_ZJ01_complete_genome                                 95.3%  95.3%     --------------------------------------------------------------------------------
 24 AY304486.1_SARS_coronavirus_SZ3_complete_genome                                  95.0%  95.0%     --------------------------------------------------------------------------------
 25 AY304488.1_SARS_coronavirus_SZ16_complete_genome                                 95.0%  95.0%     --------------------------------------------------------------------------------
 26 AY304495.1_SARS_coronavirus_GZ50_complete_genome                                 95.0%  95.0%     --------------------------------------------------------------------------------
 27 AY310120.1_SARS_coronavirus_FRA_complete_genome                                 100.0% 100.0%     --------------------------------------------------------------------------------
 28 AY313906.1_SARS_coronavirus_GD69_complete_genome                                100.0% 100.0%     --------------------------------------------------------------------------------
 29 AY321118.1_SARS_coronavirus_TWC_complete_genome                                 100.0% 100.0%     --------------------------------------------------------------------------------
```

```
 30 AY323977.2_SARS_coronavirus_HSR_1_complete_genome                               100.0% 100.0%     --------------------------------------------------------------------------------
 31 AY338174.1_SARS_coronavirus_Taiwan_TC1_complete_genome                           77.0%  77.0%     GTG-----------------------------------------------------------------------------
 32 AY338175.1_SARS_coronavirus_Taiwan_TC2_complete_genome                           77.0%  77.0%     GTG-----------------------------------------------------------------------------
 33 AY345986.1_SARS_coronavirus_CUHK-AG01_complete_genome                            95.0%  95.0%     --------------------------------------------------------------------------------
 34 AY345987.1_SARS_coronavirus_CUHK-AG02_complete_genome                            95.0%  95.0%     --------------------------------------------------------------------------------
 35 AY345988.1_SARS_coronavirus_CUHK-AG03_complete_genome                            95.0%  95.0%     --------------------------------------------------------------------------------
 36 AY348314.1_SARS_coronavirus_Taiwan_TC3_complete_genome                           77.0%  77.0%     GTG-----------------------------------------------------------------------------
 37 AY350750.1_SARS_coronavirus_PUMC01_complete_genome                               95.7%  95.7%     --------------------------------------------------------------------------------
 38 AY351680.1_SARS_coronavirus_ZMY_1_complete_genome                               100.0%  99.7%     --------------------------------------------------------------------------------
 39 AY357075.1_SARS_coronavirus_PUMC02_complete_genome                               95.3%  95.3%     --------------------------------------------------------------------------------
 40 AY357076.1_SARS_coronavirus_PUMC03_complete_genome                               95.3%  95.3%     --------------------------------------------------------------------------------
 41 AY362698.1_SARS_coronavirus_TWC2_complete_genome                                100.0% 100.0%     --------------------------------------------------------------------------------
 42 AY362699.1_SARS_coronavirus_TWC3_complete_genome                                100.0% 100.0%     --------------------------------------------------------------------------------
 43 AY390556.1_SARS_coronavirus_GZ02_complete_genome                                100.0% 100.0%     --------------------------------------------------------------------------------
 44 AY394850.2_SARS_coronavirus_WHU_complete_genome                                 100.0% 100.0%     --------------------------------------------------------------------------------
 45 AY394977.1_SARS_coronavirus_GZ-A_partial_genome                                  83.0%  83.0%     --------------------------------------------------------------------------------
 46 AY394978.1_SARS_coronavirus_GZ-B_complete_genome                                 76.0%  76.0%     GTGGAA--------------------------------------------------------------------------
 47 AY394979.1_SARS_coronavirus_GZ-C_complete_genome                                 82.7%  82.7%     --------------------------------------------------------------------------------
 48 AY394980.1_SARS_coronavirus_GZ-D_partial_genome                                  84.7%  84.3%     --------------------------------------------------------------------------------
 49 AY394981.1_SARS_coronavirus_HGZ8L1-A_partial_genome                              83.0%  83.0%     --------------------------------------------------------------------------------
 50 AY394982.1_SARS_coronavirus_HGZ8L1-B_partial_genome                              82.7%  82.7%     --------------------------------------------------------------------------------
 51 AY394983.1_SARS_coronavirus_HSZ2-A_complete_genome                               82.7%  82.7%     --------------------------------------------------------------------------------
 52 AY394984.1_SARS_coronavirus_HSZ-A_partial_genome                                 84.3%  84.3%     --------------------------------------------------------------------------------
 53 AY394985.1_SARS_coronavirus_HSZ-Bb_complete_genome                               16.7%  16.7%     GTGGAAGAGGCCCTATCGGAGGCACGTGAACACCTCAAAAATGGCACTTGTGGTCTAGTAGAGCTGGAAAAAGGCGTACT
 54 AY394986.1_SARS_coronavirus_HSZ-Cb_complete_genome                               83.0%  83.0%     --------------------------------------------------------------------------------
 55 AY394987.1_SARS_coronavirus_HZS2-Fb_complete_genome                              86.0%  86.0%     --------------------------------------------------------------------------------
 56 AY394988.1_SARS_coronavirus_JMD_partial_genome                                   71.3%  71.0%     GTGGAAGAGGCCCTATCGGA------------------------------------------------------------
 57 AY394989.1_SARS_coronavirus_HZS2-D_complete_genome                               95.0%  95.0%     --------------------------------------------------------------------------------
 58 AY394990.1_SARS_coronavirus_HZS2-E_complete_genome                               95.0%  95.0%     --------------------------------------------------------------------------------
 59 AY394991.1_SARS_coronavirus_HZS2-Fc_complete_genome                              95.0%  95.0%     --------------------------------------------------------------------------------
 60 AY394992.1_SARS_coronavirus_HZS2-C_complete_genome                               95.0%  95.0%     --------------------------------------------------------------------------------
 61 AY394993.1_SARS_coronavirus_HGZ8L2_complete_genome                               95.0%  95.0%     --------------------------------------------------------------------------------
 62 AY394994.1_SARS_coronavirus_HSZ-Bc_complete_genome                               95.0%  95.0%     --------------------------------------------------------------------------------
 63 AY394995.1_SARS_coronavirus_HSZ-Cc_complete_genome                               95.0%  95.0%     --------------------------------------------------------------------------------
 64 AY394996.1_SARS_coronavirus_ZS-B_complete_genome                                 95.0%  95.0%     --------------------------------------------------------------------------------
 65 AY394997.1_SARS_coronavirus_ZS-A_complete_genome                                 95.0%  95.0%     --------------------------------------------------------------------------------
 66 AY394998.1_SARS_coronavirus_LC1_complete_genome                                  95.0%  95.0%     --------------------------------------------------------------------------------
 67 AY394999.1_SARS_coronavirus_LC2_complete_genome                                  95.0%  95.0%     --------------------------------------------------------------------------------
 68 AY395000.1_SARS_coronavirus_LC3_complete_genome                                  95.0%  95.0%     --------------------------------------------------------------------------------
 69 AY395001.1_SARS_coronavirus_LC4_complete_genome                                  95.0%  95.0%     --------------------------------------------------------------------------------
 70 AY395002.1_SARS_coronavirus_LC5_complete_genome                                  95.0%  95.0%     --------------------------------------------------------------------------------
 71 AY395003.1_SARS_coronavirus_ZS-C_complete_genome                                 83.0%  83.0%     --------------------------------------------------------------------------------
 72 AY395004.1_SARS_coronavirus_HZS2-Bb_partial_genome                               84.3%  84.3%     --------------------------------------------------------------------------------
 73 AY427439.1_SARS_coronavirus_AS_complete_genome                                   94.7%  94.7%     --------------------------------------------------------------------------------
 74 AY461660.1_SARS_coronavirus_SoD_complete_genome                                  95.0%  94.7%     --------------------------------------------------------------------------------
 75 AY463059.1_SARS_coronavirus_ShanghaiQXC1_complete_genome                         73.7%  73.7%     GTGGAAGAGGCCC-------------------------------------------------------------------
 76 AY463060.1_SARS_coronavirus_ShanghaiQXC2_complete_genome                         73.7%  73.7%     GTGGAAGAGGCCC-------------------------------------------------------------------
 77 AY485277.1_SARS_coronavirus_Sino1-11_complete_genome                            100.0% 100.0%     --------------------------------------------------------------------------------
 78 AY485278.1_SARS_coronavirus_Sino3-11_complete_genome                            100.0% 100.0%     --------------------------------------------------------------------------------
 79 AY502923.1_SARS_coronavirus_TW10_complete_genome                                100.0% 100.0%     --------------------------------------------------------------------------------
 80 AY502924.1_SARS_coronavirus_TW11_complete_genome                                100.0% 100.0%     --------------------------------------------------------------------------------
 81 AY502925.1_SARS_coronavirus_TW2_complete_genome                                 100.0% 100.0%     --------------------------------------------------------------------------------
 82 AY502926.1_SARS_coronavirus_TW3_complete_genome                                 100.0% 100.0%     --------------------------------------------------------------------------------
 83 AY502927.1_SARS_coronavirus_TW4_complete_genome                                 100.0% 100.0%     --------------------------------------------------------------------------------
 84 AY502928.1_SARS_coronavirus_TW5_complete_genome                                 100.0% 100.0%     --------------------------------------------------------------------------------
 85 AY502929.1_SARS_coronavirus_TW6_complete_genome                                 100.0% 100.0%     --------------------------------------------------------------------------------
 86 AY502930.1_SARS_coronavirus_TW7_complete_genome                                 100.0% 100.0%     --------------------------------------------------------------------------------
 87 AY502931.1_SARS_coronavirus_TW8_complete_genome                                 100.0% 100.0%     --------------------------------------------------------------------------------
 88 AY502932.1_SARS_coronavirus_TW9_complete_genome                                 100.0% 100.0%     --------------------------------------------------------------------------------
 89 AY508724.1_SARS_coronavirus_NS-1_complete_genome                                 94.7%  94.7%     --------------------------------------------------------------------------------
 90 AY559081.1_SARS_coronavirus_Sin842_complete_genome                               95.7%  95.3%     --------------------------------------------------------------------------------
 91 AY559082.1_SARS_coronavirus_Sin852_complete_genome                               99.7%  96.3%     --------------------------------------------------------------------------------
 92 AY559083.1_SARS_coronavirus_Sin3408_complete_genome                              88.7%  85.3%     --------------------------------------------------------------------------------
 93 AY559084.1_SARS_coronavirus_Sin3765V_complete_genome                             94.7%  94.7%     --------------------------------------------------------------------------------
 94 AY559085.1_SARS_coronavirus_Sin848_complete_genome                               94.7%  94.7%     --------------------------------------------------------------------------------
 95 AY559086.1_SARS_coronavirus_Sin849_complete_genome                               94.7%  94.7%     --------------------------------------------------------------------------------
 96 AY559087.1_SARS_coronavirus_Sin3725V_complete_genome                             94.7%  94.7%     --------------------------------------------------------------------------------
 97 AY559088.1_SARS_coronavirus_SinP1_complete_genome                                94.7%  94.7%     --------------------------------------------------------------------------------
 98 AY559089.1_SARS_coronavirus_SinP2_complete_genome                                94.7%  94.7%     --------------------------------------------------------------------------------
 99 AY559090.1_SARS_coronavirus_SinP3_complete_genome                                95.3%  92.7%     --------------------------------------------------------------------------------
100 AY559091.1_SARS_coronavirus_SinP4_complete_genome                                94.7%  94.7%     --------------------------------------------------------------------------------
101 AY559092.1_SARS_coronavirus_SinP5_complete_genome                                94.7%  94.7%     --------------------------------------------------------------------------------
102 AY559093.1_SARS_coronavirus_Sin845_complete_genome                               94.7%  94.7%     --------------------------------------------------------------------------------
103 AY559094.1_SARS_coronavirus_Sin846_complete_genome                               94.7%  94.7%     --------------------------------------------------------------------------------
104 AY559095.1_SARS_coronavirus_Sin847_complete_genome                               96.7%  96.0%     --------------------------------------------------------------------------------
105 AY559096.1_SARS_coronavirus_Sin850_complete_genome                               97.3%  95.7%     --------------------------------------------------------------------------------
106 AY559097.1_SARS_coronavirus_Sin3408L_complete_genome                             94.7%  94.7%     --------------------------------------------------------------------------------
107 AY595412.1_SARS_coronavirus_LLJ-2004_complete_genome                             93.0%  93.0%     --------------------------------------------------------------------------------
108 AY654624.1_SARS_coronavirus_TJF_complete_genome                                  99.7%  99.0%     --------------------------------------------------------------------------------
109 AY714217.1_SARS_Coronavirus_CDC*200301157_complete_genome                       100.0% 100.0%     --------------------------------------------------------------------------------
110 CQ903009.1_Sequence_13_from_Patent_WO2004094667                                 100.0%  99.7%     --------------------------------------------------------------------------------
111 CQ918584.1_Sequence_1_from_Patent_WO2004096842                                   95.0%  95.0%     --------------------------------------------------------------------------------
112 CQ918585.1_Sequence_2_from_Patent_WO2004096842                                   95.0%  95.0%     --------------------------------------------------------------------------------
113 CQ918598.1_Sequence_15_from_Patent_WO2004096842                                 100.0% 100.0%     --------------------------------------------------------------------------------
114 NC_004718.3_SARS_coronavirus_complete_genome                                    100.0% 100.0%     --------------------------------------------------------------------------------
    consensus/100%                                                                                    ................................................................................
    consensus/90%                                                                                     ................................................................................
    consensus/80%                                                                                     ................................................................................
    consensus/70%                                                                                     ................................................................................

                                                                                       cov    pid 481        .         5         .         .         .         .         :         . 560
  1 AP006557.1_SARS_coronavirus_TWH_genomic_RNA_complete_genome                     100.0% 100.0%     --------------------------------------------------------------------------------
  2 AP006558.1_SARS_coronavirus_TWJ_genomic_RNA_complete_genome                     100.0% 100.0%     --------------------------------------------------------------------------------
  3 AP006559.1_SARS_coronavirus_TWK_genomic_RNA_complete_genome                     100.0% 100.0%     --------------------------------------------------------------------------------
  4 AP006560.1_SARS_coronavirus_TWS_genomic_RNA_complete_genome                     100.0% 100.0%     --------------------------------------------------------------------------------
  5 AP006561.1_SARS_coronavirus_TWY_genomic_RNA_complete_genome                     100.0% 100.0%     --------------------------------------------------------------------------------
  6 AY274119.3_Severe_acute_respiratory_syndrome-related_coronavirus_isolate_Tor2_complete_genome 100.0% 100.0%     --------------------------------------------------------------------------------
  7 AY278487.3_SARS_coronavirus_BJ02_complete_genome                                100.0% 100.0%     --------------------------------------------------------------------------------
  8 AY278488.2_SARS_coronavirus_BJ01_complete_genome                                 93.7%  93.7%     --------------------------------------------------------------------------------
  9 AY278489.2_SARS_coronavirus_GD01_complete_genome                                 94.7%  94.7%     --------------------------------------------------------------------------------
 10 AY278490.3_SARS_coronavirus_BJ03_complete_genome                                 98.7%  98.7%     --------------------------------------------------------------------------------
 11 AY278491.2_SARS_coronavirus_HKU-39849_complete_genome                           100.0% 100.0%     --------------------------------------------------------------------------------
 12 AY278554.2_SARS_coronavirus_CUHK-W1_complete_genome                              95.0%  95.0%     --------------------------------------------------------------------------------
 13 AY278741.1_SARS_coronavirus_Urbani_complete_genome                              100.0% 100.0%     --------------------------------------------------------------------------------
 14 AY279354.2_SARS_coronavirus_BJ04_complete_genome                                 94.7%  94.7%     --------------------------------------------------------------------------------
 15 AY282752.2_SARS_coronavirus_CUHK-Su10_complete_genome                            95.0%  95.0%     --------------------------------------------------------------------------------
 16 AY283794.1_SARS_coronavirus_Sin2500_complete_genome                              94.7%  94.7%     --------------------------------------------------------------------------------
 17 AY283795.1_SARS_coronavirus_Sin2677_complete_genome                              94.7%  94.7%     --------------------------------------------------------------------------------
 18 AY283796.1_SARS_coronavirus_Sin2679_complete_genome                              94.7%  94.7%     --------------------------------------------------------------------------------
 19 AY283797.1_SARS_coronavirus_Sin2748_complete_genome                              94.7%  94.7%     --------------------------------------------------------------------------------
 20 AY283798.2_SARS_coronavirus_Sin2774_complete_genome                              94.7%  94.7%     --------------------------------------------------------------------------------
 21 AY291315.1_SARS_coronavirus_Frankfurt_1_complete_genome                         100.0% 100.0%     --------------------------------------------------------------------------------
 22 AY291451.1_SARS_coronavirus_TW1_complete_genome                                 100.0% 100.0%     --------------------------------------------------------------------------------
 23 AY297028.1_SARS_coronavirus_ZJ01_complete_genome                                 95.3%  95.3%     --------------------------------------------------------------------------------
 24 AY304486.1_SARS_coronavirus_SZ3_complete_genome                                  95.0%  95.0%     --------------------------------------------------------------------------------
 25 AY304488.1_SARS_coronavirus_SZ16_complete_genome                                 95.0%  95.0%     --------------------------------------------------------------------------------
 26 AY304495.1_SARS_coronavirus_GZ50_complete_genome                                 95.0%  95.0%     --------------------------------------------------------------------------------
 27 AY310120.1_SARS_coronavirus_FRA_complete_genome                                 100.0% 100.0%     --------------------------------------------------------------------------------
 28 AY313906.1_SARS_coronavirus_GD69_complete_genome                                100.0% 100.0%     --------------------------------------------------------------------------------
 29 AY321118.1_SARS_coronavirus_TWC_complete_genome                                 100.0% 100.0%     --------------------------------------------------------------------------------
 30 AY323977.2_SARS_coronavirus_HSR_1_complete_genome                               100.0% 100.0%     --------------------------------------------------------------------------------
 31 AY338174.1_SARS_coronavirus_Taiwan_TC1_complete_genome                           77.0%  77.0%     --------------------------------------------------------------------------------
 32 AY338175.1_SARS_coronavirus_Taiwan_TC2_complete_genome                           77.0%  77.0%     --------------------------------------------------------------------------------
 33 AY345986.1_SARS_coronavirus_CUHK-AG01_complete_genome                            95.0%  95.0%     --------------------------------------------------------------------------------
 34 AY345987.1_SARS_coronavirus_CUHK-AG02_complete_genome                            95.0%  95.0%     --------------------------------------------------------------------------------
 35 AY345988.1_SARS_coronavirus_CUHK-AG03_complete_genome                            95.0%  95.0%     --------------------------------------------------------------------------------
 36 AY348314.1_SARS_coronavirus_Taiwan_TC3_complete_genome                           77.0%  77.0%     --------------------------------------------------------------------------------
 37 AY350750.1_SARS_coronavirus_PUMC01_complete_genome                               95.7%  95.7%     --------------------------------------------------------------------------------
 38 AY351680.1_SARS_coronavirus_ZMY_1_complete_genome                               100.0%  99.7%     --------------------------------------------------------------------------------
 39 AY357075.1_SARS_coronavirus_PUMC02_complete_genome                               95.3%  95.3%     --------------------------------------------------------------------------------
 40 AY357076.1_SARS_coronavirus_PUMC03_complete_genome                               95.3%  95.3%     --------------------------------------------------------------------------------
 41 AY362698.1_SARS_coronavirus_TWC2_complete_genome                                100.0% 100.0%     --------------------------------------------------------------------------------
 42 AY362699.1_SARS_coronavirus_TWC3_complete_genome                                100.0% 100.0%     --------------------------------------------------------------------------------
 43 AY390556.1_SARS_coronavirus_GZ02_complete_genome                                100.0% 100.0%     --------------------------------------------------------------------------------
 44 AY394850.2_SARS_coronavirus_WHU_complete_genome                                 100.0% 100.0%     --------------------------------------------------------------------------------
 45 AY394977.1_SARS_coronavirus_GZ-A_partial_genome                                  83.0%  83.0%     --------------------------------------------------------------------------------
 46 AY394978.1_SARS_coronavirus_GZ-B_complete_genome                                 76.0%  76.0%     --------------------------------------------------------------------------------
 47 AY394979.1_SARS_coronavirus_GZ-C_complete_genome                                 82.7%  82.7%     --------------------------------------------------------------------------------
 48 AY394980.1_SARS_coronavirus_GZ-D_partial_genome                                  84.7%  84.3%     --------------------------------------------------------------------------------
 49 AY394981.1_SARS_coronavirus_HGZ8L1-A_partial_genome                              83.0%  83.0%     --------------------------------------------------------------------------------
 50 AY394982.1_SARS_coronavirus_HGZ8L1-B_partial_genome                              82.7%  82.7%     --------------------------------------------------------------------------------
 51 AY394983.1_SARS_coronavirus_HSZ2-A_complete_genome                               82.7%  82.7%     --------------------------------------------------------------------------------
 52 AY394984.1_SARS_coronavirus_HSZ-A_partial_genome                                 84.3%  84.3%     --------------------------------------------------------------------------------
 53 AY394985.1_SARS_coronavirus_HSZ-Bb_complete_genome                               16.7%  16.7%     GCCCCAGCTTGAACAGCCCTATGTGTTCATTAAACGTTCTGATGCCTTAAGCACCAATCACGGCCACAAGGTCGTTGAGC
 54 AY394986.1_SARS_coronavirus_HSZ-Cb_complete_genome                               83.0%  83.0%     --------------------------------------------------------------------------------
 55 AY394987.1_SARS_coronavirus_HZS2-Fb_complete_genome                              86.0%  86.0%     --------------------------------------------------------------------------------
 56 AY394988.1_SARS_coronavirus_JMD_partial_genome                                   71.3%  71.0%     --------------------------------------------------------------------------------
 57 AY394989.1_SARS_coronavirus_HZS2-D_complete_genome                               95.0%  95.0%     --------------------------------------------------------------------------------
 58 AY394990.1_SARS_coronavirus_HZS2-E_complete_genome                               95.0%  95.0%     --------------------------------------------------------------------------------
 59 AY394991.1_SARS_coronavirus_HZS2-Fc_complete_genome                              95.0%  95.0%     --------------------------------------------------------------------------------
 60 AY394992.1_SARS_coronavirus_HZS2-C_complete_genome                               95.0%  95.0%     --------------------------------------------------------------------------------
 61 AY394993.1_SARS_coronavirus_HGZ8L2_complete_genome                               95.0%  95.0%     --------------------------------------------------------------------------------
 62 AY394994.1_SARS_coronavirus_HSZ-Bc_complete_genome                               95.0%  95.0%     --------------------------------------------------------------------------------
 63 AY394995.1_SARS_coronavirus_HSZ-Cc_complete_genome                               95.0%  95.0%     --------------------------------------------------------------------------------
 64 AY394996.1_SARS_coronavirus_ZS-B_complete_genome                                 95.0%  95.0%     --------------------------------------------------------------------------------
 65 AY394997.1_SARS_coronavirus_ZS-A_complete_genome                                 95.0%  95.0%     --------------------------------------------------------------------------------
 66 AY394998.1_SARS_coronavirus_LC1_complete_genome                                  95.0%  95.0%     --------------------------------------------------------------------------------
 67 AY394999.1_SARS_coronavirus_LC2_complete_genome                                  95.0%  95.0%     --------------------------------------------------------------------------------
 68 AY395000.1_SARS_coronavirus_LC3_complete_genome                                  95.0%  95.0%     --------------------------------------------------------------------------------
```

```
 69 AY395001.1_SARS_coronavirus_LC4_complete_genome                 95.0%  95.0%     --------------------------------------------------------------------------------
 70 AY395002.1_SARS_coronavirus_LC5_complete_genome                 95.0%  95.0%     --------------------------------------------------------------------------------
 71 AY395003.1_SARS_coronavirus_ZS-C_complete_genome                83.0%  83.0%     --------------------------------------------------------------------------------
 72 AY395004.1_SARS_coronavirus_HZS2-Bb_partial_genome              84.3%  84.3%     --------------------------------------------------------------------------------
 73 AY427439.1_SARS_coronavirus_AS_complete_genome                  94.7%  94.7%     --------------------------------------------------------------------------------
 74 AY461660.1_SARS_coronavirus_SoD_complete_genome                 95.0%  94.7%     --------------------------------------------------------------------------------
 75 AY463059.1_SARS_coronavirus_ShanghaiQXC1_complete_genome        73.7%  73.7%     --------------------------------------------------------------------------------
 76 AY463060.1_SARS_coronavirus_ShanghaiQXC2_complete_genome        73.7%  73.7%     --------------------------------------------------------------------------------
 77 AY485277.1_SARS_coronavirus_Sino1-11_complete_genome           100.0% 100.0%     --------------------------------------------------------------------------------
 78 AY485278.1_SARS_coronavirus_Sino3-11_complete_genome           100.0% 100.0%     --------------------------------------------------------------------------------
 79 AY502923.1_SARS_coronavirus_TW10_complete_genome               100.0% 100.0%     --------------------------------------------------------------------------------
 80 AY502924.1_SARS_coronavirus_TW11_complete_genome               100.0% 100.0%     --------------------------------------------------------------------------------
 81 AY502925.1_SARS_coronavirus_TW2_complete_genome                100.0% 100.0%     --------------------------------------------------------------------------------
 82 AY502926.1_SARS_coronavirus_TW3_complete_genome                100.0% 100.0%     --------------------------------------------------------------------------------
 83 AY502927.1_SARS_coronavirus_TW4_complete_genome                100.0% 100.0%     --------------------------------------------------------------------------------
 84 AY502928.1_SARS_coronavirus_TW5_complete_genome                100.0% 100.0%     --------------------------------------------------------------------------------
 85 AY502929.1_SARS_coronavirus_TW6_complete_genome                100.0% 100.0%     --------------------------------------------------------------------------------
 86 AY502930.1_SARS_coronavirus_TW7_complete_genome                100.0% 100.0%     --------------------------------------------------------------------------------
 87 AY502931.1_SARS_coronavirus_TW8_complete_genome                100.0% 100.0%     --------------------------------------------------------------------------------
 88 AY502932.1_SARS_coronavirus_TW9_complete_genome                100.0% 100.0%     --------------------------------------------------------------------------------
 89 AY508724.1_SARS_coronavirus_NS-1_complete_genome                94.7%  94.7%     --------------------------------------------------------------------------------
 90 AY559081.1_SARS_coronavirus_Sin842_complete_genome              95.7%  95.3%     --------------------------------------------------------------------------------
 91 AY559082.1_SARS_coronavirus_Sin852_complete_genome              99.7%  96.3%     --------------------------------------------------------------------------------
 92 AY559083.1_SARS_coronavirus_Sin3408_complete_genome             88.7%  85.3%     --------------------------------------------------------------------------------
 93 AY559084.1_SARS_coronavirus_Sin3765V_complete_genome            94.7%  94.7%     --------------------------------------------------------------------------------
 94 AY559085.1_SARS_coronavirus_Sin848_complete_genome              94.7%  94.7%     --------------------------------------------------------------------------------
 95 AY559086.1_SARS_coronavirus_Sin849_complete_genome              94.7%  94.7%     --------------------------------------------------------------------------------
 96 AY559087.1_SARS_coronavirus_Sin3725V_complete_genome            94.7%  94.7%     --------------------------------------------------------------------------------
 97 AY559088.1_SARS_coronavirus_SinP1_complete_genome               94.7%  94.7%     --------------------------------------------------------------------------------
 98 AY559089.1_SARS_coronavirus_SinP2_complete_genome               94.7%  94.7%     --------------------------------------------------------------------------------
 99 AY559090.1_SARS_coronavirus_SinP3_complete_genome               95.3%  92.7%     --------------------------------------------------------------------------------
100 AY559091.1_SARS_coronavirus_SinP4_complete_genome               94.7%  94.7%     --------------------------------------------------------------------------------
101 AY559092.1_SARS_coronavirus_SinP5_complete_genome               94.7%  94.7%     --------------------------------------------------------------------------------
102 AY559093.1_SARS_coronavirus_Sin845_complete_genome              94.7%  94.7%     --------------------------------------------------------------------------------
103 AY559094.1_SARS_coronavirus_Sin846_complete_genome              94.7%  94.7%     --------------------------------------------------------------------------------
104 AY559095.1_SARS_coronavirus_Sin847_complete_genome              96.7%  96.0%     --------------------------------------------------------------------------------
105 AY559096.1_SARS_coronavirus_Sin850_complete_genome              97.3%  95.7%     --------------------------------------------------------------------------------
106 AY559097.1_SARS_coronavirus_Sin3408L_complete_genome            94.7%  94.7%     --------------------------------------------------------------------------------
107 AY595412.1_SARS_coronavirus_LLJ-2004_complete_genome            93.0%  93.0%     --------------------------------------------------------------------------------
108 AY654624.1_SARS_coronavirus_TJF_complete_genome                 99.7%  99.0%     --------------------------------------------------------------------------------
109 AY714217.1_SARS_Coronavirus_CDC*200301157_complete_genome      100.0% 100.0%     --------------------------------------------------------------------------------
110 CQ903009.1_Sequence_13_from_Patent_WO2004094667                100.0%  99.7%     --------------------------------------------------------------------------------
111 CQ918584.1_Sequence_1_from_Patent_WO2004096842                  95.0%  95.0%     --------------------------------------------------------------------------------
112 CQ918585.1_Sequence_2_from_Patent_WO2004096842                  95.0%  95.0%     --------------------------------------------------------------------------------
113 CQ918598.1_Sequence_15_from_Patent_WO2004096842                100.0% 100.0%     --------------------------------------------------------------------------------
114 NC_004718.3_SARS_coronavirus_complete_genome                   100.0% 100.0%     --------------------------------------------------------------------------------
    consensus/100%                                                                   ................................................................................
    consensus/90%                                                                    ................................................................................
    consensus/80%                                                                    ................................................................................
    consensus/70%                                                                    ................................................................................

                                                                      cov    pid 561         .         .  ] 584
  1 AP006557.1_SARS_coronavirus_TWH_genomic_RNA_complete_genome    100.0% 100.0%     ------------------------
  2 AP006558.1_SARS_coronavirus_TWJ_genomic_RNA_complete_genome    100.0% 100.0%     ------------------------
  3 AP006559.1_SARS_coronavirus_TWK_genomic_RNA_complete_genome    100.0% 100.0%     ------------------------
  4 AP006560.1_SARS_coronavirus_TWS_genomic_RNA_complete_genome    100.0% 100.0%     ------------------------
  5 AP006561.1_SARS_coronavirus_TWY_genomic_RNA_complete_genome    100.0% 100.0%     ------------------------
  6 AY274119.3_Severe_acute_respiratory_syndrome-related_coronavirus_isolate_Tor2_complete_genome 100.0% 100.0%     ------------------------
  7 AY278487.3_SARS_coronavirus_BJ02_complete_genome               100.0% 100.0%     ------------------------
  8 AY278488.2_SARS_coronavirus_BJ01_complete_genome                93.7%  93.7%     ------------------------
  9 AY278489.2_SARS_coronavirus_GD01_complete_genome                94.7%  94.7%     ------------------------
 10 AY278490.3_SARS_coronavirus_BJ03_complete_genome                98.7%  98.7%     ------------------------
 11 AY278491.2_SARS_coronavirus_HKU-39849_complete_genome          100.0% 100.0%     ------------------------
 12 AY278554.2_SARS_coronavirus_CUHK-W1_complete_genome             95.0%  95.0%     ------------------------
 13 AY278741.1_SARS_coronavirus_Urbani_complete_genome             100.0% 100.0%     ------------------------
 14 AY279354.2_SARS_coronavirus_BJ04_complete_genome                94.7%  94.7%     ------------------------
 15 AY282752.2_SARS_coronavirus_CUHK-Su10_complete_genome           95.0%  95.0%     ------------------------
 16 AY283794.1_SARS_coronavirus_Sin2500_complete_genome             94.7%  94.7%     ------------------------
 17 AY283795.1_SARS_coronavirus_Sin2677_complete_genome             94.7%  94.7%     ------------------------
 18 AY283796.1_SARS_coronavirus_Sin2679_complete_genome             94.7%  94.7%     ------------------------
 19 AY283797.1_SARS_coronavirus_Sin2748_complete_genome             94.7%  94.7%     ------------------------
 20 AY283798.2_SARS_coronavirus_Sin2774_complete_genome             94.7%  94.7%     ------------------------
 21 AY291315.1_SARS_coronavirus_Frankfurt_1_complete_genome        100.0% 100.0%     ------------------------
 22 AY291451.1_SARS_coronavirus_TW1_complete_genome                100.0% 100.0%     ------------------------
 23 AY297028.1_SARS_coronavirus_ZJ01_complete_genome                95.3%  95.3%     ------------------------
 24 AY304486.1_SARS_coronavirus_SZ3_complete_genome                 95.0%  95.0%     ------------------------
 25 AY304488.1_SARS_coronavirus_SZ16_complete_genome                95.0%  95.0%     ------------------------
 26 AY304495.1_SARS_coronavirus_GZ50_complete_genome                95.0%  95.0%     ------------------------
 27 AY310120.1_SARS_coronavirus_FRA_complete_genome                100.0% 100.0%     ------------------------
 28 AY313906.1_SARS_coronavirus_GD69_complete_genome               100.0% 100.0%     ------------------------
 29 AY321118.1_SARS_coronavirus_TWC_complete_genome                100.0% 100.0%     ------------------------
 30 AY323977.2_SARS_coronavirus_HSR_1_complete_genome              100.0% 100.0%     ------------------------
 31 AY338174.1_SARS_coronavirus_Taiwan_TC1_complete_genome          77.0%  77.0%     ------------------------
 32 AY338175.1_SARS_coronavirus_Taiwan_TC2_complete_genome          77.0%  77.0%     ------------------------
 33 AY345986.1_SARS_coronavirus_CUHK-AG01_complete_genome           95.0%  95.0%     ------------------------
 34 AY345987.1_SARS_coronavirus_CUHK-AG02_complete_genome           95.0%  95.0%     ------------------------
 35 AY345988.1_SARS_coronavirus_CUHK-AG03_complete_genome           95.0%  95.0%     ------------------------
 36 AY348314.1_SARS_coronavirus_Taiwan_TC3_complete_genome          77.0%  77.0%     ------------------------
 37 AY350750.1_SARS_coronavirus_PUMC01_complete_genome              95.7%  95.7%     ------------------------
 38 AY351680.1_SARS_coronavirus_ZMY_1_complete_genome              100.0%  99.7%     ------------------------
 39 AY357075.1_SARS_coronavirus_PUMC02_complete_genome              95.3%  95.3%     ------------------------
 40 AY357076.1_SARS_coronavirus_PUMC03_complete_genome              95.3%  95.3%     ------------------------
 41 AY362698.1_SARS_coronavirus_TWC2_complete_genome               100.0% 100.0%     ------------------------
 42 AY362699.1_SARS_coronavirus_TWC3_complete_genome               100.0% 100.0%     ------------------------
 43 AY390556.1_SARS_coronavirus_GZ02_complete_genome               100.0% 100.0%     ------------------------
 44 AY394850.2_SARS_coronavirus_WHU_complete_genome                100.0% 100.0%     ------------------------
 45 AY394977.1_SARS_coronavirus_GZ-A_partial_genome                 83.0%  83.0%     ------------------------
 46 AY394978.1_SARS_coronavirus_GZ-B_complete_genome                76.0%  76.0%     ------------------------
 47 AY394979.1_SARS_coronavirus_GZ-C_complete_genome                82.7%  82.7%     ------------------------
 48 AY394980.1_SARS_coronavirus_GZ-D_partial_genome                 84.7%  84.3%     ------------------------
 49 AY394981.1_SARS_coronavirus_HGZ8L1-A_partial_genome             83.0%  83.0%     ------------------------
 50 AY394982.1_SARS_coronavirus_HGZ8L1-B_partial_genome             82.7%  82.7%     ------------------------
 51 AY394983.1_SARS_coronavirus_HSZ2-A_complete_genome              82.7%  82.7%     ------------------------
 52 AY394984.1_SARS_coronavirus_HSZ-A_partial_genome                84.3%  84.3%     ------------------------
 53 AY394985.1_SARS_coronavirus_HSZ-Bb_complete_genome              16.7%  16.7%     TGGTTGCAGAAATGGACGGCATTC
 54 AY394986.1_SARS_coronavirus_HSZ-Cb_complete_genome              83.0%  83.0%     ------------------------
 55 AY394987.1_SARS_coronavirus_HZS2-Fb_complete_genome             86.0%  86.0%     ------------------------
 56 AY394988.1_SARS_coronavirus_JMD_partial_genome                  71.3%  71.0%     ------------------------
 57 AY394989.1_SARS_coronavirus_HZS2-D_complete_genome              95.0%  95.0%     ------------------------
 58 AY394990.1_SARS_coronavirus_HZS2-E_complete_genome              95.0%  95.0%     ------------------------
 59 AY394991.1_SARS_coronavirus_HZS2-Fc_complete_genome             95.0%  95.0%     ------------------------
 60 AY394992.1_SARS_coronavirus_HZS2-C_complete_genome              95.0%  95.0%     ------------------------
 61 AY394993.1_SARS_coronavirus_HGZ8L2_complete_genome              95.0%  95.0%     ------------------------
 62 AY394994.1_SARS_coronavirus_HSZ-Bc_complete_genome              95.0%  95.0%     ------------------------
 63 AY394995.1_SARS_coronavirus_HSZ-Cc_complete_genome              95.0%  95.0%     ------------------------
 64 AY394996.1_SARS_coronavirus_ZS-B_complete_genome                95.0%  95.0%     ------------------------
 65 AY394997.1_SARS_coronavirus_ZS-A_complete_genome                95.0%  95.0%     ------------------------
 66 AY394998.1_SARS_coronavirus_LC1_complete_genome                 95.0%  95.0%     ------------------------
 67 AY394999.1_SARS_coronavirus_LC2_complete_genome                 95.0%  95.0%     ------------------------
 68 AY395000.1_SARS_coronavirus_LC3_complete_genome                 95.0%  95.0%     ------------------------
 69 AY395001.1_SARS_coronavirus_LC4_complete_genome                 95.0%  95.0%     ------------------------
 70 AY395002.1_SARS_coronavirus_LC5_complete_genome                 95.0%  95.0%     ------------------------
 71 AY395003.1_SARS_coronavirus_ZS-C_complete_genome                83.0%  83.0%     ------------------------
 72 AY395004.1_SARS_coronavirus_HZS2-Bb_partial_genome              84.3%  84.3%     ------------------------
 73 AY427439.1_SARS_coronavirus_AS_complete_genome                  94.7%  94.7%     ------------------------
 74 AY461660.1_SARS_coronavirus_SoD_complete_genome                 95.0%  94.7%     ------------------------
 75 AY463059.1_SARS_coronavirus_ShanghaiQXC1_complete_genome        73.7%  73.7%     ------------------------
 76 AY463060.1_SARS_coronavirus_ShanghaiQXC2_complete_genome        73.7%  73.7%     ------------------------
 77 AY485277.1_SARS_coronavirus_Sino1-11_complete_genome           100.0% 100.0%     ------------------------
 78 AY485278.1_SARS_coronavirus_Sino3-11_complete_genome           100.0% 100.0%     ------------------------
 79 AY502923.1_SARS_coronavirus_TW10_complete_genome               100.0% 100.0%     ------------------------
 80 AY502924.1_SARS_coronavirus_TW11_complete_genome               100.0% 100.0%     ------------------------
 81 AY502925.1_SARS_coronavirus_TW2_complete_genome                100.0% 100.0%     ------------------------
 82 AY502926.1_SARS_coronavirus_TW3_complete_genome                100.0% 100.0%     ------------------------
 83 AY502927.1_SARS_coronavirus_TW4_complete_genome                100.0% 100.0%     ------------------------
 84 AY502928.1_SARS_coronavirus_TW5_complete_genome                100.0% 100.0%     ------------------------
 85 AY502929.1_SARS_coronavirus_TW6_complete_genome                100.0% 100.0%     ------------------------
 86 AY502930.1_SARS_coronavirus_TW7_complete_genome                100.0% 100.0%     ------------------------
 87 AY502931.1_SARS_coronavirus_TW8_complete_genome                100.0% 100.0%     ------------------------
 88 AY502932.1_SARS_coronavirus_TW9_complete_genome                100.0% 100.0%     ------------------------
 89 AY508724.1_SARS_coronavirus_NS-1_complete_genome                94.7%  94.7%     ------------------------
 90 AY559081.1_SARS_coronavirus_Sin842_complete_genome              95.7%  95.3%     ------------------------
 91 AY559082.1_SARS_coronavirus_Sin852_complete_genome              99.7%  96.3%     ------------------------
 92 AY559083.1_SARS_coronavirus_Sin3408_complete_genome             88.7%  85.3%     ------------------------
 93 AY559084.1_SARS_coronavirus_Sin3765V_complete_genome            94.7%  94.7%     ------------------------
 94 AY559085.1_SARS_coronavirus_Sin848_complete_genome              94.7%  94.7%     ------------------------
 95 AY559086.1_SARS_coronavirus_Sin849_complete_genome              94.7%  94.7%     ------------------------
 96 AY559087.1_SARS_coronavirus_Sin3725V_complete_genome            94.7%  94.7%     ------------------------
 97 AY559088.1_SARS_coronavirus_SinP1_complete_genome               94.7%  94.7%     ------------------------
 98 AY559089.1_SARS_coronavirus_SinP2_complete_genome               94.7%  94.7%     ------------------------
 99 AY559090.1_SARS_coronavirus_SinP3_complete_genome               95.3%  92.7%     ------------------------
100 AY559091.1_SARS_coronavirus_SinP4_complete_genome               94.7%  94.7%     ------------------------
101 AY559092.1_SARS_coronavirus_SinP5_complete_genome               94.7%  94.7%     ------------------------
102 AY559093.1_SARS_coronavirus_Sin845_complete_genome              94.7%  94.7%     ------------------------
103 AY559094.1_SARS_coronavirus_Sin846_complete_genome              94.7%  94.7%     ------------------------
104 AY559095.1_SARS_coronavirus_Sin847_complete_genome              96.7%  96.0%     ------------------------
105 AY559096.1_SARS_coronavirus_Sin850_complete_genome              97.3%  95.7%     ------------------------
106 AY559097.1_SARS_coronavirus_Sin3408L_complete_genome            94.7%  94.7%     ------------------------
107 AY595412.1_SARS_coronavirus_LLJ-2004_complete_genome            93.0%  93.0%     ------------------------
```

```
108 AY654624.1_SARS_coronavirus_TJF_complete_genome                                  99.7%  99.0%     ------------------------
109 AY714217.1_SARS_Coronavirus_CDC*200301157_complete_genome                       100.0% 100.0%     ------------------------
110 CQ903009.1_Sequence_13_from_Patent_WO2004094667                                 100.0%  99.7%     ------------------------
111 CQ918584.1_Sequence_1_from_Patent_WO2004096842                                   95.0%  95.0%     ------------------------
112 CQ918585.1_Sequence_2_from_Patent_WO2004096842                                   95.0%  95.0%     ------------------------
113 CQ918598.1_Sequence_15_from_Patent_WO2004096842                                 100.0% 100.0%     ------------------------
114 NC_004718.3_SARS_coronavirus_complete_genome                                    100.0% 100.0%     ------------------------
    consensus/100%                                                                                    ........................
    consensus/90%                                                                                     ........................
    consensus/80%                                                                                     ........................
    consensus/70%                                                                                     ........................
```

MView 1.63, Copyright © 1997-2018 Nigel P. Brown
