# Supplementary material for: Potential use of noncoding RNAs and innovative therapeutic strategies to target the 5’UTR of SARS-CoV-2
Source: Epigenomics. 2020 Sep 2:10.2217/epi-2020-0162. doi: 10.2217/epi-2020-0162 (PMC7466951; doi:10.2217/epi-2020-0162)
Supplement: Supplementary file 2 [file epi-2020-0162-supp-table-2.pdf]

```
Reference sequence (1): hCoV-19/Algeria/G0638_2264/2020|EPI_ISL_418241|2020-03-02
Identities normalised by aligned length.
Colored by: identity

                                                                     cov    pid   1 [        .         .         .         .         :         .         .         . 80
 1 hCoV-19/Algeria/G0638_2264/2020|EPI_ISL_418241|2020-03-02       100.0% 100.0%    ---------------------------------------------------------CTTGTAGATCTGTTCTCTAAACG
 2 hCoV-19/Australia/NSW01/2020|EPI_ISL_407893|2020-01-24           97.9%  96.3%    --------------------------------------------------------------AGATCTGTTCTCTAAACG
 3 hCoV-19/bat/Yunnan/RaTG13/2013|EPI_ISL_402131|2013-07-24         86.4%  84.4%    -----------------------CTTTCCAGGTAACAAACCAACGAACTCTCGATCTCTTGTAGATCTGTTCTCTAAACG
 4 hCoV-19/Beijing/105/2020|EPI_ISL_413518|2020-01-26               82.6%  79.9%    ----ATTAAAGGTTTATAC----CTTCCCAGGTAACAAACCAACCAACTTTCGATCTCTTGTAGATCTGTTCTCTAAACG
 5 hCoV-19/Belgium/BA-02291/2020|EPI_ISL_415159|2020-02-29          90.9%  90.2%    ---------------------------------AACAAACCAACCAACTTTCGATCTCTTGTAGATCTGTTCTCTAAACG
 6 hCoV-19/Brazil/AMBR-02/2020|EPI_ISL_417034|2020-03-16           100.0%  98.0%    ------------------------------------------------------TCTCTTGTAGATCTGTTCTCTAAACG
 7 hCoV-19/Cambodia/0012/2020|EPI_ISL_411902|2020-01-27             82.2%  80.2%    -----TTAAAGGTTTATAC----CTTCCCAGGTAACAAACCAACCAACTTTCGATCTCTTGTAGATCTGTTCTCTAAACG
 8 hCoV-19/Canada/BC_02421/2020|EPI_ISL_415581|2020-03-01           97.9%  96.3%    --------------------------------------------------------------AGATCTGTTCTCTAAACG
 9 hCoV-19/Chile/Santiago_op2d1/2020|EPI_ISL_415658|2020-03-06      97.5%  97.9%    ---------------------------------------------------------------GATCTGTTCTCTAAACG
10 hCoV-19/China/IQTC01/2020|EPI_ISL_412966|2020-02-05              81.0%  78.6%    -GTTCTTTAAGGTTTATAC----CTTCCCAGGTAACAAACCAACCAACTTTCGATCTCTTGTAGATCTGTTCTCTAAACG
11 hCoV-19/China/WHU02/2020|EPI_ISL_406717|2020-01-02               83.1%  80.3%    -----TTAAAGGTTTATAC----CTTCCCAGGTAACAAACCAACCAACTTTCGATCTCTTGTAGATCTGTTCTCTAAACG
12 hCoV-19/Chongqing/IVDC-CQ-001/2020|EPI_ISL_408481|2020-01-18     81.4%  82.0%    -----------GTTTATAC----CTTCCCAGGTAACAAACCAACCAACTTTCGATCTCTTGTAGATCTGTTCTCTAAACG
13 hCoV-19/Congo/108/2020|EPI_ISL_417948|2020-03-19                 85.1%  82.1%    --------AAGGTTTATAC----CTTCCCAGGTAACAAACCAACCAACTTTCGATCTCTTGTAGATCTGTTCTCTAAACG
14 hCoV-19/Denmark/SSI-01/2020|EPI_ISL_416142|2020-02-26            91.7%  90.2%    ---------------------------------AACAAACCAACCAACTTTCGATCTCTTGTAGATCTGTTCTCTAAACG
15 hCoV-19/England/01/2020|EPI_ISL_407071|2020-01-29                85.1%  82.0%    ---------AGGTTTATAC----CTTCCCAGGTAACAAACCAACCAACTTTCGATCTCTTGTAGATCTGTTCTCTAAACG
16 hCoV-19/Finland/1/2020|EPI_ISL_407079|2020-01-29                 88.4%  84.9%    ----------------TAC----CTTCCCAGGTAACAAACCAACCAACTTTCGATCTCTTGTAGATCTGTTCTCTAAACG
17 hCoV-19/Foshan/20SF207/2020|EPI_ISL_406534|2020-01-22            90.9%  89.0%    -------------------------------GTAACAAACCAACCAACTTTCGATCTCTTGTAGATCTGTTCTCTAAACG
18 hCoV-19/France/ARA094100/2020|EPI_ISL_418412|2020-03-15          85.5%  84.8%    ----------------TAC----CTTCCCAGGTAACAAACCAACCAACTTTCGATCTCTTGTAGATCTGTTCTCTAAACG
19 hCoV-19/Fujian/13/2020|EPI_ISL_411066|2020-01-22                 83.5%  80.1%    ----ATTAAAGGTTTATAC----CTTCCCAGGTAACAAACCAACCAACTTTCGATCTCTTGTAGATCTGTTCTCTAAACG
20 hCoV-19/Georgia/Tb/2020|EPI_ISL_416482|2020-03-13                84.3%  81.6%    -------AAAGGTTTATAC----CTTCCCAGGTAACAAACCAACCAACTTTCGATCTCTTGTAGATCTGTTCTCTAAACG
21 hCoV-19/Germany/Baden-Wuerttemberg-1/2020|EPI_ISL_412912|2020-02-25 95.0% 99.1%  -------------------------------------------------------CTCTTGTAGATCTGTTCTCTAAACG
22 hCoV-19/Germany/BavPat1/2020|EPI_ISL_406862|2020-01-28           97.9%  96.7%    --------------------------------------------------------------AGATCTGTTCTCTAAACG
23 hCoV-19/Germany/NRW-01/2020|EPI_ISL_413488|2020-02-28            97.9%  95.9%    --------------------------------------------------------------AGATCTGTTCTCTAAACG
24 hCoV-19/Greece/10/2020|EPI_ISL_418263|2020-03-18                 83.5%  80.5%    ----ATTAAAGGTTTATAC----CTTCCCAGGTAACAAACCAACCAACTTTCGATCTCTTGTAGATCTGTTCTCTAAACG
25 hCoV-19/Guangdong/2020XN4243-P0035/2020|EPI_ISL_413853|2020-01-30 85.5% 88.0%    ------------------------------GGTAACAAACCAACCAACTTTCGATCTCTTGTAGATCTGTTCTCTAAACG
26 hCoV-19/Guangzhou/20SF206/2020|EPI_ISL_406533|2020-01-22         89.7%  88.9%    -------------------------------GTAACAAACCAACCAACTTTCGATCTCTTGTAGATCTGTTCTCTAAACG
27 hCoV-19/Hangzhou/HZ-1/2020|EPI_ISL_406970|2020-01-20             83.1%  81.0%    -------AAAGGTTTATAC----CTTCCCAGGTAACAAACCAACCAACTTTCGATCTCTTGTAGATCTGTTCTCTAAACG
28 hCoV-19/Hefei/2/2020|EPI_ISL_412026|2020-02-23                   84.3%  80.2%    ----ATTAAAGGTTTATAC----CTTCCCAGGTAACAAACCAACCAACTTTCGATCTCTTGTAGATCTGTTCTCTAAACG
29 hCoV-19/Hong_Kong/Case1_21012020/2020|EPI_ISL_417176|2020-01-21  97.5%  99.6%    --------------------------------------------------------------AGATCTGTTCTCTAAACG
30 hCoV-19/Hungary/2/2020|EPI_ISL_418183|2020-03-17                 97.9%  94.4%    --------------------------------------------------------------AGATCTGTTCTCTAAACG
31 hCoV-19/Iceland/1/2020|EPI_ISL_417535|2020-03-13                 87.2%  84.1%    -------------TTATAC----CTTCCCAGGTAACAAACCAACCAACTTTCGATCTCTTGTAGATCTGTTCTCTAAACG
32 hCoV-19/India/1-31/2020|EPI_ISL_413523|2020-01-31                91.3%  88.0%    ----------------------------CAGGTAACAAACCAACCAACTTTCGATCTCTTGTAGATCTGTTCTCTAAACG
33 hCoV-19/Ireland/21023/2020|EPI_ISL_418516|2020-03-06             81.8%  78.1%    ----nnnnnnnnnnnnnnn----nnnnnnnnnnnnnnnnnnnnnnnnnnnnnnnnnnnnnnnAGATCTGTTCTCTAAACG
34 hCoV-19/Israel/ISR_IT0320/2020|EPI_ISL_419210|2020-03            79.8%  78.5%    GGGGATTAAAGGTTTATAC----CTTCCCAGGTAACAAACCAACCAACTTTCGATCTCTTGTAGATCTGTTCTCTAAACG
35 hCoV-19/Italy/SPL1/2020|EPI_ISL_412974|2020-01-29                83.1%  80.0%    ----ATTAAAGGTTTATAC----CTTCCCAGGTAACAAACCAACCAACTTTCGATCTCTTGTAGATCTGTTCTCTAAACG
36 hCoV-19/Japan/DP0005/2020|EPI_ISL_416565|2020-02-15              88.4%  85.9%    --------------------------------------------------------------------------------
37 hCoV-19/Jiangsu/JS01/2020|EPI_ISL_411950|2020-01-23              82.2%  79.8%    ----ATTAAAGGTTTATAC----CTTCCCAGGTAACAAACCAACCAACTTTCGATCTCTTGTAGATCTGTTCTCTAAACG
38 hCoV-19/Jiangsu/JS02/2020|EPI_ISL_411952|2020-01-24              82.2%  79.8%    ----ATTAAAGGTTTATAC----CTTCCCAGGTAACAAACCAACCAACTTTCGATCTCTTGTAGATCTGTTCTCTAAACG
39 hCoV-19/Jiangxi/IVDC-JX-002/2020|EPI_ISL_408486|2020-01-11       85.5%  85.5%    -----------------------CTTCCCAGGTAACAAACCAACCAACTTTCGATCTCTTGTAGATCTGTTCTCTAAACG
40 hCoV-19/Jingzhou/HBCDC-HB-01/2020|EPI_ISL_412459|2020-01-08      83.5%  83.8%    ---------------ATAC----CTTCCCAGGTAACAAACCAACCAACTTTCGATCTCTTGTAGATCTGTTCTCTAAACG
41 hCoV-19/Kuwait/KU09/2020|EPI_ISL_416541|2020-03-02               83.1%  80.3%    -----TTAAAGGTTTATAC----CTTCCCAGGTAACAAACCAACCAACTTTCGATCTCTTGTAGATCTGTTCTCTAAACG
42 hCoV-19/Lithuania/ChVir1632/2020|EPI_ISL_416741|2020-02          85.1%  84.4%    ---------------ATAC----CTTCCCAGGTAACAAACCAACCAACTTTCGATCTCTTGTAGATCTGTTCTCTAAACG
43 hCoV-19/Luxembourg/LNS0158952/2020|EPI_ISL_417534|2020-03-18     78.5%  79.5%    ----ATTAAAGGTTTATAC----CTTCCCAGGTAACAAACCAACCAACTTTCGATCTCTTGTAGATCTGTTCTCTAAACG
44 hCoV-19/Malaysia/188407/2020|EPI_ISL_417918|2020-03-18           95.5%  93.9%    -------------------------------------------CCAACTTTCGATCTCTTGTAGATCTGTTCTCTAAACG
45 hCoV-19/Mexico/CDMX-InDRE_01/2020|EPI_ISL_412972|2020-02-27      78.9%  79.6%    ----ATTAAAGGTTTATAC----CTTCCCAGGTAACAAACCAACCAACTTTCGATCTCTTGTAGATCTGTTCTCTAAACG
46 hCoV-19/NanChang/JX216/2020|EPI_ISL_417420|2020-03-23            91.7%  90.2%    ---------------------------------AACAAACCAACCAACTTTCGATCTCTTGTAGATCTGTTCTCTAAACG
47 hCoV-19/Nepal/61/2020|EPI_ISL_410301|2020-01-13                  90.1%  86.1%    -----------------------CTTCCCAGGTAACAAACCAACCAACTTTCGATCTCTTGTAGATCTGTTCTCTAAACG
48 hCoV-19/Netherlands/Utrecht_1/2020|EPI_ISL_414435|2020-03-03     96.3%  97.5%    ---------------------------------------------------CGATCTCTTGTAGATCTGTTCTCTAAACG
49 hCoV-19/New_Zealand/01/2020|EPI_ISL_413490|2020-02-27            97.9%  95.9%    --------------------------------------------------------------AGATCTGTTCTCTAAACG
50 hCoV-19/Nonthaburi/61/2020|EPI_ISL_403962|2020-01-08             86.4%  84.2%    ---------------ATAC----CTTCCCAGGTAACAAACCAACCAACTTTCGATCTCTTGTAGATCTGTTCTCTAAACG
51 hCoV-19/Norway/1379/2020|EPI_ISL_417483|2020-02-27               85.5%  82.7%    -----------GTTTATAC----CTTCCCAGGTAACAAACCAACCAACTTTCGATCTCTTGTAGATCTGTTCTCTAAACG
52 hCoV-19/Pakistan/Gilgit1/2020|EPI_ISL_417444|2020-03-04          95.5%  94.7%    --------------------------------------------CAACTTTCGATCTCTTGTAGATCTGTTCTCTAAACG
53 hCoV-19/Panama/328677/2020|EPI_ISL_415152|2020-03-06             81.8%  79.8%    ----ATTAAAGGTTTATAC----CTTCCCAGGTAACAAACCAACCAACTTTCGATCTCTTGTAGATCTGTTCTCTAAACG
54 hCoV-19/pangolin/Guangdong/1/2019|EPI_ISL_410721|2019           81.4%  78.9%    ----ATTAAAGGTTTATAC----CTTCCCAGGTAACAAACCAACCAACTCTCGATCTCTTGTAGATCTGTTCTCTAAACG
55 hCoV-19/Peru/010/2020|EPI_ISL_415787|2020-03-10                  87.6%  84.1%    -------------TTATAC----CTTCCCAGGTAACAAACCAACCAACTTTCGATCTCTTGTAGATCTGTTCTCTAAACG
56 hCoV-19/Poland/PL_P1/2020|EPI_ISL_416488|2020-03-03              82.2%  79.4%    ----ATTAAAGGTTTATAC----CTTCCCAGGTAACAAACCAACCAACTTTCGATCTCTTGTAGATCTGTTCTCTAAACG
57 hCoV-19/Portugal/CV62/2020|EPI_ISL_413647|2020-03-01             81.8%  80.2%    ----ATTAAAGGTTTATAC----CTTCCCAGGTAACAAACCAACCAACTTTCGATCTCTTGTAGATCTGTTCTCTAAACG
58 hCoV-19/Russia/StPetersburg-3524/2020|EPI_ISL_415710|2020-03-15  77.3%  79.2%    ----ATTAAAGGTTTATAC----CTTCCCAGGTAACAAACCAACCAACTTTCGATCTCTTGTAGATCTGTTCTCTAAACG
59 hCoV-19/Saudi_Arabia/KAIMRC-Alghoribi/2020|EPI_ISL_416432|2020-03-07 72.7% 76.2% --CCTGGGAAGGTATAAACCTTACTTCCCAGGTAACAAACCAACCAACTTTCGATCTCTTGTAGATCTGTTCTCTAAACG
60 hCoV-19/Scotland/CVR05/2020|EPI_ISL_414027|2020-03-04            97.9%  96.3%    --------------------------------------------------------------AGATCTGTTCTCTAAACG
61 hCoV-19/Senegal/003/2020|EPI_ISL_418206|2020-02-28               96.7%  95.5%    ----------------------------------------------ACTTTCGATCTCTTGTAGATCTGTTCTCTAAACG
62 hCoV-19/Shandong/IVDC-SD-001/2020|EPI_ISL_408482|2020-01-19      83.9%  84.2%    ----------------TAC----CTTCCCAGGTAACAAACCAACCAACTTTCGATCTCTTGTAGATCTGTTCTCTAAACG
63 hCoV-19/Shanghai/SH0002/2020|EPI_ISL_416316|2020-01-25           93.4%  91.8%    --------------------------------------ACCAACCAACTTTCGATCTCTTGTAGATCTGTTCTCTAAACG
64 hCoV-19/Shenzhen/HKU-SZ-002/2020|EPI_ISL_406030|2020-01-10       92.6%  92.5%    ----------------------------------------CAACCAACTTTCGATCTCTTGTAGATCTGTTCTCTAAACG
65 hCoV-19/Sichuan/IVDC-SC-001/2020|EPI_ISL_408484|2020-01-15       85.5%  85.5%    -----------------------CTTCCCAGGTAACAAACCAACCAACTTTCGATCTCTTGTAGATCTGTTCTCTAAACG
66 hCoV-19/Singapore/1/2020|EPI_ISL_406973|2020-01-23               82.6%  79.9%    ----ATTAAAGGTTTATAC----CTTCCCAGGTAACAAACCAACCAACTTTCGATCTCTTGTAGATCTGTTCTCTAAACG
67 hCoV-19/Slovakia/SK-BMC1/2020|EPI_ISL_417877|2020-03-06          81.4%  80.7%    ------TAAAGGTTTATAC----CTTCCCAGGTAACAAACCAACCAACTTTCGATCTCTTGTAGATCTGTTCTCTAAACG
68 hCoV-19/South_Africa/R03006-20/2020|EPI_ISL_417186|2020-03-07    78.1%  79.4%    ----ATTAAAGGTTTATAC----CTTCCCAGGTAACAAACCAACCAACTTTCGATCTCTTGTAGATCTGTTCTCTAAACG
69 hCoV-19/South_Korea/KCDC03/2020|EPI_ISL_407193|2020-01-25        91.3%  90.9%    -------------------------------------AAACCAACCAACTTTCGATCTCTTGTAGATCTGTTCTCTAAACG
70 hCoV-19/Spain/Madrid_H10_39/2020|EPI_ISL_417963|2020-03-12       95.9%  96.3%    ------------------------------------------------TTTCGATCTCTTGTAGATCTGTTCTCTAAACG
71 hCoV-19/Sweden/01/2020|EPI_ISL_411951|2020-02-07                 83.5%  80.1%    ----ATTAAAGGTTTATAC----CTTCCCAGGTAACAAACCAACCAACTTTCGATCTCTTGTAGATCTGTTCTCTAAACG
72 hCoV-19/Switzerland/1000477377/2020|EPI_ISL_413020|2020-02-27    78.1%  79.4%    ----ATTAAAGGTTTATAC----CTTCCCAGGTAACAAACCAACCAACTTTCGATCTCTTGTAGATCTGTTCTCTAAACG
73 hCoV-19/Taiwan/2/2020|EPI_ISL_406031|2020-01-23                  85.1%  81.3%    -------AAAGGTTTATAC----CTTCCCAGGTAACAAACCAACCAACTTTCGATCTCTTGTAGATCTGTTCTCTAAACG
74 hCoV-19/USA/AZ1/2020|EPI_ISL_406223|2020-01-22                   84.3%  80.2%    ----ATTAAAGGTTTATAC----CTTCCCAGGTAACAAACCAACCAACTTTCGATCTCTTGTAGATCTGTTCTCTAAACG
75 hCoV-19/Vietnam/19-01S/2020|EPI_ISL_418269|2020-01-22            81.4%  79.7%    ----ATTAAAGGTTTATAC----CTTCCCAGGTAACAAACCAACCAACTTTCGATCTCTTGTAGATCTGTTCTCTAAACG
76 hCoV-19/Wales/PHW04/2020|EPI_ISL_415041|2020-03-07               97.9%  95.2%    --------------------------------------------------------------AGATCTGTTCTCTAAACG
77 hCoV-19/Wuhan/HBCDC-HB-01/2019|EPI_ISL_402132|2019-12-30         84.7%  84.0%    ---------------ATAC----CTTCCCAGGTAACAAACCAACCAACTTTCGATCTCTTGTAGATCTGTTCTCTAAACG
78 hCoV-19/Wuhan-Hu-1/2019|EPI_ISL_402125|2019-12-31                83.1%  80.0%    ----ATTAAAGGTTTATAC----CTTCCCAGGTAACAAACCAACCAACTTTCGATCTCTTGTAGATCTGTTCTCTAAACG
79 hCoV-19/Yunnan/IVDC-YN-003/2020|EPI_ISL_408480|2020-01-17        82.2%  81.8%    ----------GGTTTATAC----CTTCCCAGGTAACAAACCAACCAACTTTCGATCTCTTGTAGATCTGTTCTCTAAACG
80 hCoV-19/Zhejiang/WZ-01/2020|EPI_ISL_404227|2020-01-16            89.3%  87.4%    ---------------------------CCAGGTAACAAGCCAACCAACTTTCGATCTCTTGTAGATCTGTTCTCTAAACG
   consensus/100%                                                                   ................................................................................
   consensus/90%                                                                    ..............................................................AGATCTGTTCTCTAAACG
   consensus/80%                                                                    ..............................................ssTsTCGATCTCTTGTAGATCTGTTCTCTAAACG
   consensus/70%                                                                    .................................AACAAACCAACCAACTTTCGATCTCTTGTAGATCTGTTCTCTAAACG

                                                                     cov    pid  81        .         1         .         .         .         .         :         . 160
 1 hCoV-19/Algeria/G0638_2264/2020|EPI_ISL_418241|2020-03-02       100.0% 100.0%    AACTTTAAAATCTGTGTGGCTGTCACTCGGCTGCATGCTTAGTGCACTCACGCAGTATAATTAATAACTAATTACTGTCG
 2 hCoV-19/Australia/NSW01/2020|EPI_ISL_407893|2020-01-24           97.9%  96.3%    AACTTTAAAATCTGTGTGGCTGTCACTCGGCTGCATGCTTAGTGCACTCACGCAGTATAATTAATAACTAATTACTGTCG
 3 hCoV-19/bat/Yunnan/RaTG13/2013|EPI_ISL_402131|2013-07-24         86.4%  84.4%    AACTTTAAAATCTGTGTGACTGTCACTCGGCTGCATGCTTAGTGCACTCACGCAGTATAATTAATAACTAATTACTGTCG
 4 hCoV-19/Beijing/105/2020|EPI_ISL_413518|2020-01-26               82.6%  79.9%    AACTTTAAAATCTGTGTGGCTGTCACTCGGCTGCATGCTTAGTGCACTCACGCAGTATAATTAATAACTAATTACTGTCG
 5 hCoV-19/Belgium/BA-02291/2020|EPI_ISL_415159|2020-02-29          90.9%  90.2%    AACTTTAAAATCTGTGTGGCTGTCACTCGGCTGCATGCTTAGTGCACTCACGCAGTATAATTAATAACTAATTACTGTCG
 6 hCoV-19/Brazil/AMBR-02/2020|EPI_ISL_417034|2020-03-16           100.0%  98.0%    AACTTTAAAATCTGTGTGGCTGTCACTCGGCTGCATGCTTAGTGCACTCACGCAGTATAATTAATAACTAATTACTGTCG
 7 hCoV-19/Cambodia/0012/2020|EPI_ISL_411902|2020-01-27             82.2%  80.2%    AACTTTAAAATCTGTGTGGCTGTCACTCGGCTGCATGCTTAGTGCACTCACGCAGTATAATTAATAACTAATTACTGTCG
 8 hCoV-19/Canada/BC_02421/2020|EPI_ISL_415581|2020-03-01           97.9%  96.3%    AACTTTAAAATCTGTGTGGCTGTCACTCGGCTGCATGCTTAGTGCACTCACGCAGTATAATTAATAACTAATTACTGTCG
 9 hCoV-19/Chile/Santiago_op2d1/2020|EPI_ISL_415658|2020-03-06      97.5%  97.9%    AACTTTAAAATCTGTGTGGCTGTCACTCGGCTGCATGCTTAGTGCACTCACGCAGTATAATTAATAACTAATTACTGTCG
10 hCoV-19/China/IQTC01/2020|EPI_ISL_412966|2020-02-05              81.0%  78.6%    AACTTTAAAATCTGTGTGGCTGTCACTCGGCTGCATGCTTAGTGCACTCACGCAGTATAATTAATAACTAATTACTGTCG
11 hCoV-19/China/WHU02/2020|EPI_ISL_406717|2020-01-02               83.1%  80.3%    AACTTTAAAATCTGTGTGGCTGTCACTCGGCTGCATGCTTAGTGCACTCACGCAGTATAATTAATAACTAATTACTGTCG
12 hCoV-19/Chongqing/IVDC-CQ-001/2020|EPI_ISL_408481|2020-01-18     81.4%  82.0%    AACTTTAAAATCTGTGTGGCTGTCACTCGGCTGCATGCTTAGTGCACTCACGCAGTATAATTAATAACTAATTACTGTCG
13 hCoV-19/Congo/108/2020|EPI_ISL_417948|2020-03-19                 85.1%  82.1%    AACTTTAAAATCTGTGTGGCTGTCACTCGGCTGCATGCTTAGTGCACTCACGCAGTATAATTAATAACTAATTACTGTCG
14 hCoV-19/Denmark/SSI-01/2020|EPI_ISL_416142|2020-02-26            91.7%  90.2%    AACTTTAAAATCTGTGTGGCTGTCACTCGGCTGCATGCTTAGTGCACTCACGCAGTATAATTAATAACTAATTACTGTCG
15 hCoV-19/England/01/2020|EPI_ISL_407071|2020-01-29                85.1%  82.0%    AACTTTAAAATCTGTGTGGCTGTCACTCGGCTGCATGCTTAGTGCACTCACGCAGTATAATTAATAACTAATTACTGTCG
16 hCoV-19/Finland/1/2020|EPI_ISL_407079|2020-01-29                 88.4%  84.9%    AACTTTAAAATCTGTGTGGCTGTCACTCGGCTGCATGCTTAGTGCACTCACGCAGTATAATTAATAACTAATTACTGTCG
17 hCoV-19/Foshan/20SF207/2020|EPI_ISL_406534|2020-01-22            90.9%  89.0%    AACTTTAAAATCTGTGTGGCTGTCACTCGGCTGCATGCTTAGTGCACTCACGCAGTATAATTAATAACTAATTACTGTCG
18 hCoV-19/France/ARA094100/2020|EPI_ISL_418412|2020-03-15          85.5%  84.8%    AACTTTAAAATCTGTGTGGCTGTCACTCGGCTGCATGCTTAGTGCACTCACGCAGTATAATTAATAACTAATTACTGTCG
19 hCoV-19/Fujian/13/2020|EPI_ISL_411066|2020-01-22                 83.5%  80.1%    AACTTTAAAATCTGTGTGGCTGTCACTCGGCTGCATGCTTAGTGCACTCACGCAGTATAATTAATAACTAATTACTGTCG
20 hCoV-19/Georgia/Tb/2020|EPI_ISL_416482|2020-03-13                84.3%  81.6%    AACTTTAAAATCTGTGTGGCTGTCACTCGGCTGCATGCTTAGTGCACTCACGCAGTATAATTAATAACTAATTACTGTCG
21 hCoV-19/Germany/Baden-Wuerttemberg-1/2020|EPI_ISL_412912|2020-02-25 95.0% 99.1%  AACTTTAAAATCTGTGTGGCTGTCACTCGGCTGCATGCTTAGTGCACTCACGCAGTATAATTAATAACTAATTACTGTCG
22 hCoV-19/Germany/BavPat1/2020|EPI_ISL_406862|2020-01-28           97.9%  96.7%    AACTTTAAAATCTGTGTGGCTGTCACTCGGCTGCATGCTTAGTGCACTCACGCAGTATAATTAATAACTAATTACTGTCG
23 hCoV-19/Germany/NRW-01/2020|EPI_ISL_413488|2020-02-28            97.9%  95.9%    AACTTTAAAATCTGTGTGGCTGTCACTCGGCTGCATGCTTAGTGCACTCACGCAGTATAATTAATAACTAATTACTGTCG
24 hCoV-19/Greece/10/2020|EPI_ISL_418263|2020-03-18                 83.5%  80.5%    AACTTTAAAATCTGTGTGGCTGTCACTCGGCTGCATGCTTAGTGCACTCACGCAGTATAATTAATAACTAATTACTGTCG
25 hCoV-19/Guangdong/2020XN4243-P0035/2020|EPI_ISL_413853|2020-01-30 85.5% 88.0%    AACTTTAAAATCTGTGTGGCTGTCACTCGGCTGCATGCTTAGTGCACTCACGCAGTATAATTAATAACTAATTACTGTCG
26 hCoV-19/Guangzhou/20SF206/2020|EPI_ISL_406533|2020-01-22         89.7%  88.9%    AACTTTAAAATCTGTGTGGCTGTCACTCGGCTGCATGCTTAGTGCACTCACGCAGTATAATTAATAACTAATTACTGTCG
27 hCoV-19/Hangzhou/HZ-1/2020|EPI_ISL_406970|2020-01-20             83.1%  81.0%    AACTTTAAAATCTGTGTGGCTGTCACTCGGCTGCATGCTTAGTGCACTCACGCAGTATAATTAATAACTAATTACTGTCG
28 hCoV-19/Hefei/2/2020|EPI_ISL_412026|2020-02-23                   84.3%  80.2%    AACTTTAAAATCTGTGTGGCTGTCACTCGGCTGCATGCTTAGTGCACTCACGCAGTATAATTAATAACTAATTACTGTCG
29 hCoV-19/Hong_Kong/Case1_21012020/2020|EPI_ISL_417176|2020-01-21  97.5%  99.6%    AACTTTAAAATCTGTGTGGCTGTCACTCGGCTGCATGCTTAGTGCACTCACGCAGTATAATTAATAACTAATTACTGTCG
30 hCoV-19/Hungary/2/2020|EPI_ISL_418183|2020-03-17                 97.9%  94.4%    AACTTTAAAATCTGTGTGGCTGTCACTCGGCTGCATGCTTAGTGCACTCACGCAGTATAATTAATAACTAATTACTGTCG
31 hCoV-19/Iceland/1/2020|EPI_ISL_417535|2020-03-13                 87.2%  84.1%    AACTTTAAAATCTGTGTGGCTGTCACTCGGCTGCATGCTTAGTGCACTCACGCAGTATAATTAATAACTAATTACTGTCG
32 hCoV-19/India/1-31/2020|EPI_ISL_413523|2020-01-31                91.3%  88.0%    AACTTTAAAATCTGTGTGGCTGTCACTCGGCTGCATGCTTAGTGCACTCACGCAGTATAATTAATAACTAATTACTGTCG
33 hCoV-19/Ireland/21023/2020|EPI_ISL_418516|2020-03-06             81.8%  78.1%    AACTTTAAAATCTGTGTGGCTGTCACTCGGCTGCATGCTTAGTGCACTCACGCAGTATAATTAATAACTAATTACTGTCG
34 hCoV-19/Israel/ISR_IT0320/2020|EPI_ISL_419210|2020-03            79.8%  78.5%    AACTTTAAAATCTGTGTGGCTGTCACTCGGCTGCATGCTTAGTGCACTCACGCAGTATAATTAATAACTAATTACTGTCG
35 hCoV-19/Italy/SPL1/2020|EPI_ISL_412974|2020-01-29                83.1%  80.0%    AACTTTAAAATCTGTGTGGCTGTCACTCGGCTGCATGCTTAGTGCACTCACGCAGTATAATTAATAACTAATTACTGTCG
36 hCoV-19/Japan/DP0005/2020|EPI_ISL_416565|2020-02-15              88.4%  85.9%    -----TAAAATCTGTGTGGCTGTCACTCGGCTGCATGCTTAGTGCACTCACGCAGTATAATTAATAACTAATTACTGTCG
37 hCoV-19/Jiangsu/JS01/2020|EPI_ISL_411950|2020-01-23              82.2%  79.8%    AACTTTAAAATCTGTGTGGCTGTCACTCGGCTGCATGCTTAGTGCACTCACGCAGTATAATTAATAACTAATTACTGTCG
38 hCoV-19/Jiangsu/JS02/2020|EPI_ISL_411952|2020-01-24              82.2%  79.8%    AACTTTAAAATCTGTGTGGCTGTCACTCGGCTGCATGCTTAGTGCACTCACGCAGTATAATTAATAACTAATTACTGTCG
39 hCoV-19/Jiangxi/IVDC-JX-002/2020|EPI_ISL_408486|2020-01-11       85.5%  85.5%    AACTTTAAAATCTGTGTGGCTGTCACTCGGCTGCATGCTTAGTGCACTCACGCAGTATAATTAATAACTAATTACTGTCG
40 hCoV-19/Jingzhou/HBCDC-HB-01/2020|EPI_ISL_412459|2020-01-08      83.5%  83.8%    AACTTTAAAATCTGTGTGGCTGTCACTCGGCTGCATGCTTAGTGCACTCACGCAGTATAATTAATAACTAATTACTGTCG
41 hCoV-19/Kuwait/KU09/2020|EPI_ISL_416541|2020-03-02               83.1%  80.3%    AACTTTAAAATCTGTGTGGCTGTCACTCGGCTGCATGCTTAGTGCACTCACGCAGTATAATTAATAACTAATTACTGTCG
42 hCoV-19/Lithuania/ChVir1632/2020|EPI_ISL_416741|2020-02          85.1%  84.4%    AACTTTAAAATCTGTGTGGCTGTCACTCGGCTGCATGCTTAGTGCACTCACGCAGTATAATTAATAACTAATTACTGTCG
43 hCoV-19/Luxembourg/LNS0158952/2020|EPI_ISL_417534|2020-03-18     78.5%  79.5%    AACTTTAAAATCTGTGTGGCTGTCACTCGGCTGCATGCTTAGTGCACTCACGCAGTATAATTAATAACTAATTACTGTCG
44 hCoV-19/Malaysia/188407/2020|EPI_ISL_417918|2020-03-18           95.5%  93.9%    AACTTTAAAATCTGTGTGGCTGTCACTCGGCTGCATGCTTAGTGCACTCACGCAGTATAATTAATAACTAATTACTGTCG
45 hCoV-19/Mexico/CDMX-InDRE_01/2020|EPI_ISL_412972|2020-02-27      78.9%  79.6%    AACTTTAAAATCTGTGTGGCTGTCACTCGGCTGCATGCTTAGTGCACTCACGCAGTATAATTAATAACTAATTACTGTCG
```

```
46 hCoV-19/NanChang/JX216/2020|EPI_ISL_417420|2020-03-23                91.7%  90.2%     AACTTTAAAATCTGTGTGGCTGTCACTCGGCTGCATGCTTAGTGCACTCACGCAGTATAATTAATAACTAATTACTGTCG
47 hCoV-19/Nepal/61/2020|EPI_ISL_410301|2020-01-13                      90.1%  86.1%     AACTTTAAAATCTGTGTGGCTGTCACTCGGCTGCATGCTTAGTGCACTCACGCAGTATAATTAATAACTAATTACTGTCG
48 hCoV-19/Netherlands/Utrecht_1/2020|EPI_ISL_414435|2020-03-03         96.3%  97.5%     AACTTTAAAATCTGTGTGGCTGTCACTCGGCTGCATGCTTAGTGCACTCACGCAGTATAATTAATAACTAATTACTGTCG
49 hCoV-19/New_Zealand/01/2020|EPI_ISL_413490|2020-02-27               97.9%  95.9%     AACTTTAAAATCTGTGTGGCTGTCACTCGGCTGCATGCTTAGTGCACTCACGCAGTATAATTAATAACTAATTACTGTCG
50 hCoV-19/Nonthaburi/61/2020|EPI_ISL_403962|2020-01-08                 86.4%  84.2%     AACTTTAAAATCTGTGTGGCTGTCACTCGGCTGCATGCTTAGTGCACTCACGCAGTATAATTAATAACTAATTACTGTCG
51 hCoV-19/Norway/1379/2020|EPI_ISL_417483|2020-02-27                   85.5%  82.7%     AACTTTAAAATCTGTGTGGCTGTCACTCGGCTGCATGCTTAGTGCACTCACGCAGTATAATTAATAACTAATTACTGTCG
52 hCoV-19/Pakistan/Gilgit1/2020|EPI_ISL_417444|2020-03-04              95.5%  94.7%     AACTTTAAAATCTGTGTGGCTGTCACTCGGCTGCATGCTTAGTGCACTCACGCAGTATAATTAATAACTAATTACTGTCG
53 hCoV-19/Panama/328677/2020|EPI_ISL_415152|2020-03-06                 81.8%  79.8%     AACTTKAAAATCTGTGTGGCTGTCACTCGGCTGCATGCTTAGTGCACTCACGCAGTATAATTAATAACTAATTACTGTCG
54 hCoV-19/pangolin/Guangdong/1/2019|EPI_ISL_410721|2019                81.4%  78.9%     AACTTTAAAATCTGTGTGGCTGTCACTCGGCTGCATGCTTAGTGCACTCACGCAGTATAATTAATAACTAATTACTGTCG
55 hCoV-19/Peru/010/2020|EPI_ISL_415787|2020-03-10                      87.6%  84.1%     AACTTTAAAATCTGTGTGGCTGTCACTCGGCTGCATGCTTAGTGCACTCACGCAGTATAATTAATAACTAATTACTGTCG
56 hCoV-19/Poland/PL_P1/2020|EPI_ISL_416488|2020-03-03                  82.2%  79.4%     AACTTTAAAATCTGTGTGGCTGTCACTCGGCTGCATGCTTAGTGCACTCACGCAGTATAATTAATAACTAATTACTGTCG
57 hCoV-19/Portugal/CV62/2020|EPI_ISL_413647|2020-03-01                 81.8%  80.2%     AACTTTAAAATCTGTGTGGCTGTCACTCGGCTGCATGCTTAGTGCACTCACGCAGTATAATTAATAACTAATTACTGTCG
58 hCoV-19/Russia/StPetersburg-3524/2020|EPI_ISL_415710|2020-03-15      77.3%  79.2%     AACTTTAAAATCTGTGTGGCTGTCACTCGGCTGCATGCTTAGTGCACTCACGCAGTATAATTAATAACTAATTACTGTCG
59 hCoV-19/Saudi_Arabia/KAIMRC-Alghoribi/2020|EPI_ISL_416432|2020-03-07 72.7%  76.2%     AACTTTAAAATCTGTGTGGCTGTCACTCGGCTGCATGCTTAGTGCACTCACGCAGTATAATTAATAACTAATTACTGTCG
60 hCoV-19/Scotland/CVR05/2020|EPI_ISL_414027|2020-03-04                97.9%  96.3%     AACTTTAAAATCTGTGTGGCTGTCACTCGGCTGCATGCTTAGTGCACTCACGCAGTATAATTAATAACTAATTACTGTCG
61 hCoV-19/Senegal/003/2020|EPI_ISL_418206|2020-02-28                   96.7%  95.5%     AACTTTAAAATCTGTGTGGCTGTCACTCGGCTGCATGCTTAGTGCACTCACGCAGTATAATTAATAACTAATTACTGTCG
62 hCoV-19/Shandong/IVDC-SD-001/2020|EPI_ISL_408482|2020-01-19          83.9%  84.2%     AACTTTAAAATCTGTGTGGCTGTCACTCGGCTGCATGCTTAGTGCACTCACGCAGTATAATTAATAACTAATTACTGTCG
63 hCoV-19/Shanghai/SH0002/2020|EPI_ISL_416316|2020-01-25               93.4%  91.8%     AACTTTAAAATCTGTGTGGCTGTCACTCGGCTGCATGCTTAGTGCACTCACGCAGTATAATTAATAACTAATTACTGTCG
64 hCoV-19/Shenzhen/HKU-SZ-002/2020|EPI_ISL_406030|2020-01-10           92.6%  92.5%     AACTTTAAAATCTGTGTGGCTGTCACTCGGCTGCATGCTTAGTGCACTCACGCAGTATAATTAATAACTAATTACTGTCG
65 hCoV-19/Sichuan/IVDC-SC-001/2020|EPI_ISL_408484|2020-01-15           85.5%  85.5%     AACTTTAAAATCTGTGTGGCTGTCACTCGGCTGCATGCTTAGTGCACTCACGCAGTATAATTAATAACTAATTACTGTCG
66 hCoV-19/Singapore/1/2020|EPI_ISL_406973|2020-01-23                   82.6%  79.9%     AACTTTAAAATCTGTGTGGCTGTCACTCGGCTGCATGCTTAGTGCACTCACGCAGTATAATTAATAACTAATTACTGTCG
67 hCoV-19/Slovakia/SK-BMC1/2020|EPI_ISL_417877|2020-03-06              81.4%  80.7%     AACTTTAAAATCTGTGTGGCTGTCACTCGGCTGCATGCTTAGTGCACTCACGCAGTATAATTAATAACTAATTACTGTCG
68 hCoV-19/South_Africa/R03006-20/2020|EPI_ISL_417186|2020-03-07        78.1%  79.4%     AACTTTAAAATCTGTGTGGCTGTCACTCGGCTGCATGCTTAGTGCACTCACGCAGTATAATTAATAACTAATTACTGTCG
69 hCoV-19/South_Korea/KCDC03/2020|EPI_ISL_407193|2020-01-25            91.3%  90.9%     AACTTTAAAATCTGTGTGGCTGTCACTCGGCTGCATGCTTAGTGCACTCACGCAGTATAATTAATAACTAATTACTGTCG
70 hCoV-19/Spain/Madrid_H10_39/2020|EPI_ISL_417963|2020-03-12           95.9%  96.3%     AACTTTAAAATCTGTGTGGCTGTCACTCGGCTGCATGCTTAGTGCACTCACGCAGTATAATTAATAACTAATTACTGTCG
71 hCoV-19/Sweden/01/2020|EPI_ISL_411951|2020-02-07                     83.5%  80.1%     AACTTTAAAATCTGTGTGGCTGTCACTCGGCTGCATGCTTAGTGCACTCACGCAGTATAATTAATAACTAATTACTGTCG
72 hCoV-19/Switzerland/1000477377/2020|EPI_ISL_413020|2020-02-27        78.1%  79.4%     AACTTTAAAATCTGTGTGGCTGTCACTCGGCTGCATGCTTAGTGCACTCACGCAGTATAATTAATAACTAATTACTGTCG
73 hCoV-19/Taiwan/2/2020|EPI_ISL_406031|2020-01-23                      85.1%  81.3%     AACTTTAAAATCTGTGTGGCTGTCACTCGGCTGCATGCTTAGTGCACTCACGCAGTATAATTAATAACTAATTACTGTCG
74 hCoV-19/USA/AZ1/2020|EPI_ISL_406223|2020-01-22                       84.3%  80.2%     AACTTTAAAATCTGTGTGGCTGTCACTCGGCTGCATGCTTAGTGCACTCACGCAGTATAATTAATAACTAATTACTGTCG
75 hCoV-19/Vietnam/19-01S/2020|EPI_ISL_418269|2020-01-22                81.4%  79.7%     AACTTTAAAATCTGTGTGGCTGTCACTCGGCTGCATGCTTAGTGCACTCACGCAGTATAATTAATAACTAATTACTGTCG
76 hCoV-19/Wales/PHW04/2020|EPI_ISL_415041|2020-03-07                   97.9%  95.2%     AACTTTAAAATCTGTGTGGCTGTCACTCGGCTGCATGCTTAGTGCACTCACGCAGTATAATTAATAACTAATTACTGTCG
77 hCoV-19/Wuhan/HBCDC-HB-01/2019|EPI_ISL_402132|2019-12-30             84.7%  84.0%     AACTTTAAAATCTGTGTGGCTGTCACTCGGCTGCATGCTTAGTGCACTCACGCAGTATAATTAATAACTAATTACTGTCG
78 hCoV-19/Wuhan-Hu-1/2019|EPI_ISL_402125|2019-12-31                    83.1%  80.0%     AACTTTAAAATCTGTGTGGCTGTCACTCGGCTGCATGCTTAGTGCACTCACGCAGTATAATTAATAACTAATTACTGTCG
79 hCoV-19/Yunnan/IVDC-YN-003/2020|EPI_ISL_408480|2020-01-17            82.2%  81.8%     AACTTTAAAATCTGTGTGGCTGTCACTCGGCTGCATGCTTAGTGCACTCACGCAGTATAATTAATAACTAATTACTGTCG
80 hCoV-19/Zhejiang/WZ-01/2020|EPI_ISL_404227|2020-01-16                89.3%  87.4%     AACTTTAAAATCTGTGTGGCTGTCACTCGGCTGCATGCTTAGTGCACTCACGCAGTATAATTAATAACTAATTACTGTCG
   consensus/100%                                                                        .....pAAAATCTGTGTGuCTGTCACTCGGCTGCATGCTTAGTGCACTCACGCAGTATAATTAATAACTAATTACTGTCG
   consensus/90%                                                                         AACTTTAAAATCTGTGTGGCTGTCACTCGGCTGCATGCTTAGTGCACTCACGCAGTATAATTAATAACTAATTACTGTCG
   consensus/80%                                                                         AACTTTAAAATCTGTGTGGCTGTCACTCGGCTGCATGCTTAGTGCACTCACGCAGTATAATTAATAACTAATTACTGTCG
   consensus/70%                                                                         AACTTTAAAATCTGTGTGGCTGTCACTCGGCTGCATGCTTAGTGCACTCACGCAGTATAATTAATAACTAATTACTGTCG

                                                                          cov    pid 161          .         .         .         2         .         .         .         . 240
 1 hCoV-19/Algeria/G0638_2264/2020|EPI_ISL_418241|2020-03-02           100.0% 100.0%     TTGACAGGACACGAGTAACTCGTCTATCTTCTGCAGGCTGCTTACGGTTTCGTCCGTGTTGCAGCCGATCATCAGCACAT
 2 hCoV-19/Australia/NSW01/2020|EPI_ISL_407893|2020-01-24               97.9%  96.3%     TTGACAGGACACGAGTAACTCGTCTATCTTCTGCAGGCTGCTTACGGTTTCGTCCGTGTTGCAGCCGATCATCAGCACAT
 3 hCoV-19/bat/Yunnan/RaTG13/2013|EPI_ISL_402131|2013-07-24             86.4%  84.4%     TTGACAGGACACGAGTAACTCATCTATCTTCTGCAGGTTGCTTACGGTTTCGTCCGTGTTGCAGCCGATCATCAGCACAT
 4 hCoV-19/Beijing/105/2020|EPI_ISL_413518|2020-01-26                   82.6%  79.9%     TTGACAGGACACGAGTAACTCGTCTATCTTCTGCAGGCTGCTTACGGTTTCGTCCGTGTTGCAGCCGATCATCAGCACAT
 5 hCoV-19/Belgium/BA-02291/2020|EPI_ISL_415159|2020-02-29              90.9%  90.2%     TTGACAGGACACGAGTAACTCGTCTATCTTCTGCAGGCTGCTTACGGTTTCGTCCGTGTTGCAGCCGATCATCAGCACAT
 6 hCoV-19/Brazil/AMBR-02/2020|EPI_ISL_417034|2020-03-16               100.0%  98.0%     TTGACAGGACACGAGTAACTCGTCTATCTTCTGCAGGCTGCTTACGGTTTCGTCCGTGTTGCAGCCGATCATCAGCACAT
 7 hCoV-19/Cambodia/0012/2020|EPI_ISL_411902|2020-01-27                 82.2%  80.2%     TTGACAGGACACGAGTAACTCGTCTATCTTCTGCAGGCTGCTTACGGTTTCGTCCGTGTTGCAGCCGATCATCAGCACAT
 8 hCoV-19/Canada/BC_02421/2020|EPI_ISL_415581|2020-03-01               97.9%  96.3%     TTGACAGGACACGAGTAACTCGTCTATCTTCTGCAGGCTGCTTACGGTTTCGTCCGTGTTGCAGCCGATCATCAGCACAT
 9 hCoV-19/Chile/Santiago_op2d1/2020|EPI_ISL_415658|2020-03-06          97.5%  97.9%     TTGACAGGACACGAGTAACTCGTCTATCTTCTGCAGGCTGCTTACGGTTTCGTCCGTGTTGCAGCCGATCATCAGCACAT
10 hCoV-19/China/IQTC01/2020|EPI_ISL_412966|2020-02-05                  81.0%  78.6%     TTGACAGGACACGAGTAACTCGTCTATCTTCTGCAGGCTGCTTACGGTTTCGTCCGTGTTGCAGCCGATCATCAGCACAT
11 hCoV-19/China/WHU02/2020|EPI_ISL_406717|2020-01-02                   83.1%  80.3%     TTGACAGGACACGAGTAACTCGTCTATCTTCTGCAGGCTGCTTACGGTTTCGTCCGTGTTGCAGCCGATCATCAGCACAT
12 hCoV-19/Chongqing/IVDC-CQ-001/2020|EPI_ISL_408481|2020-01-18         81.4%  82.0%     TTGACAGGACACGAGTAACTCGTCTATCTTCTGCAGGCTGCTTACGGTTTCGTCCGTGTTGCAGCCGATCATCAGCACAT
13 hCoV-19/Congo/108/2020|EPI_ISL_417948|2020-03-19                     85.1%  82.1%     TTGACAGGACACGAGTAACTCGTCTATCTTCTGCAGGCTGCTTACGGTTTCGTCCGTGTTGCAGCCGATCATCAGCACAT
14 hCoV-19/Denmark/SSI-01/2020|EPI_ISL_416142|2020-02-26                91.7%  90.2%     TTGACAGGACACGAGTAACTCGTCTATCTTCTGCAGGCTGCTTACGGTTTCGTCCGTGTTGCAGCCGATCATCAGCACAT
15 hCoV-19/England/01/2020|EPI_ISL_407071|2020-01-29                    85.1%  82.0%     TTGACAGGACACGAGTAACTCGTCTATCTTCTGCAGGCTGCTTACGGTTTCGTCCGTGTTGCAGCCGATCATCAGCACAT
16 hCoV-19/Finland/1/2020|EPI_ISL_407079|2020-01-29                     88.4%  84.9%     TTGACAGGACACGAGTAACTCGTCTATCTTCTGCAGGCTGCTTACGGTTTCGTCCGTGTTGCAGCCGATCATCAGCACAT
17 hCoV-19/Foshan/20SF207/2020|EPI_ISL_406534|2020-01-22                90.9%  89.0%     TTGACAGGACACGAGTAACTCGTCTATCTTCTGCAGGCTGCTTACGGTTTCGTCCGTGTTGCAGCCGATCATCAGCACAT
18 hCoV-19/France/ARA094100/2020|EPI_ISL_418412|2020-03-15              85.5%  84.8%     TTGACAGGACACGAGTAACTCGTCTATCTTCTGCAGGCTGCTTACGGTTTCGTCCGTGTTGCAGCCGATCATCAGCACAT
19 hCoV-19/Fujian/13/2020|EPI_ISL_411066|2020-01-22                     83.5%  80.1%     TTGACAGGACACGAGTAACTCGTCTATCTTCTGCAGGCTGCTTACGGTTTCGTCCGTGTTGCAGCCGATCATCAGCACAT
20 hCoV-19/Georgia/Tb/2020|EPI_ISL_416482|2020-03-13                    84.3%  81.6%     TTGACAGGACACGAGTAACTCGTCTATCTTCTGCAGGCTGCTTACGGTTTCGTCCGTGTTGCAGCCGATCATCAGCACAT
21 hCoV-19/Germany/Baden-Wuerttemberg-1/2020|EPI_ISL_412912|2020-02-25  95.0%  99.1%     TTGACAGGACACGAGTAACTCGTCTATCTTCTGCAGGCTGCTTACGGTTTCGTCCGTGTTGCAGCCGATCATCAGCACAT
22 hCoV-19/Germany/BavPat1/2020|EPI_ISL_406862|2020-01-28               97.9%  96.7%     TTGACAGGACACGAGTAACTCGTCTATCTTCTGCAGGCTGCTTACGGTTTCGTCCGTGTTGCAGCCGATCATCAGCACAT
23 hCoV-19/Germany/NRW-01/2020|EPI_ISL_413488|2020-02-28                97.9%  95.9%     TTGACAGGACACGAGTAACTCGTCTATCTTCTGCAGGCTGCTTACGGTTTCGTCCGTGTTGCAGCCGATCATCAGCACAT
24 hCoV-19/Greece/10/2020|EPI_ISL_418263|2020-03-18                     83.5%  80.5%     TTGACAGGACACGAGTAACTCGTCTATCTTCTGCAGGCTGCTTACGGTTTCGTCCGTGTTGCAGCCGATCATCAGCACAT
25 hCoV-19/Guangdong/2020XN4243-P0035/2020|EPI_ISL_413853|2020-01-30    85.5%  88.0%     TTGACAGGACACGAGTAACTCGTCTATCTTCTGCAGGCTGCTTACGGTTTCGTCCGTGTTGCAGCCGATCATCAGCACAT
26 hCoV-19/Guangzhou/20SF206/2020|EPI_ISL_406533|2020-01-22             89.7%  88.9%     TTGACAGGACACGAGTAACTCGTCTATCTTCTGCAGGCTGCTTACGGTTTCGTCCGTGTTGCAGCCGATCATCAGCACAT
27 hCoV-19/Hangzhou/HZ-1/2020|EPI_ISL_406970|2020-01-20                 83.1%  81.0%     TTGACAGGACACGAGTAACTCGTCTATCTTCTGCAGGCTGCTTACGGTTTCGTCCGTGTTGCAGCCGATCATCAGCACAT
28 hCoV-19/Hefei/2/2020|EPI_ISL_412026|2020-02-23                       84.3%  80.2%     TTGACAGGACACGAGTAACTCGTCTATCTTCTGCAGGCTGCTTACGGTTTCGTCCGTGTTGCAGCCGATCATCAGCACAT
29 hCoV-19/Hong_Kong/Case1_21012020/2020|EPI_ISL_417176|2020-01-21      97.5%  99.6%     TTGACAGGACACGAGTAACTCGTCTATCTTCTGCAGGCTGCTTACGGTTTCGTCCGTGTTGCAGCCGATCATCAGCACAT
30 hCoV-19/Hungary/2/2020|EPI_ISL_418183|2020-03-17                     97.9%  94.4%     TTGACAGGACACGAGTAACTCGTCTATCTTCTGCAGGCTGCTTACGGTTTCGTCCGTGTTGCAGCCGATCATCAGCACAT
31 hCoV-19/Iceland/1/2020|EPI_ISL_417535|2020-03-13                     87.2%  84.1%     TTGACAGGACACGAGTAACTCGTCTATCTTCTGCAGGCTGCTTACGGTTTCGTCCGTGTTGCAGCCGATCATCAGCACAT
32 hCoV-19/India/1-31/2020|EPI_ISL_413523|2020-01-31                    91.3%  88.0%     TTGACAGGACACGAGTAACTCGTCTATCTTCTGCAGGCTGCTTACGGTTTCGTCCGTGTTGCAGCCGATCATCAGCACAT
33 hCoV-19/Ireland/21023/2020|EPI_ISL_418516|2020-03-06                 81.8%  78.1%     TTGACAGGACACGAGTAACTCGTCTATCTTCTGCAGGCTGCTTACGGTTTCGTCCGTGTTGCAGCCGATCATCAGCACAT
34 hCoV-19/Israel/ISR_IT0320/2020|EPI_ISL_419210|2020-03                79.8%  78.5%     TTGACAGGACACGAGTAACTCGTCTATCTTCTGCAGGCTGCTTACGGTTTCGTCCGTGTTGCAGCCGATCATCAGCACAT
35 hCoV-19/Italy/SPL1/2020|EPI_ISL_412974|2020-01-29                    83.1%  80.0%     TTGACAGGACACGAGTAACTCGTCTATCTTCTGCAGGCTGCTTACGGTTTCGTCCGTGTTGCAGCCGATCATCAGCACAT
36 hCoV-19/Japan/DP0005/2020|EPI_ISL_416565|2020-02-15                  88.4%  85.9%     TTGACAGGACACGAGTAACTCGTCTATCTTCTGCAGGCTGCTTACGGTTTCGTCCGTGTTGCAGCCGATCATCAGCACAT
37 hCoV-19/Jiangsu/JS01/2020|EPI_ISL_411950|2020-01-23                  82.2%  79.8%     TTGACAGGACACGAGTAACTCGTCTATCTTCTGCAGGCTGCTTACGGTTTCGTCCGTGTTGCAGCCGATCATCAGCACAT
38 hCoV-19/Jiangsu/JS02/2020|EPI_ISL_411952|2020-01-24                  82.2%  79.8%     TTGACAGGACACGAGTAACTCGTCTATCTTCTGCAGGCTGCTTACGGTTTCGTCCGTGTTGCAGCCGATCATCAGCACAT
39 hCoV-19/Jiangxi/IVDC-JX-002/2020|EPI_ISL_408486|2020-01-11           85.5%  85.5%     TTGACAGGACACGAGTAACTCGTCTATCTTCTGCAGGCTGCTTACGGTTTCGTCCGTGTTGCAGCCGATCATCAGCACAT
40 hCoV-19/Jingzhou/HBCDC-HB-01/2020|EPI_ISL_412459|2020-01-08          83.5%  83.8%     TTGACAGGACACGAGTAACTCGTCTATCTTCTGCAGGCTGCTTACGGTTTCGTCCGTGTTGCAGCCGATCATCAGCACAT
41 hCoV-19/Kuwait/KU09/2020|EPI_ISL_416541|2020-03-02                   83.1%  80.3%     TTGACAGGACACGAGTAACTCGTCTATCTTCTGCAGGCTGCTTACGGTTTCGTCCGTGTTGCAGCCGATCATCAGCACAT
42 hCoV-19/Lithuania/ChVir1632/2020|EPI_ISL_416741|2020-02              85.1%  84.4%     TTGACAGGACACGAGTAACTCGTCTATCTTCTGCAGGCTGCTTACGGTTTCGTCCGTGTTGCAGCCGATCATCAGCACAT
43 hCoV-19/Luxembourg/LNS0158952/2020|EPI_ISL_417534|2020-03-18         78.5%  79.5%     TTGACAGGACACGAGTAACTCGTCTATCTTCTGCAGGCTGCTTACGGTTTCGTCCGTGTTGCAGCCGATCATCAGCACAT
44 hCoV-19/Malaysia/188407/2020|EPI_ISL_417918|2020-03-18               95.5%  93.9%     TTGACAGGACACGAGTAACTCGTCTATCTTCTGCAGGCTGCTTACGGTTTCGTCCGTGTTGCAGCCGATCATCAGCACAT
45 hCoV-19/Mexico/CDMX-InDRE_01/2020|EPI_ISL_412972|2020-02-27          78.9%  79.6%     TTGACAGGACACGAGTAACTCGTCTATCTTCTGCAGGCTGCTTACGGTTTCGTCCGTGTTGCAGCCGATCATCAGCACAT
46 hCoV-19/NanChang/JX216/2020|EPI_ISL_417420|2020-03-23                91.7%  90.2%     TTGACAGGACACGAGTAACTCGTCTATCTTCTGCAGGCTGCTTACGGTTTCGTCCGTGTTGCAGCCGATCATCAGCACAT
47 hCoV-19/Nepal/61/2020|EPI_ISL_410301|2020-01-13                      90.1%  86.1%     TTGACAGGACACGAGTAACTCGTCTATCTTCTGCAGGCTGCTTACGGTTTCGTCCGTGTTGCAGCCGATCATCAGCACAT
48 hCoV-19/Netherlands/Utrecht_1/2020|EPI_ISL_414435|2020-03-03         96.3%  97.5%     TTGACAGGACACGAGTAACTCGTCTATCTTCTGCAGGCTGCTTACGGTTTCGTCCGTGTTGCAGCCGATCATCAGCACAT
49 hCoV-19/New_Zealand/01/2020|EPI_ISL_413490|2020-02-27               97.9%  95.9%     TTGACAGGACACGAGTAACTCGTCTATCTTCTGCAGGCTGCTTACGGTTTCGTCCGTGTTGCAGCCGATCATCAGCACAT
50 hCoV-19/Nonthaburi/61/2020|EPI_ISL_403962|2020-01-08                 86.4%  84.2%     TTGACAGGACACGAGTAACTCGTCTATCTTCTGCAGGCTGCTTACGGTTTCGTCCGTGTTGCAGCCGATCATCAGCACAT
51 hCoV-19/Norway/1379/2020|EPI_ISL_417483|2020-02-27                   85.5%  82.7%     TTGACAGGACACGAGTAACTCGTCTATCTTCTGCAGGCTGCTTACGGTTTCGTCCGTGTTGCAGCCGATCATCAGCACAT
52 hCoV-19/Pakistan/Gilgit1/2020|EPI_ISL_417444|2020-03-04              95.5%  94.7%     TTGACAGGACACGAGTAACTCGTCTATCTTCTGCAGGCTGCTTACGGTTTCGTCCGTGTTGCAGCCGATCATCAGCACAT
53 hCoV-19/Panama/328677/2020|EPI_ISL_415152|2020-03-06                 81.8%  79.8%     TTGACAGGACACGAGTAACTCGTCTATCTTCTGCAGGCTGCTTACGGTTTCGTCCGTGTTGCAGCCGATCATCAGCACAT
54 hCoV-19/pangolin/Guangdong/1/2019|EPI_ISL_410721|2019                81.4%  78.9%     TTGACAGGACACGAGTAACTCGTCTATCTTCTGCAGGCTGCTTACGGTTTCGTCCGTGTTGCAGCCGATCATCAGCATAC
55 hCoV-19/Peru/010/2020|EPI_ISL_415787|2020-03-10                      87.6%  84.1%     TTGACAGGACACGAGTAACTCGTCTATCTTCTGCAGGCTGCTTACGGTTTCGTCCGTGTTGCAGCCGATCATCAGCACAT
56 hCoV-19/Poland/PL_P1/2020|EPI_ISL_416488|2020-03-03                  82.2%  79.4%     TTGACAGGACACGAGTAACTCGTCTATCTTCTGCAGGCTGCTTACAGTTTCGTCCGTGTTGCAGCCGATCATCAGCACAT
57 hCoV-19/Portugal/CV62/2020|EPI_ISL_413647|2020-03-01                 81.8%  80.2%     TTGACAGGACACGAGTAACTCGTCTATCTTCTGCAGGCTGCTTACGGTTTCGTCCGTGTTGCAGCCGATCATCAGCACAT
58 hCoV-19/Russia/StPetersburg-3524/2020|EPI_ISL_415710|2020-03-15      77.3%  79.2%     TTGACAGGACACGAGTAACTCGTCTATCTTCTGCAGGCTGCTTACGGTTTCGTCCGTGTTGCAGCCGATCATCAGCACAT
59 hCoV-19/Saudi_Arabia/KAIMRC-Alghoribi/2020|EPI_ISL_416432|2020-03-07 72.7%  76.2%     TTGACAGGACACGAGTAACTCGTCTATCTTCTGCAGGCTGCTTACGGTTTCGTCCGTGTTGCAGCCGATCATC-------
60 hCoV-19/Scotland/CVR05/2020|EPI_ISL_414027|2020-03-04                97.9%  96.3%     TTGACAGGACACGAGTAACTCGTCTATCTTCTGCAGGCTGCTTACGGTTTCGTCCGTGTTGCAGCCGATCATCAGCACAT
61 hCoV-19/Senegal/003/2020|EPI_ISL_418206|2020-02-28                   96.7%  95.5%     TTGACAGGACACGAGTAACTCGTCTATCTTCTGCAGGCTGCTTACGGTTTCGTCCGTGTTGCAGCCGATCATCAGCACAT
62 hCoV-19/Shandong/IVDC-SD-001/2020|EPI_ISL_408482|2020-01-19          83.9%  84.2%     TTGACAGGACACGAGTAACTCGTCTATCTTCTGCAGGCTGCTTACGGTTTCGTCCGTGTTGCAGCCGATCATCAGCACAT
63 hCoV-19/Shanghai/SH0002/2020|EPI_ISL_416316|2020-01-25               93.4%  91.8%     TTGACAGGACACGAGTAACTCGTCTATCTTCTGCAGGCTGCTTACGGTTTCGTCCGTGTTGCAGCCGATCATCAGCACAT
64 hCoV-19/Shenzhen/HKU-SZ-002/2020|EPI_ISL_406030|2020-01-10           92.6%  92.5%     TTGACAGGACACGAGTAACTCGTCTATCTTCTGCAGGCTGCTTACGGTTTCGTCCGTGTTGCAGCCGATCATCAGCACAT
65 hCoV-19/Sichuan/IVDC-SC-001/2020|EPI_ISL_408484|2020-01-15           85.5%  85.5%     TTGACAGGACACGAGTAACTCGTCTATCTTCTGCAGGCTGCTTACGGTTTCGTCCGTGTTGCAGCCGATCATCAGCACAT
66 hCoV-19/Singapore/1/2020|EPI_ISL_406973|2020-01-23                   82.6%  79.9%     TTGACAGGACACGAGTAACTCGTCTATCTTCTGCAGGCTGCTTACGGTTTCGTCCGTGTTGCAGCCGATCATCAGCACAT
67 hCoV-19/Slovakia/SK-BMC1/2020|EPI_ISL_417877|2020-03-06              81.4%  80.7%     TTGACAGGACACGAGTAACTCGTCTATCTTCTGCAGGCTGCTTACGGTTTCGTCCGTGTTGCAGCCGATCATCAGCACAT
68 hCoV-19/South_Africa/R03006-20/2020|EPI_ISL_417186|2020-03-07        78.1%  79.4%     TTGACAGGACACGAGTAACTCGTCTATCTTCTGCAGGCTGCTTACGGTTTCGTCCGTGTTGCAGCCGATCATCAGCACAT
69 hCoV-19/South_Korea/KCDC03/2020|EPI_ISL_407193|2020-01-25            91.3%  90.9%     TTGACAGGACACGAGTAACTCGTCTATCTTCTGCAGGCTGCTTACGGTTTCGTCCGTGTTGCAGCCGATCATCAGCACAT
70 hCoV-19/Spain/Madrid_H10_39/2020|EPI_ISL_417963|2020-03-12           95.9%  96.3%     TTGACAGGACACGAGTAACTCGTCTATCTTCTGCAGGCTGCTTACGGTTTCGTCCGTGTTGCAGCCGATCATCAGCACAT
71 hCoV-19/Sweden/01/2020|EPI_ISL_411951|2020-02-07                     83.5%  80.1%     TTGACAGGACACGAGTAACTCGTCTATCTTCTGCAGGCTGCTTACGGTTTCGTCCGTGTTGCAGCCGATCATCAGCACAT
72 hCoV-19/Switzerland/1000477377/2020|EPI_ISL_413020|2020-02-27        78.1%  79.4%     TTGACAGGACACGAGTAACTCGTCTATCTTCTGCAGGCTGCTTACGGTTTCGTCCGTGTTGCAGCCGATCATCAGCACAT
73 hCoV-19/Taiwan/2/2020|EPI_ISL_406031|2020-01-23                      85.1%  81.3%     TTGACAGGACACGAGTAACTCGTCTATCTTCTGCAGGCTGCTTACGGTTTCGTCCGTGTTGCAGCCGATCATCAGCACAT
74 hCoV-19/USA/AZ1/2020|EPI_ISL_406223|2020-01-22                       84.3%  80.2%     TTGACAGGACACGAGTAACTCGTCTATCTTCTGCAGGCTGCTTACGGTTTCGTCCGTGTTGCAGCCGATCATCAGCACAT
75 hCoV-19/Vietnam/19-01S/2020|EPI_ISL_418269|2020-01-22                81.4%  79.7%     TTGACAGGACACGAGTAACTCGTCTATCTTCTGCAGGCTGCTTACGGTTTCGTCCGTGTTGCAGCCGATCATCAGCACAT
76 hCoV-19/Wales/PHW04/2020|EPI_ISL_415041|2020-03-07                   97.9%  95.2%     TTGACAGGACACGAGTAACTCGTCTATCTTCTGCAGGCTGCTTACGGTTTCGTCCGTGTTGCAGCCGATCATCAGCACAT
77 hCoV-19/Wuhan/HBCDC-HB-01/2019|EPI_ISL_402132|2019-12-30             84.7%  84.0%     TTGACAGGACACGAGTAACTCGTCTATCTTCTGCAGGCTGCTTACGGTTTCGTCCGTGTTGCAGCCGATCATCAGCACAT
78 hCoV-19/Wuhan-Hu-1/2019|EPI_ISL_402125|2019-12-31                    83.1%  80.0%     TTGACAGGACACGAGTAACTCGTCTATCTTCTGCAGGCTGCTTACGGTTTCGTCCGTGTTGCAGCCGATCATCAGCACAT
79 hCoV-19/Yunnan/IVDC-YN-003/2020|EPI_ISL_408480|2020-01-17            82.2%  81.8%     TTGACAGGACACGAGTAACTCGTCTATCTTCTGCAGGCTGCTTACGGTTTCGTCCGTGTTGCAGCCGATCATCAGCACAT
80 hCoV-19/Zhejiang/WZ-01/2020|EPI_ISL_404227|2020-01-16                89.3%  87.4%     TTGACAGGACACGAGTAACTCGTCTATCTTCTGCAGGCTGCTTACGGTTTCGTCCGTGTTGCAGCCGATCATCAGCACAT
   consensus/100%                                                                        TTGACAGGACACGAGTAACTCuTCTATCTTCTGCAGGsTGCTTACuGTTTCGTCCGTGTTGCAGCCGATCATC.......
   consensus/90%                                                                         TTGACAGGACACGAGTAACTCGTCTATCTTCTGCAGGCTGCTTACGGTTTCGTCCGTGTTGCAGCCGATCATCAGCACAT
   consensus/80%                                                                         TTGACAGGACACGAGTAACTCGTCTATCTTCTGCAGGCTGCTTACGGTTTCGTCCGTGTTGCAGCCGATCATCAGCACAT
   consensus/70%                                                                         TTGACAGGACACGAGTAACTCGTCTATCTTCTGCAGGCTGCTTACGGTTTCGTCCGTGTTGCAGCCGATCATCAGCACAT

                                                                          cov    pid 241          :         .         .         .         .         3         .         . 320
 1 hCoV-19/Algeria/G0638_2264/2020|EPI_ISL_418241|2020-03-02           100.0% 100.0%     CTAGGTTTTGTCCGGGTGTGACCGAAAGGTAAGATGGAGAGCCTTGTCCCTGGTTTCAA---------------------
 2 hCoV-19/Australia/NSW01/2020|EPI_ISL_407893|2020-01-24               97.9%  96.3%     CTAGGTTTCGTCCGGGTGTGACCGAAAGGTAAGATGGAGAGCCTTGTCCCTGGTTTCAACGAGAAAA-------------
 3 hCoV-19/bat/Yunnan/RaTG13/2013|EPI_ISL_402131|2013-07-24             86.4%  84.4%     CTAGGTTTCGTCCGGGTGTGACCGAA------------------------------------------------------
 4 hCoV-19/Beijing/105/2020|EPI_ISL_413518|2020-01-26                   82.6%  79.9%     CTAGGTTTCGTCCGGGT---------------------------------------------------------------
 5 hCoV-19/Belgium/BA-02291/2020|EPI_ISL_415159|2020-02-29              90.9%  90.2%     CTAGGTTTTGTCCGGGTGTGACCGAAAGGTAAGATGG-------------------------------------------
 6 hCoV-19/Brazil/AMBR-02/2020|EPI_ISL_417034|2020-03-16               100.0%  98.0%     CTAGGTTTCGTCCGGGTGTGACCGAAAGGTAAGATGGAGAGCCTTGTCCCTGGTTTCAAC--------------------
 7 hCoV-19/Cambodia/0012/2020|EPI_ISL_411902|2020-01-27                 82.2%  80.2%     CTAGGTTTCGTCCGGG----------------------------------------------------------------
 8 hCoV-19/Canada/BC_02421/2020|EPI_ISL_415581|2020-03-01               97.9%  96.3%     CTAGGTTTCGTCCGGGTGTGACCGAAAGGTAAGATGGAGAGCCTTGTCCCTGGTTTCAACGAGAAAA-------------
 9 hCoV-19/Chile/Santiago_op2d1/2020|EPI_ISL_415658|2020-03-06          97.5%  97.9%     CTAGGTTTCGTCCGGGTGTGACCGAAAGGTAAGATGGAGAGCCTTGTCCCTGGTTTCAACGAG-----------------
10 hCoV-19/China/IQTC01/2020|EPI_ISL_412966|2020-02-05                  81.0%  78.6%     CTAGGTTTCGTCC-------------------------------------------------------------------
11 hCoV-19/China/WHU02/2020|EPI_ISL_406717|2020-01-02                   83.1%  80.3%     CTAGGTTTCGTCCGGGTG--------------------------------------------------------------
```

```
12 hCoV-19/Chongqing/IVDC-CQ-001/2020|EPI_ISL_408481|2020-01-18          81.4%  82.0%   CTAGGTTTCGTCCG------------------------------------------------------------------
13 hCoV-19/Congo/108/2020|EPI_ISL_417948|2020-03-19                      85.1%  82.1%   CTAGGTTTTGTCCGGGTGTGACC---------------------------------------------------------
14 hCoV-19/Denmark/SSI-01/2020|EPI_ISL_416142|2020-02-26                 91.7%  90.2%   CTAGGTTTTGTCCGGGTGTGACCGAAAGGTAAGATGGAG-----------------------------------------
15 hCoV-19/England/01/2020|EPI_ISL_407071|2020-01-29                     85.1%  82.0%   CTAGGTTTCGTCCGGGTGTGACC---------------------------------------------------------
16 hCoV-19/Finland/1/2020|EPI_ISL_407079|2020-01-29                      88.4%  84.9%   CTAGGTTTCGTCCGGGTGTGACCGAAAGGTA-------------------------------------------------
17 hCoV-19/Foshan/20SF207/2020|EPI_ISL_406534|2020-01-22                 90.9%  89.0%   CTAGGTTTCGTCCGGGTGTGACCGAAAGGTAAGATGG-------------------------------------------
18 hCoV-19/France/ARA094100/2020|EPI_ISL_418412|2020-03-15               85.5%  84.8%   CTAGGTTTTGTCCGGGTGTGACCG--------------------------------------------------------
19 hCoV-19/Fujian/13/2020|EPI_ISL_411066|2020-01-22                      83.5%  80.1%   CTAGGTTTCGTCCGGGTGT-------------------------------------------------------------
20 hCoV-19/Georgia/Tb/2020|EPI_ISL_416482|2020-03-13                     84.3%  81.6%   CTAGGTTTTGTCCGGGTGTGA-----------------------------------------------------------
21 hCoV-19/Germany/Baden-Wuerttemberg-1/2020|EPI_ISL_412912|2020-02-25   95.0%  99.1%   CTAGGTTTTGTCCGGGTGTGACCGAAAGGTAAGATGGAGAGCCTTGT---------------------------------
22 hCoV-19/Germany/BavPat1/2020|EPI_ISL_406862|2020-01-28                97.9%  96.7%   CTAGGTTTTGTCCGGGTGTGACCGAAAGGTAAGATGGAGAGCCTTGTCCCTGGTTTCAACGAGAAAA-------------
23 hCoV-19/Germany/NRW-01/2020|EPI_ISL_413488|2020-02-28                 97.9%  95.9%   CTAGGTTTCGTCCGGGTGTGACCGAAAGGTAAGATGGAGAGCCTTGTCCCTGGTTTCAACGAGAAAAC------------
24 hCoV-19/Greece/10/2020|EPI_ISL_418263|2020-03-18                      83.5%  80.5%   CTAGGTTTTGTCCGGGTGT-------------------------------------------------------------
25 hCoV-19/Guangdong/2020XN4243-P0035/2020|EPI_ISL_413853|2020-01-30     85.5%  88.0%   CTAGGTTTCGTCCGGGTGTGACCG--------------------------------------------------------
26 hCoV-19/Guangzhou/20SF206/2020|EPI_ISL_406533|2020-01-22              89.7%  88.9%   CTAGGTTTCGTCCGGGTGTGACCGAAAGGTAAGA---------------------------------------------
27 hCoV-19/Hangzhou/HZ-1/2020|EPI_ISL_406970|2020-01-20                  83.1%  81.0%   CTAGGTTTCGTCCGGGTG--------------------------------------------------------------
28 hCoV-19/Hefei/2/2020|EPI_ISL_412026|2020-02-23                        84.3%  80.2%   CTAGGTTTCGTCCGGGTGTGA-----------------------------------------------------------
29 hCoV-19/Hong_Kong/Case1_21012020/2020|EPI_ISL_417176|2020-01-21       97.5%  99.6%   CTAGGTTTCGTCCGGGTGTGACCGAAAGGTAAGATGGAGAGCCTTGTCCCTGGTTTCA----------------------
30 hCoV-19/Hungary/2/2020|EPI_ISL_418183|2020-03-17                      97.9%  94.4%   CTAGGTTTTGTCCGGGTGTGACCGAAAGGTAAGATGGAGAGCCTTGTCCCTGGTTTCAACGAGAAAACACACG-------
31 hCoV-19/Iceland/1/2020|EPI_ISL_417535|2020-03-13                      87.2%  84.1%   CTAGGTTTTGTCCGGGTGTGACCGAAAG----------------------------------------------------
32 hCoV-19/India/1-31/2020|EPI_ISL_413523|2020-01-31                     91.3%  88.0%   CTAGGTTTCGTCCGGGTGTGACCGAAAGGTAAGATGGA------------------------------------------
33 hCoV-19/Ireland/21023/2020|EPI_ISL_418516|2020-03-06                  81.8%  78.1%   CTAGGTTTTGTCCGG-----------------------------------------------------------------
34 hCoV-19/Israel/ISR_IT0320/2020|EPI_ISL_419210|2020-03                 79.8%  78.5%   CTAGGTTTTG----------------------------------------------------------------------
35 hCoV-19/Italy/SPL1/2020|EPI_ISL_412974|2020-01-29                     83.1%  80.0%   CTAGGTTTCGTCCGGGTG--------------------------------------------------------------
36 hCoV-19/Japan/DP0005/2020|EPI_ISL_416565|2020-02-15                   88.4%  85.9%   CTAGGTTTCGTCCGGGTGTGACCGAAAGGTAAGATGGAGAGCCTTGTCCCTGGTTTCAACGAGAAAACACACGTCCAACT
37 hCoV-19/Jiangsu/JS01/2020|EPI_ISL_411950|2020-01-23                   82.2%  79.8%   CTAGGTTTCGTCCGGG----------------------------------------------------------------
38 hCoV-19/Jiangsu/JS02/2020|EPI_ISL_411952|2020-01-24                   82.2%  79.8%   CTAGGTTTCGTCCGGG----------------------------------------------------------------
39 hCoV-19/Jiangxi/IVDC-JX-002/2020|EPI_ISL_408486|2020-01-11            85.5%  85.5%   CTAGGTTTCGTCCGGGTGTGACCG--------------------------------------------------------
40 hCoV-19/Jingzhou/HBCDC-HB-01/2020|EPI_ISL_412459|2020-01-08           83.5%  83.8%   CTAGGTTTCGTCCGGGTGT-------------------------------------------------------------
41 hCoV-19/Kuwait/KU09/2020|EPI_ISL_416541|2020-03-02                    83.1%  80.3%   CTAGGTTTCGTCCGGGTG--------------------------------------------------------------
42 hCoV-19/Lithuania/ChVir1632/2020|EPI_ISL_416741|2020-02               85.1%  84.4%   CTAGGTTTTGTCCGGGTGTGACC---------------------------------------------------------
43 hCoV-19/Luxembourg/LNS0158952/2020|EPI_ISL_417534|2020-03-18          78.5%  79.5%   CTAGGTT-------------------------------------------------------------------------
44 hCoV-19/Malaysia/188407/2020|EPI_ISL_417918|2020-03-18                95.5%  93.9%   CTAGGTTTCGTCCGGGTGTGACCGAAAGGTAAGATGGAGAGCCTTGTC--------------------------------
45 hCoV-19/Mexico/CDMX-InDRE_01/2020|EPI_ISL_412972|2020-02-27           78.9%  79.6%   CTAGGTTT------------------------------------------------------------------------
46 hCoV-19/NanChang/JX216/2020|EPI_ISL_417420|2020-03-23                 91.7%  90.2%   CTAGGTTTTGTCCGGGTGTGACCGAAAGGTAAGATGGAG-----------------------------------------
47 hCoV-19/Nepal/61/2020|EPI_ISL_410301|2020-01-13                       90.1%  86.1%   CTAGGTTTCGTCCGGGTGTGACCGAAAGGTAAGAT--------------------------------------------
48 hCoV-19/Netherlands/Utrecht_1/2020|EPI_ISL_414435|2020-03-03          96.3%  97.5%   CTAGGTTTTGTCCGGGTGTGACCGAAAGGTAAGATGGAGAGCCTTGTCCC------------------------------
49 hCoV-19/New_Zealand/01/2020|EPI_ISL_413490|2020-02-27                 97.9%  95.9%   CTAGGTTTCGTCCGGGTGTGACCGAAAGGTAAGATGGAGAGCCTTGTCCCTGGTTTCAACGAGAAAAC------------
50 hCoV-19/Nonthaburi/61/2020|EPI_ISL_403962|2020-01-08                  86.4%  84.2%   CTAGGTTTCGTCCGGGTGTGACCGAA------------------------------------------------------
51 hCoV-19/Norway/1379/2020|EPI_ISL_417483|2020-02-27                    85.5%  82.7%   CTAGGTTTCGTCCGGGTGTGACCG--------------------------------------------------------
52 hCoV-19/Pakistan/Gilgit1/2020|EPI_ISL_417444|2020-03-04               95.5%  94.7%   CTAGGTTTTGTCCGGGTGTGACCGAAAGGTAAGATGGAGAGCCTTGTC--------------------------------
53 hCoV-19/Panama/328677/2020|EPI_ISL_415152|2020-03-06                  81.8%  79.8%   CTAGGTTTTGTCCGG-----------------------------------------------------------------
54 hCoV-19/pangolin/Guangdong/1/2019|EPI_ISL_410721|2019                 81.4%  78.9%   CTAGGTTTCGTCCG------------------------------------------------------------------
55 hCoV-19/Peru/010/2020|EPI_ISL_415787|2020-03-10                       87.6%  84.1%   CTAGGTTTTGTCCGGGTGTGACCGAAAGG---------------------------------------------------
56 hCoV-19/Poland/PL_P1/2020|EPI_ISL_416488|2020-03-03                   82.2%  79.4%   CTAGGTTTCGTCCGGG----------------------------------------------------------------
57 hCoV-19/Portugal/CV62/2020|EPI_ISL_413647|2020-03-01                  81.8%  80.2%   CTAGGTTTTGTCCGG-----------------------------------------------------------------
58 hCoV-19/Russia/StPetersburg-3524/2020|EPI_ISL_415710|2020-03-15       77.3%  79.2%   CTAG----------------------------------------------------------------------------
59 hCoV-19/Saudi_Arabia/KAIMRC-Alghoribi/2020|EPI_ISL_416432|2020-03-07  72.7%  76.2%   --------------------------------------------------------------------------------
60 hCoV-19/Scotland/CVR05/2020|EPI_ISL_414027|2020-03-04                 97.9%  96.3%   CTAGGTTTTGTCCGGGTGTGACCGAAAGGTAAGATGGAGAGCCTTGTCCCTGGTTTCAACGAGAAAAC------------
61 hCoV-19/Senegal/003/2020|EPI_ISL_418206|2020-02-28                    96.7%  95.5%   CTAGGTTTTGTCCGGGTGTGACCGAAAGGTAAGATGGAGAGCCTTGTCCCT-----------------------------
62 hCoV-19/Shandong/IVDC-SD-001/2020|EPI_ISL_408482|2020-01-19           83.9%  84.2%   CTAGGTTTCGTCCGGGTGTG------------------------------------------------------------
63 hCoV-19/Shanghai/SH0002/2020|EPI_ISL_416316|2020-01-25                93.4%  91.8%   CTAGGTTTCGTCCGGGTGTGACCGAAAGGTAAGATGGAGAGCC-------------------------------------
64 hCoV-19/Shenzhen/HKU-SZ-002/2020|EPI_ISL_406030|2020-01-10            92.6%  92.5%   CTAGGTTTCGTCCGGGTGTGACCGAAAGGTAAGATGGAGAG--------------------------------------
65 hCoV-19/Sichuan/IVDC-SC-001/2020|EPI_ISL_408484|2020-01-15            85.5%  85.5%   CTAGGTTTCGTCCGGGTGTGACCG--------------------------------------------------------
66 hCoV-19/Singapore/1/2020|EPI_ISL_406973|2020-01-23                    82.6%  79.9%   CTAGGTTTCGTCCGGGT---------------------------------------------------------------
67 hCoV-19/Slovakia/SK-BMC1/2020|EPI_ISL_417877|2020-03-06               81.4%  80.7%   CTAGGTTTTGTCCG------------------------------------------------------------------
68 hCoV-19/South_Africa/R03006-20/2020|EPI_ISL_417186|2020-03-07         78.1%  79.4%   CTAGGT--------------------------------------------------------------------------
69 hCoV-19/South_Korea/KCDC03/2020|EPI_ISL_407193|2020-01-25             91.3%  90.9%   CTAGGTTTCGTCCGGGTGTGACCGAAAGGTAAGATGGA------------------------------------------
70 hCoV-19/Spain/Madrid_H10_39/2020|EPI_ISL_417963|2020-03-12            95.9%  96.3%   CTAGGTTTTGTCCGGGTGTGACCGAAAGGTAAGATGGAGAGCCTTGTCC-------------------------------
71 hCoV-19/Sweden/01/2020|EPI_ISL_411951|2020-02-07                      83.5%  80.1%   CTAGGTTTCGTCCGGGTGT-------------------------------------------------------------
72 hCoV-19/Switzerland/1000477377/2020|EPI_ISL_413020|2020-02-27         78.1%  79.4%   CTAGGT--------------------------------------------------------------------------
73 hCoV-19/Taiwan/2/2020|EPI_ISL_406031|2020-01-23                       85.1%  81.3%   CTAGGTTTCGTCCGGGTGTGACC---------------------------------------------------------
74 hCoV-19/USA/AZ1/2020|EPI_ISL_406223|2020-01-22                        84.3%  80.2%   CTAGGTTTCGTCCGGGTGTGA-----------------------------------------------------------
75 hCoV-19/Vietnam/19-01S/2020|EPI_ISL_418269|2020-01-22                 81.4%  79.7%   CTAGGTTTCGTCCG------------------------------------------------------------------
76 hCoV-19/Wales/PHW04/2020|EPI_ISL_415041|2020-03-07                    97.9%  95.2%   CTAGGTTTTGTCCGGGTGTGACCGAAAGGTAAGATGGAGAGCCTTGTCCCTGGTTTCAACGAGAAAACACA---------
77 hCoV-19/Wuhan/HBCDC-HB-01/2019|EPI_ISL_402132|2019-12-30              84.7%  84.0%   CTAGGTTTCGTCCGGGTGTGAC----------------------------------------------------------
78 hCoV-19/Wuhan-Hu-1/2019|EPI_ISL_402125|2019-12-31                     83.1%  80.0%   CTAGGTTTCGTCCGGGTG--------------------------------------------------------------
79 hCoV-19/Yunnan/IVDC-YN-003/2020|EPI_ISL_408480|2020-01-17             82.2%  81.8%   CTAGGTTTCGTCCGGG----------------------------------------------------------------
80 hCoV-19/Zhejiang/WZ-01/2020|EPI_ISL_404227|2020-01-16                 89.3%  87.4%   CTAGGTTTCGTCCGGGTGTGACCGAAAGGTAAG----------------------------------------------
   consensus/100%                                                                       ................................................................................
   consensus/90%                                                                        CTAGGTTTsGTCCG..................................................................
   consensus/80%                                                                        CTAGGTTTsGTCCGGG................................................................
   consensus/70%                                                                        CTAGGTTTsGTCCGGGTG..............................................................

                                                                          cov    pid 321        .         .         :         .         .         .         .        4 400
 1 hCoV-19/Algeria/G0638_2264/2020|EPI_ISL_418241|2020-03-02             100.0% 100.0%   --------------------------------------------------------------------------------
 2 hCoV-19/Australia/NSW01/2020|EPI_ISL_407893|2020-01-24                 97.9%  96.3%   --------------------------------------------------------------------------------
 3 hCoV-19/bat/Yunnan/RaTG13/2013|EPI_ISL_402131|2013-07-24               86.4%  84.4%   --------------------------------------------------------------------------------
 4 hCoV-19/Beijing/105/2020|EPI_ISL_413518|2020-01-26                     82.6%  79.9%   --------------------------------------------------------------------------------
 5 hCoV-19/Belgium/BA-02291/2020|EPI_ISL_415159|2020-02-29                90.9%  90.2%   --------------------------------------------------------------------------------
 6 hCoV-19/Brazil/AMBR-02/2020|EPI_ISL_417034|2020-03-16                 100.0%  98.0%   --------------------------------------------------------------------------------
 7 hCoV-19/Cambodia/0012/2020|EPI_ISL_411902|2020-01-27                   82.2%  80.2%   --------------------------------------------------------------------------------
 8 hCoV-19/Canada/BC_02421/2020|EPI_ISL_415581|2020-03-01                 97.9%  96.3%   --------------------------------------------------------------------------------
 9 hCoV-19/Chile/Santiago_op2d1/2020|EPI_ISL_415658|2020-03-06            97.5%  97.9%   --------------------------------------------------------------------------------
10 hCoV-19/China/IQTC01/2020|EPI_ISL_412966|2020-02-05                    81.0%  78.6%   --------------------------------------------------------------------------------
11 hCoV-19/China/WHU02/2020|EPI_ISL_406717|2020-01-02                     83.1%  80.3%   --------------------------------------------------------------------------------
12 hCoV-19/Chongqing/IVDC-CQ-001/2020|EPI_ISL_408481|2020-01-18           81.4%  82.0%   --------------------------------------------------------------------------------
13 hCoV-19/Congo/108/2020|EPI_ISL_417948|2020-03-19                       85.1%  82.1%   --------------------------------------------------------------------------------
14 hCoV-19/Denmark/SSI-01/2020|EPI_ISL_416142|2020-02-26                  91.7%  90.2%   --------------------------------------------------------------------------------
15 hCoV-19/England/01/2020|EPI_ISL_407071|2020-01-29                      85.1%  82.0%   --------------------------------------------------------------------------------
16 hCoV-19/Finland/1/2020|EPI_ISL_407079|2020-01-29                       88.4%  84.9%   --------------------------------------------------------------------------------
17 hCoV-19/Foshan/20SF207/2020|EPI_ISL_406534|2020-01-22                  90.9%  89.0%   --------------------------------------------------------------------------------
18 hCoV-19/France/ARA094100/2020|EPI_ISL_418412|2020-03-15                85.5%  84.8%   --------------------------------------------------------------------------------
19 hCoV-19/Fujian/13/2020|EPI_ISL_411066|2020-01-22                       83.5%  80.1%   --------------------------------------------------------------------------------
20 hCoV-19/Georgia/Tb/2020|EPI_ISL_416482|2020-03-13                      84.3%  81.6%   --------------------------------------------------------------------------------
21 hCoV-19/Germany/Baden-Wuerttemberg-1/2020|EPI_ISL_412912|2020-02-25    95.0%  99.1%   --------------------------------------------------------------------------------
22 hCoV-19/Germany/BavPat1/2020|EPI_ISL_406862|2020-01-28                 97.9%  96.7%   --------------------------------------------------------------------------------
23 hCoV-19/Germany/NRW-01/2020|EPI_ISL_413488|2020-02-28                  97.9%  95.9%   --------------------------------------------------------------------------------
24 hCoV-19/Greece/10/2020|EPI_ISL_418263|2020-03-18                       83.5%  80.5%   --------------------------------------------------------------------------------
25 hCoV-19/Guangdong/2020XN4243-P0035/2020|EPI_ISL_413853|2020-01-30      85.5%  88.0%   --------------------------------------------------------------------------------
26 hCoV-19/Guangzhou/20SF206/2020|EPI_ISL_406533|2020-01-22               89.7%  88.9%   --------------------------------------------------------------------------------
27 hCoV-19/Hangzhou/HZ-1/2020|EPI_ISL_406970|2020-01-20                   83.1%  81.0%   --------------------------------------------------------------------------------
28 hCoV-19/Hefei/2/2020|EPI_ISL_412026|2020-02-23                         84.3%  80.2%   --------------------------------------------------------------------------------
29 hCoV-19/Hong_Kong/Case1_21012020/2020|EPI_ISL_417176|2020-01-21        97.5%  99.6%   --------------------------------------------------------------------------------
30 hCoV-19/Hungary/2/2020|EPI_ISL_418183|2020-03-17                       97.9%  94.4%   --------------------------------------------------------------------------------
31 hCoV-19/Iceland/1/2020|EPI_ISL_417535|2020-03-13                       87.2%  84.1%   --------------------------------------------------------------------------------
32 hCoV-19/India/1-31/2020|EPI_ISL_413523|2020-01-31                      91.3%  88.0%   --------------------------------------------------------------------------------
33 hCoV-19/Ireland/21023/2020|EPI_ISL_418516|2020-03-06                   81.8%  78.1%   --------------------------------------------------------------------------------
34 hCoV-19/Israel/ISR_IT0320/2020|EPI_ISL_419210|2020-03                  79.8%  78.5%   --------------------------------------------------------------------------------
35 hCoV-19/Italy/SPL1/2020|EPI_ISL_412974|2020-01-29                      83.1%  80.0%   --------------------------------------------------------------------------------
36 hCoV-19/Japan/DP0005/2020|EPI_ISL_416565|2020-02-15                    88.4%  85.9%   CAGTTTGCCTGTT-------------------------------------------------------------------
37 hCoV-19/Jiangsu/JS01/2020|EPI_ISL_411950|2020-01-23                    82.2%  79.8%   --------------------------------------------------------------------------------
38 hCoV-19/Jiangsu/JS02/2020|EPI_ISL_411952|2020-01-24                    82.2%  79.8%   --------------------------------------------------------------------------------
39 hCoV-19/Jiangxi/IVDC-JX-002/2020|EPI_ISL_408486|2020-01-11             85.5%  85.5%   --------------------------------------------------------------------------------
40 hCoV-19/Jingzhou/HBCDC-HB-01/2020|EPI_ISL_412459|2020-01-08            83.5%  83.8%   --------------------------------------------------------------------------------
41 hCoV-19/Kuwait/KU09/2020|EPI_ISL_416541|2020-03-02                     83.1%  80.3%   --------------------------------------------------------------------------------
42 hCoV-19/Lithuania/ChVir1632/2020|EPI_ISL_416741|2020-02                85.1%  84.4%   --------------------------------------------------------------------------------
43 hCoV-19/Luxembourg/LNS0158952/2020|EPI_ISL_417534|2020-03-18           78.5%  79.5%   --------------------------------------------------------------------------------
44 hCoV-19/Malaysia/188407/2020|EPI_ISL_417918|2020-03-18                 95.5%  93.9%   --------------------------------------------------------------------------------
45 hCoV-19/Mexico/CDMX-InDRE_01/2020|EPI_ISL_412972|2020-02-27            78.9%  79.6%   --------------------------------------------------------------------------------
46 hCoV-19/NanChang/JX216/2020|EPI_ISL_417420|2020-03-23                  91.7%  90.2%   --------------------------------------------------------------------------------
47 hCoV-19/Nepal/61/2020|EPI_ISL_410301|2020-01-13                        90.1%  86.1%   --------------------------------------------------------------------------------
48 hCoV-19/Netherlands/Utrecht_1/2020|EPI_ISL_414435|2020-03-03           96.3%  97.5%   --------------------------------------------------------------------------------
49 hCoV-19/New_Zealand/01/2020|EPI_ISL_413490|2020-02-27                  97.9%  95.9%   --------------------------------------------------------------------------------
50 hCoV-19/Nonthaburi/61/2020|EPI_ISL_403962|2020-01-08                   86.4%  84.2%   --------------------------------------------------------------------------------
51 hCoV-19/Norway/1379/2020|EPI_ISL_417483|2020-02-27                     85.5%  82.7%   --------------------------------------------------------------------------------
52 hCoV-19/Pakistan/Gilgit1/2020|EPI_ISL_417444|2020-03-04                95.5%  94.7%   --------------------------------------------------------------------------------
53 hCoV-19/Panama/328677/2020|EPI_ISL_415152|2020-03-06                   81.8%  79.8%   --------------------------------------------------------------------------------
54 hCoV-19/pangolin/Guangdong/1/2019|EPI_ISL_410721|2019                  81.4%  78.9%   --------------------------------------------------------------------------------
55 hCoV-19/Peru/010/2020|EPI_ISL_415787|2020-03-10                        87.6%  84.1%   --------------------------------------------------------------------------------
56 hCoV-19/Poland/PL_P1/2020|EPI_ISL_416488|2020-03-03                    82.2%  79.4%   --------------------------------------------------------------------------------
57 hCoV-19/Portugal/CV62/2020|EPI_ISL_413647|2020-03-01                   81.8%  80.2%   --------------------------------------------------------------------------------
58 hCoV-19/Russia/StPetersburg-3524/2020|EPI_ISL_415710|2020-03-15        77.3%  79.2%   --------------------------------------------------------------------------------
59 hCoV-19/Saudi_Arabia/KAIMRC-Alghoribi/2020|EPI_ISL_416432|2020-03-07   72.7%  76.2%   --------------------------------------------------------------------------------
60 hCoV-19/Scotland/CVR05/2020|EPI_ISL_414027|2020-03-04                  97.9%  96.3%   --------------------------------------------------------------------------------
61 hCoV-19/Senegal/003/2020|EPI_ISL_418206|2020-02-28                     96.7%  95.5%   --------------------------------------------------------------------------------
62 hCoV-19/Shandong/IVDC-SD-001/2020|EPI_ISL_408482|2020-01-19            83.9%  84.2%   --------------------------------------------------------------------------------
63 hCoV-19/Shanghai/SH0002/2020|EPI_ISL_416316|2020-01-25                 93.4%  91.8%   --------------------------------------------------------------------------------
```

```
64 hCoV-19/Shenzhen/HKU-SZ-002/2020|EPI_ISL_406030|2020-01-10             92.6%  92.5%     --------------------------------------------------------------------------------
65 hCoV-19/Sichuan/IVDC-SC-001/2020|EPI_ISL_408484|2020-01-15             85.5%  85.5%     --------------------------------------------------------------------------------
66 hCoV-19/Singapore/1/2020|EPI_ISL_406973|2020-01-23                     82.6%  79.9%     --------------------------------------------------------------------------------
67 hCoV-19/Slovakia/SK-BMC1/2020|EPI_ISL_417877|2020-03-06                81.4%  80.7%     --------------------------------------------------------------------------------
68 hCoV-19/South_Africa/R03006-20/2020|EPI_ISL_417186|2020-03-07          78.1%  79.4%     --------------------------------------------------------------------------------
69 hCoV-19/South_Korea/KCDC03/2020|EPI_ISL_407193|2020-01-25              91.3%  90.9%     --------------------------------------------------------------------------------
70 hCoV-19/Spain/Madrid_H10_39/2020|EPI_ISL_417963|2020-03-12             95.9%  96.3%     --------------------------------------------------------------------------------
71 hCoV-19/Sweden/01/2020|EPI_ISL_411951|2020-02-07                       83.5%  80.1%     --------------------------------------------------------------------------------
72 hCoV-19/Switzerland/1000477377/2020|EPI_ISL_413020|2020-02-27          78.1%  79.4%     --------------------------------------------------------------------------------
73 hCoV-19/Taiwan/2/2020|EPI_ISL_406031|2020-01-23                        85.1%  81.3%     --------------------------------------------------------------------------------
74 hCoV-19/USA/AZ1/2020|EPI_ISL_406223|2020-01-22                         84.3%  80.2%     --------------------------------------------------------------------------------
75 hCoV-19/Vietnam/19-01S/2020|EPI_ISL_418269|2020-01-22                  81.4%  79.7%     --------------------------------------------------------------------------------
76 hCoV-19/Wales/PHW04/2020|EPI_ISL_415041|2020-03-07                     97.9%  95.2%     --------------------------------------------------------------------------------
77 hCoV-19/Wuhan/HBCDC-HB-01/2019|EPI_ISL_402132|2019-12-30               84.7%  84.0%     --------------------------------------------------------------------------------
78 hCoV-19/Wuhan-Hu-1/2019|EPI_ISL_402125|2019-12-31                      83.1%  80.0%     --------------------------------------------------------------------------------
79 hCoV-19/Yunnan/IVDC-YN-003/2020|EPI_ISL_408480|2020-01-17              82.2%  81.8%     --------------------------------------------------------------------------------
80 hCoV-19/Zhejiang/WZ-01/2020|EPI_ISL_404227|2020-01-16                  89.3%  87.4%     --------------------------------------------------------------------------------
   consensus/100%                                                                          ................................................................................
   consensus/90%                                                                           ................................................................................
   consensus/80%                                                                           ................................................................................
   consensus/70%                                                                           ................................................................................

                                                                            cov    pid 401   ] 403
 1 hCoV-19/Algeria/G0638_2264/2020|EPI_ISL_418241|2020-03-02             100.0% 100.0%     ---
 2 hCoV-19/Australia/NSW01/2020|EPI_ISL_407893|2020-01-24                 97.9%  96.3%     ---
 3 hCoV-19/bat/Yunnan/RaTG13/2013|EPI_ISL_402131|2013-07-24               86.4%  84.4%     ---
 4 hCoV-19/Beijing/105/2020|EPI_ISL_413518|2020-01-26                     82.6%  79.9%     ---
 5 hCoV-19/Belgium/BA-02291/2020|EPI_ISL_415159|2020-02-29                90.9%  90.2%     ---
 6 hCoV-19/Brazil/AMBR-02/2020|EPI_ISL_417034|2020-03-16                 100.0%  98.0%     ---
 7 hCoV-19/Cambodia/0012/2020|EPI_ISL_411902|2020-01-27                   82.2%  80.2%     ---
 8 hCoV-19/Canada/BC_02421/2020|EPI_ISL_415581|2020-03-01                 97.9%  96.3%     ---
 9 hCoV-19/Chile/Santiago_op2d1/2020|EPI_ISL_415658|2020-03-06            97.5%  97.9%     ---
10 hCoV-19/China/IQTC01/2020|EPI_ISL_412966|2020-02-05                    81.0%  78.6%     ---
11 hCoV-19/China/WHU02/2020|EPI_ISL_406717|2020-01-02                     83.1%  80.3%     ---
12 hCoV-19/Chongqing/IVDC-CQ-001/2020|EPI_ISL_408481|2020-01-18           81.4%  82.0%     ---
13 hCoV-19/Congo/108/2020|EPI_ISL_417948|2020-03-19                       85.1%  82.1%     ---
14 hCoV-19/Denmark/SSI-01/2020|EPI_ISL_416142|2020-02-26                  91.7%  90.2%     ---
15 hCoV-19/England/01/2020|EPI_ISL_407071|2020-01-29                      85.1%  82.0%     ---
16 hCoV-19/Finland/1/2020|EPI_ISL_407079|2020-01-29                       88.4%  84.9%     ---
17 hCoV-19/Foshan/20SF207/2020|EPI_ISL_406534|2020-01-22                  90.9%  89.0%     ---
18 hCoV-19/France/ARA094100/2020|EPI_ISL_418412|2020-03-15                85.5%  84.8%     ---
19 hCoV-19/Fujian/13/2020|EPI_ISL_411066|2020-01-22                       83.5%  80.1%     ---
20 hCoV-19/Georgia/Tb/2020|EPI_ISL_416482|2020-03-13                      84.3%  81.6%     ---
21 hCoV-19/Germany/Baden-Wuerttemberg-1/2020|EPI_ISL_412912|2020-02-25    95.0%  99.1%     ---
22 hCoV-19/Germany/BavPat1/2020|EPI_ISL_406862|2020-01-28                 97.9%  96.7%     ---
23 hCoV-19/Germany/NRW-01/2020|EPI_ISL_413488|2020-02-28                  97.9%  95.9%     ---
24 hCoV-19/Greece/10/2020|EPI_ISL_418263|2020-03-18                       83.5%  80.5%     ---
25 hCoV-19/Guangdong/2020XN4243-P0035/2020|EPI_ISL_413853|2020-01-30      85.5%  88.0%     ---
26 hCoV-19/Guangzhou/20SF206/2020|EPI_ISL_406533|2020-01-22               89.7%  88.9%     ---
27 hCoV-19/Hangzhou/HZ-1/2020|EPI_ISL_406970|2020-01-20                   83.1%  81.0%     ---
28 hCoV-19/Hefei/2/2020|EPI_ISL_412026|2020-02-23                         84.3%  80.2%     ---
29 hCoV-19/Hong_Kong/Case1_21012020/2020|EPI_ISL_417176|2020-01-21        97.5%  99.6%     ---
30 hCoV-19/Hungary/2/2020|EPI_ISL_418183|2020-03-17                       97.9%  94.4%     ---
31 hCoV-19/Iceland/1/2020|EPI_ISL_417535|2020-03-13                       87.2%  84.1%     ---
32 hCoV-19/India/1-31/2020|EPI_ISL_413523|2020-01-31                      91.3%  88.0%     ---
33 hCoV-19/Ireland/21023/2020|EPI_ISL_418516|2020-03-06                   81.8%  78.1%     ---
34 hCoV-19/Israel/ISR_IT0320/2020|EPI_ISL_419210|2020-03                  79.8%  78.5%     ---
35 hCoV-19/Italy/SPL1/2020|EPI_ISL_412974|2020-01-29                      83.1%  80.0%     ---
36 hCoV-19/Japan/DP0005/2020|EPI_ISL_416565|2020-02-15                    88.4%  85.9%     ---
37 hCoV-19/Jiangsu/JS01/2020|EPI_ISL_411950|2020-01-23                    82.2%  79.8%     ---
38 hCoV-19/Jiangsu/JS02/2020|EPI_ISL_411952|2020-01-24                    82.2%  79.8%     ---
39 hCoV-19/Jiangxi/IVDC-JX-002/2020|EPI_ISL_408486|2020-01-11             85.5%  85.5%     ---
40 hCoV-19/Jingzhou/HBCDC-HB-01/2020|EPI_ISL_412459|2020-01-08            83.5%  83.8%     ---
41 hCoV-19/Kuwait/KU09/2020|EPI_ISL_416541|2020-03-02                     83.1%  80.3%     ---
42 hCoV-19/Lithuania/ChVir1632/2020|EPI_ISL_416741|2020-02                85.1%  84.4%     ---
43 hCoV-19/Luxembourg/LNS0158952/2020|EPI_ISL_417534|2020-03-18           78.5%  79.5%     ---
44 hCoV-19/Malaysia/188407/2020|EPI_ISL_417918|2020-03-18                 95.5%  93.9%     ---
45 hCoV-19/Mexico/CDMX-InDRE_01/2020|EPI_ISL_412972|2020-02-27            78.9%  79.6%     ---
46 hCoV-19/NanChang/JX216/2020|EPI_ISL_417420|2020-03-23                  91.7%  90.2%     ---
47 hCoV-19/Nepal/61/2020|EPI_ISL_410301|2020-01-13                        90.1%  86.1%     ---
48 hCoV-19/Netherlands/Utrecht_1/2020|EPI_ISL_414435|2020-03-03           96.3%  97.5%     ---
49 hCoV-19/New_Zealand/01/2020|EPI_ISL_413490|2020-02-27                  97.9%  95.9%     ---
50 hCoV-19/Nonthaburi/61/2020|EPI_ISL_403962|2020-01-08                   86.4%  84.2%     ---
51 hCoV-19/Norway/1379/2020|EPI_ISL_417483|2020-02-27                     85.5%  82.7%     ---
52 hCoV-19/Pakistan/Gilgit1/2020|EPI_ISL_417444|2020-03-04                95.5%  94.7%     ---
53 hCoV-19/Panama/328677/2020|EPI_ISL_415152|2020-03-06                   81.8%  79.8%     ---
54 hCoV-19/pangolin/Guangdong/1/2019|EPI_ISL_410721|2019                  81.4%  78.9%     ---
55 hCoV-19/Peru/010/2020|EPI_ISL_415787|2020-03-10                        87.6%  84.1%     ---
56 hCoV-19/Poland/PL_P1/2020|EPI_ISL_416488|2020-03-03                    82.2%  79.4%     ---
57 hCoV-19/Portugal/CV62/2020|EPI_ISL_413647|2020-03-01                   81.8%  80.2%     ---
58 hCoV-19/Russia/StPetersburg-3524/2020|EPI_ISL_415710|2020-03-15        77.3%  79.2%     ---
59 hCoV-19/Saudi_Arabia/KAIMRC-Alghoribi/2020|EPI_ISL_416432|2020-03-07   72.7%  76.2%     ---
60 hCoV-19/Scotland/CVR05/2020|EPI_ISL_414027|2020-03-04                  97.9%  96.3%     ---
61 hCoV-19/Senegal/003/2020|EPI_ISL_418206|2020-02-28                     96.7%  95.5%     ---
62 hCoV-19/Shandong/IVDC-SD-001/2020|EPI_ISL_408482|2020-01-19            83.9%  84.2%     ---
63 hCoV-19/Shanghai/SH0002/2020|EPI_ISL_416316|2020-01-25                 93.4%  91.8%     ---
64 hCoV-19/Shenzhen/HKU-SZ-002/2020|EPI_ISL_406030|2020-01-10             92.6%  92.5%     ---
65 hCoV-19/Sichuan/IVDC-SC-001/2020|EPI_ISL_408484|2020-01-15             85.5%  85.5%     ---
66 hCoV-19/Singapore/1/2020|EPI_ISL_406973|2020-01-23                     82.6%  79.9%     ---
67 hCoV-19/Slovakia/SK-BMC1/2020|EPI_ISL_417877|2020-03-06                81.4%  80.7%     ---
68 hCoV-19/South_Africa/R03006-20/2020|EPI_ISL_417186|2020-03-07          78.1%  79.4%     ---
69 hCoV-19/South_Korea/KCDC03/2020|EPI_ISL_407193|2020-01-25              91.3%  90.9%     ---
70 hCoV-19/Spain/Madrid_H10_39/2020|EPI_ISL_417963|2020-03-12             95.9%  96.3%     ---
71 hCoV-19/Sweden/01/2020|EPI_ISL_411951|2020-02-07                       83.5%  80.1%     ---
72 hCoV-19/Switzerland/1000477377/2020|EPI_ISL_413020|2020-02-27          78.1%  79.4%     ---
73 hCoV-19/Taiwan/2/2020|EPI_ISL_406031|2020-01-23                        85.1%  81.3%     ---
74 hCoV-19/USA/AZ1/2020|EPI_ISL_406223|2020-01-22                         84.3%  80.2%     ---
75 hCoV-19/Vietnam/19-01S/2020|EPI_ISL_418269|2020-01-22                  81.4%  79.7%     ---
76 hCoV-19/Wales/PHW04/2020|EPI_ISL_415041|2020-03-07                     97.9%  95.2%     ---
77 hCoV-19/Wuhan/HBCDC-HB-01/2019|EPI_ISL_402132|2019-12-30               84.7%  84.0%     ---
78 hCoV-19/Wuhan-Hu-1/2019|EPI_ISL_402125|2019-12-31                      83.1%  80.0%     ---
79 hCoV-19/Yunnan/IVDC-YN-003/2020|EPI_ISL_408480|2020-01-17              82.2%  81.8%     ---
80 hCoV-19/Zhejiang/WZ-01/2020|EPI_ISL_404227|2020-01-16                  89.3%  87.4%     ---
   consensus/100%                                                                          ...
   consensus/90%                                                                           ...
   consensus/80%                                                                           ...
   consensus/70%                                                                           ...
```

MView 1.63, Copyright © 1997-2018 Nigel P. Brown
