# Supplementary figures and images for: Potential use of noncoding RNAs and innovative therapeutic strategies to target the 5’UTR of SARS-CoV-2
Source: Epigenomics. 2020 Sep 2:10.2217/epi-2020-0162. doi: 10.2217/epi-2020-0162 (PMC7466951; doi:10.2217/epi-2020-0162)

## 5'UTR of all SARS-CoV isolates

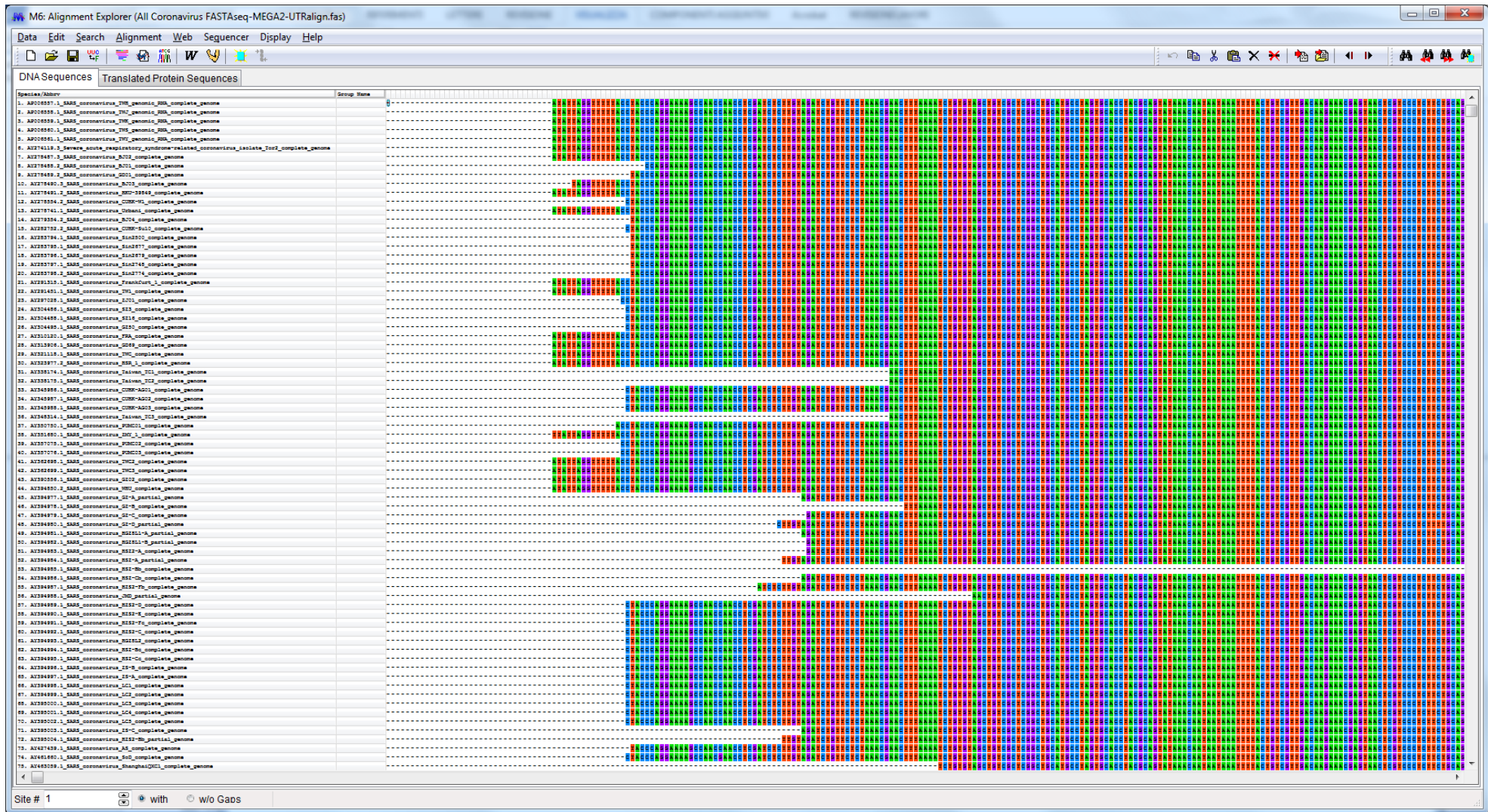

## 5'UTR of all SARS-CoV-2 isolates

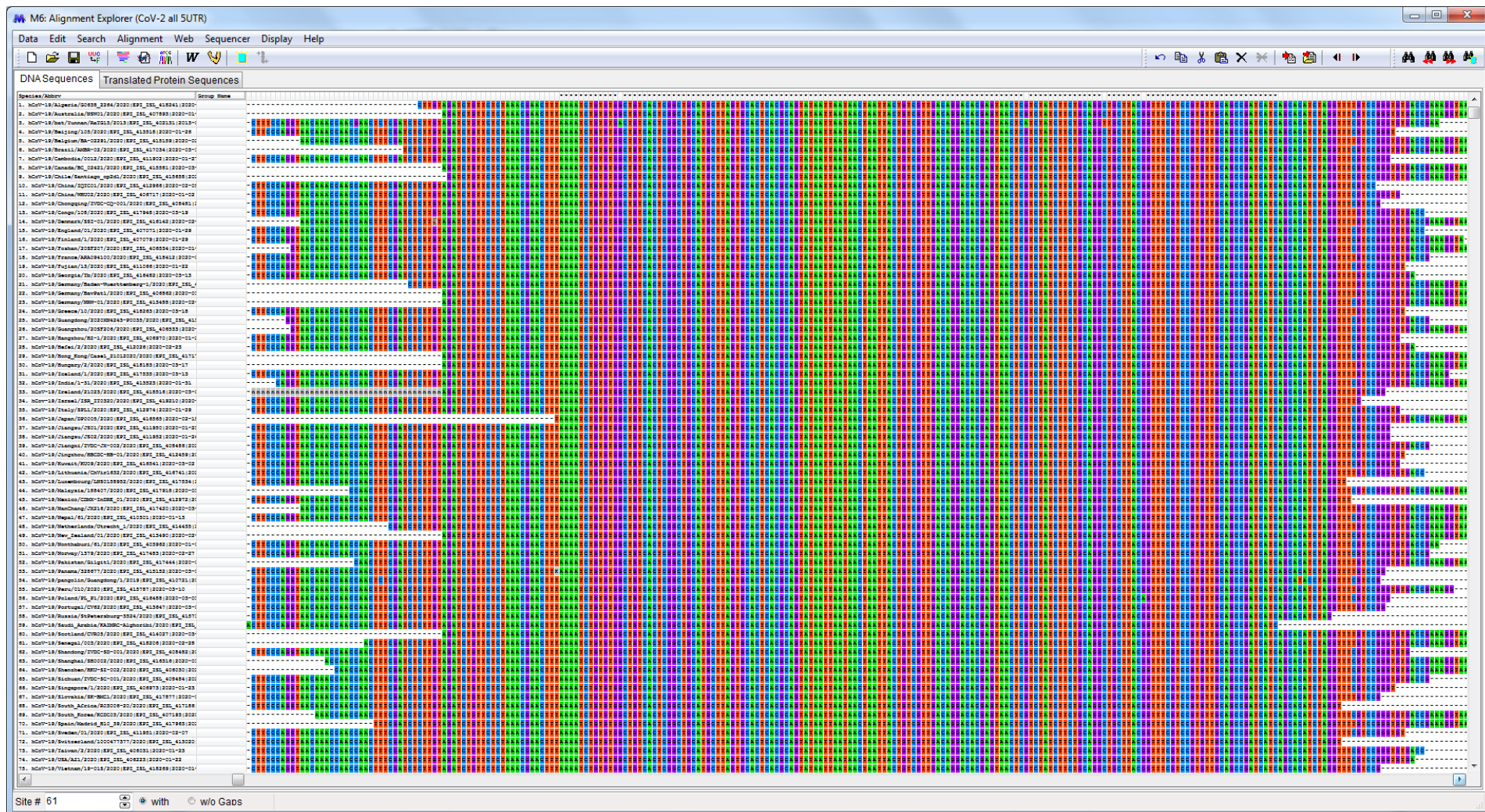

Supplement: Supplementary file 3 [file epi-2020-0162-supp-table-3.pdf]
